# Risk of Major Congenital Malformations Following Prenatal Exposure to Smoking Cessation Medicines

**DOI:** 10.1001/jamainternmed.2025.0290

**Published:** 2025-03-31

**Authors:** Duong T. Tran, Jacqueline M. Cohen, Sarah Donald, Carolyn E. Cesta, Kari Furu, Lianne Parkin, Sallie-Anne Pearson, Johan Reutfors, Annelies L. Robijn, Helga Zoega, Nicholas Zwar, Alys Havard

**Affiliations:** 1 National Drug and Alcohol Research Centre, Faculty of Medicine and Health, University of New South Wales, Sydney, New South Wales, Australia; 2 Department of Chronic Diseases, Norwegian Institute of Public Health, Oslo, Norway; 3 Centre for Fertility and Health, Norwegian Institute of Public Health, Oslo, Norway; 4 Department of Preventive and Social Medicine, Dunedin School of Medicine, University of Otago, Dunedin, New Zealand; 5 Centre for Pharmacoepidemiology, Department of Medicine Solna, Karolinska Institute, Stockholm, Sweden; 6 Medicines Intelligence Research Program, School of Population Health, Faculty of Medicine and Health, University of New South Wales, Sydney, New South Wales, Australia; 7 Centre of Public Health Sciences, Faculty of Medicine, University of Iceland, Reykjavik, Iceland; 8 Faculty of Health Sciences & Medicine, Bond University, Gold Coast, Queensland, Australia

## Abstract

**Importance:**

Nicotine replacement therapy (NRT), varenicline, and bupropion are effective smoking cessation pharmacotherapies, but evidence on fetal safety is limited.

**Objective:**

To assess whether prenatal use of smoking cessation pharmacotherapies was associated with increased risks of major congenital malformations (MCMs).

**Design, Setting, and Participants:**

This retrospective cohort study was conducted across 4 countries, and results were pooled via meta-analyses. Records of all births (2001-2020) in New South Wales (NSW; Australia), New Zealand (NZ), Norway, and Sweden were linked to prescribed medicine dispensings, hospital admission, outpatient, and death data. Follow-up ended December 31, 2021, and the data were analyzed between August and October 2023. The base cohort comprised 391 474 infants among 267 522 women who smoked during the first trimester or were dispensed a smoking cessation pharmacotherapy 90 days before conception or during the first trimester.

**Exposures:**

Supply of NRT, varenicline, and bupropion overlapping the first trimester. Unexposed infants were born to women who smoked but were not dispensed a pharmacotherapy 90 days preconception and the first trimester. Propensity score matching (1:10) was used.

**Main Outcomes and Measures:**

MCM overall and subgroups.

**Results:**

The mean (SD) maternal age at childbirth was 27.2 (6.0) years. Analyses included 9325 infants exposed to NRT (NSW, NZ), 3031 to varenicline (NSW, NZ, Norway, and Sweden), and 1042 to bupropion (NSW, NZ). Compared with unexposed infants, there were no differences in prevalence of MCMs overall following NRT exposure (37.6 vs 34.4 per 1000 live births; adjusted relative risk [aRR], 1.10; 95% CI, 0.98-1.22), varenicline (32.7 vs 36.6; aRR, 0.90; 95% CI, 0.73-1.10), or bupropion (35.5 vs 38.8; aRR, 0.93; 95% CI, 0.67-1.29). NRT analyses showed no difference in the risk of MCMs of the heart, limbs, genital organs, kidney/urinary tract, respiratory system, and orofacial clefts but a higher risk of digestive organ MCMs (3.8 vs 2.5 per 1000 live births; aRR, 1.53; 95% CI, 1.05-2.23; *P* = .41 after multiple comparison adjustment). Varenicline analyses revealed no difference in the risk of heart, limb, and genital MCMs but a higher risk of kidney/urinary tract MCMs (11.5 vs 4.2 per 1000 live births; aRR, 2.75; 95% CI, 1.42-5.34; *P* = .09 after multiple comparison adjustment), with findings for other MCMs being too imprecise. For bupropion, data were too sparse to estimate the risk of MCM subgroups.

**Conclusions and Relevance:**

The results of this cohort study suggest that there is no clear increased risk of MCMs associated with prenatal use of NRT and varenicline compared with smoking during the first trimester.

## Key Points

**Question:**

Is the use of nicotine replacement therapy (NRT), varenicline, and bupropion during the first trimester of pregnancy associated with increased risks of major congenital malformations (MCMs)?

**Findings:**

In this cohort study of 5.2 million births in Australia (state of New South Wales), New Zealand, Norway, and Sweden, there was no increased risk of MCMs overall following exposure to NRT (9325 infants), varenicline (3031 infants), or bupropion (1042 infants). The study found no evidence of higher risk of several malformation subgroups for NRT or varenicline, while the risk of malformation subgroups could not be estimated robustly for bupropion.

**Meaning:**

The results of this study suggest that, considering the harms of smoking, it is reassuring that prenatal use of NRT and varenicline for smoking cessation was not associated with increased risks of MCMs compared with smoking during the first trimester.

## Teaser

This cohort study examines whether prenatal use of smoking cessation pharmacotherapies is associated with increased risks of major congenital malformations.

## Introduction

Smoking causes various adverse pregnancy outcomes, including major congenital malformations (MCMs).^1^ Nicotine replacement therapy (NRT), varenicline (nicotinic acetylcholine receptor partial agonist), and bupropion (nicotinic acetylcholine receptor antagonist) are effective smoking cessation pharmacotherapies that exert their effect by reducing cravings and withdrawal^2^; however, evidence on fetal safety is limited. The Cochrane review^3^ of randomized clinical trials of NRT transdermal patches during pregnancy yielded inconclusive evidence about the potential teratogenicity of NRT due to small samples (total of 696 individuals exposed). Two cohort studies (total of 2927 individuals exposed) found no association between NRT in any formulation and MCM overall but noted a potentially increased risk of musculoskeletal^4^ and respiratory defects.^5^ These cohort studies did not account for maternal smoking, and risk estimates were imprecise, leaving an evidence gap regarding the potential teratogenic effects of NRT. Additionally, to our knowledge, the risk of malformations for other NRT formulations has not been studied. Although fast-acting formulations (eg, gums, lozenges, and inhalers) are preferred for pregnancy because they deliver intermittent and lower daily doses,^2,6^ transdermal patches are more popular.^7^

Evidence regarding the safety of varenicline during pregnancy has been limited to a few observational studies.^8,9,10,11,12^ Two rigorously conducted cohort studies^11,12^ found that varenicline was not associated with an increased risk of MCM overall; however, neither had adequate sample sizes (total of 1031 individuals exposed) to investigate MCM subgroups. Although evidence on bupropion safety during pregnancy exists, the quality is low, and maternal smoking was not considered.^13,14^

The lack of clear evidence hinders attempts to quit smoking with pharmacotherapies during pregnancy. Clinical guidelines recommend that NRT be used with caution and supervision and advise against the use of varenicline and bupropion until further evidence becomes available.^2,3,6^ To overcome the statistical power and confounding by maternal smoking issues present in prior studies, we conducted a large cohort study across 4 countries to assess the risk of MCMs overall and in subgroups and specific malformations associated with maternal use of NRT, varenicline, and bupropion during the first trimester compared with smoking. We also estimated the risk of MCMs separately for NRT transdermal patches and fast-acting formulations.

## Methods

### Data Sources and Study Cohort

We conducted a retrospective cohort study, using a common protocol in New South Wales (NSW, Australia), New Zealand (NZ), Norway, and Sweden. The study received approval from the Australian Institute of Health and Welfare human research ethics committee, New South Wales Population and Health Services research ethics committee, University of Otago human ethics committee , the Norwegian Regional Ethics Committee, and the Swedish ethics committee. In each country, records of all pregnancies resulting in live births and stillbirths (perinatal data) were linked to records of prescription medicine dispensings, hospital admissions, mortality, and specialist health care registers (Norway and Sweden only). The data sources (Figure) contain information on 5 239 070 births among 3 035 489 women. This study followed the Strengthening the Reporting of Observational Studies in Epidemiology (STROBE) reporting guidelines.

**Figure.**
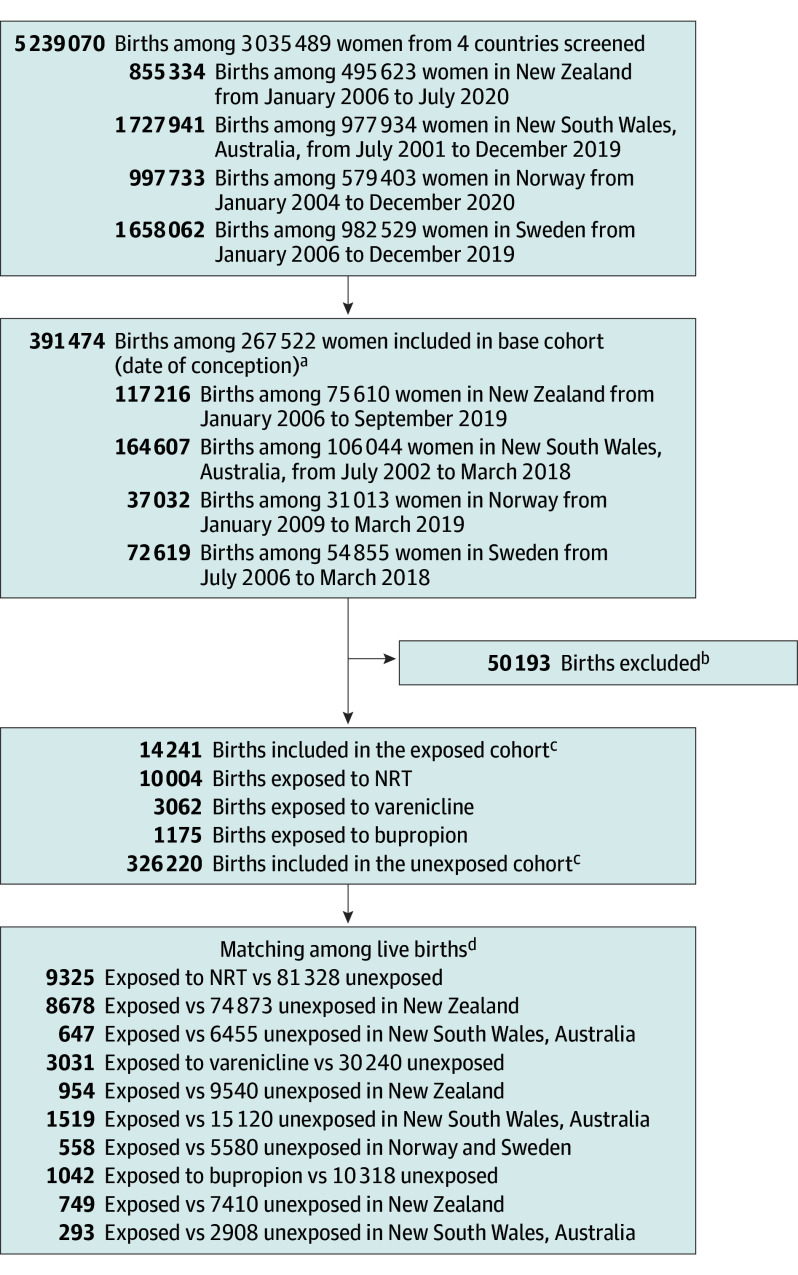
Cohort Selection in New Zealand, Australia (New South Wales), Norway, and Sweden NRT indicates nicotine replacement therapy. ^a^The base cohort included births of women who were dispensed a prescribed smoking cessation pharmacotherapy within 90 days before conception or during the first trimester, plus births of women who self-reported that they smoked during the first trimester. ^b^These included multiple births (n = 10 487); stillbirths (n = 2476); inadequate information to estimate conception (n = 931); a pregnancy interval of shorter than 6 months (n = 17 820); congenital anomalies due to chromosomal anomaly, genetic syndrome, or teratogenic infections (n = 1056); a mother receiving a diagnosis of teratogenic infection during the first trimester or being dispensed potentially or known teratogenic medicines during 90 days preconception and the first trimester (n = 15 128); a mother being dispensed 2 or more smoking cessation pharmacotherapies during the first trimester (n = 285); overseas or interstate visitors (n = 1686); and the infant’s identification missing (n = 324). ^c^The number of exposed infants identified in 4 countries. Unexposed infants were born to women who self-reported that they smoked during the first trimester and were not dispensed a prescribed smoking cessation pharmacotherapy within 90 days before conception and during the first trimester. ^d^Matching on propensity score and year of conception (New Zealand and New South Wales) or year of birth (Norway and Sweden); greedy algorithm, 1 exposed: 10 unexposed, no replacement, caliper 0.25. The number of stillbirths are also presented in eAppendix 8 in Supplement 1.

We identified births with an estimated conception date (DoC; date of childbirth – 7 × weeks of gestation + 14 days) from January 2006 to September 2019 in NZ, July 2002 to March 2018 in NSW, January 2009 to March 2019 in Norway, and July 2006 to March 2018 in Sweden. We then identified births among women who were dispensed smoking cessation pharmacotherapy from 90 days before conception to the end of the first trimester (DoC + 83 days), as well as births of women who smoked during the first trimester (identified via self-report and documented in the perinatal data). This resulted in a base cohort of 391 474 births among 267 522 women (see eAppendices 1 and 2 in Supplement 1 for data sources and a definition of smoking status).

### Exclusions

We excluded multiple births, stillbirths, births with inadequate information to estimate DoC, those conceived within 6 months from prior childbirth, and those having MCMs due to chromosomal anomalies, genetic syndromes, or teratogenic infections. We excluded births to women who had teratogenic viral infection (rubella, cytomegalovirus, or toxoplasmosis) during the first trimester, who were dispensed potentially or known teratogenic medicines within 90 days preconception or during the first trimester, or who were dispensed 2 or more smoking cessation pharmacotherapies during the first trimester. Due to potentially incomplete data, births among overseas visitors (NSW, NZ), interstate visitors (NSW), and a few infants without a linkage identification (NZ) were also excluded (eAppendix 2 in Supplement 1).

### Exposures

NRT is available via prescription and over the counter (OTC) in all participating countries. In NZ, most NRTs (eg, patches, gums, and lozenges) are obtained via prescription and other sources captured by dispensing data (personal communication; eAppendix 2 in Supplement 1), while Australian dispensing data cover only prescription nicotine patches.^7^ In Norway and Sweden, NRT is generally obtained OTC; thus, these countries were not included in the NRT analyses. In all participating countries, varenicline and bupropion are prescription-only medicines and were captured by dispensing data. Our bupropion analyses included births in NZ and Australia, where bupropion is registered only for smoking cessation, but not births in Norway and Sweden, where bupropion is also available for depression treatment. For each dispensing, we calculated days of supply by dividing the quantity supplied by the recommended daily dose.

We defined exposure during the first trimester as the medicine supply overlapping the first trimester (DoC ≤ date of dispensing ≤ DoC + 83 or date of dispensing < DoC ≤ date of dispensing + days of supply). This group comprised women who self-reported that they smoked during the first trimester as well as those who did not. Among the latter, our prior study^7^ analyzing the timing and amount of smoking cessation pharmacotherapy dispensed found that most were likely to smoke during the first trimester (Appendix 2 in Supplement 1). We defined unexposed infants as those born to women who self-reported that they smoked during the first trimester and were not dispensed a prescribed smoking cessation pharmacotherapy during 90 days before conception and the first trimester.

### Outcomes

We defined MCMs according to the EUROCAT, version 1.4, classification,^15^ with minor variations due to national coding practices (eTables 3 and 4 in Supplement 1). The primary outcomes were MCMs overall and MCMs in the following subgroups: heart, limbs, orofacial clefts, genital organs, kidney and urinary tract, digestive system, respiratory system, nervous system, abdominal wall, eye, ear, and others. The secondary outcomes were specific malformations of the heart, limbs, genital organs, kidney and urinary tract, digestive system, nervous system, and orofacial clefts, which were selected for having a prevalence in the general population greater than 10 per 10 000 live births.^16^ We identified MCMs from diagnoses in hospital admissions or cause of death records (within 18 months from birth) for infants born in NZ and NSW. This was supplemented with medical birth registers and outpatient specialist care data (within 12 months of birth) for infants born in Norway or Sweden.

### Covariates

Covariates included year of conception (year of childbirth in the Norway and Sweden cohort), the infant’s sex, the mother’s sociodemographic characteristics (age, self-reported Indigenous status, country of birth, marital status, education level, and remoteness and social deprivation of residential areas), parity, maternal body mass index (calculated as weight in kilograms divided by height in meters squared), a previous child having an MCM, and hospitalizations during the 12 months before conception. Maternal morbidities (mental health disorder, chronic airway disorder, gastroesophageal reflux, use of nonsteroid anti-inflammatory drugs, use of steroids, anemia and coagulation disorders, alcohol or other drug disorders, thyroid disorder, cardiovascular disease, preexisting diabetes, preexisting hypertension, epilepsy, and chronic kidney disease) were identified from perinatal, medicine dispensing, and hospital admission data. Some demographic variables were not available in all countries (eg, Indigenous status was available only in NZ and NSW; see eTables 6 and 7 in Supplement 1 for definitions).

### Statistical Analyses

The unit of analysis was the woman-infant dyad. To adhere to privacy laws, we analyzed unit record data within each country using a common protocol and shared summarized data. The Nordic Pregnancy drug Safety Studies collaboration^17^ enabled us to analyze data from Norway and Sweden as a combined cohort.

We calculated propensity scores using logistic regression models, with exposure status as the dependent variable and covariates as explanatory variables. We matched exposed and unexposed infants on year and propensity score (greedy algorithm, caliper width 0.25, 1 exposed: 10 unexposed, without replacement). We assessed the balance of baseline characteristics, with an absolute standardized difference of less than 0.1 indicating the covariate was balanced between groups.^18^

We calculated MCM prevalence per 1000 live births and 95% CIs. We estimated adjusted relative risks (aRRs) with 95% CIs using univariate conditional Poisson regression models with a robust variance estimator.^19,20^ Analyses were conducted in SAS, version 9.4 (SAS Institute), and Stata, version 17 (StataCorp), with the package *kmatch*.^21^ We assumed 1 underlying true association of the medicine with the risk of MCMs that each country estimates^22,23^; thus, we performed a fixed-effects meta-analysis using the R package *metafor* (R Foundation).^24^ We adjusted for multiple statistical comparisons using the Benjamini-Hochberg method.^25^

We conducted 4 sensitivity analyses for the primary outcomes (eAppendix 8 in Supplement 1). First, as our main analyses assumed all exposed infants born to women who smoked at some point during the first trimester, we restricted the exposed groups to infants of women who self-reported that they smoked during the first trimester. This addressed the possible misclassification of smoking status among women using a pharmacotherapy who quit smoking before conception. For this analysis, smoking quantity was further included in the propensity score. Second, to evaluate the potential bias due to nonuse of dispensed medicines, we restricted exposed groups to women who received 2 or more dispensings, with at least 1 occurring after conception. Third, to examine the potential bias due to inclusion of only live births, we conducted a quantitative bias analysis in which we quantified plausible adjusted RRs under scenarios in which pregnancies with a MCM resulted in miscarriage or termination instead of a livebirth.^25^ Fourth, to examine the potential effects of unmeasured confounding, for MCMs with elevated risks, we calculated the *E* value,^26^ which is the minimum strength of an association that an unmeasured confounder should have with the exposure and outcome to explain away the observed association.^26,27^

## Results

Following exclusions, the propensity score matching yielded 135 284 woman-infant dyads, with 9325 infants exposed to NRT, 3031 to varenicline, and 1042 to bupropion during the first trimester (Figure). The characteristics of exposed and matched unexposed infants were well balanced (Table 1), except for maternal age, Indigenous status, socioeconomic deprivation quintile of maternal residence, and parity and mental health disorders in the NRT analyses for NZ infants; we adjusted for these unbalanced covariates in the final regression models. eTables 8 to 16 in Supplement 1 describe country-specific baseline characteristics before and after propensity score matching.

**Table 1.** Characteristics of Infants Exposed to Nicotine Replacement Therapy (Any Formulation), Varenicline, and Bupropion During the First Trimester and Unexposed Infants Following Propensity Score Matching^a^

Characteristic	No. (%)								
	Nicotine replacement therapy			Varenicline			Bupropion		
	Exposed^b^	Unexposed^b^	Diff^c^	Exposed^b^	Unexposed^b^	Diff^c^	Exposed^b^	Unexposed^b^	Diff^c^
Total, No.	9325	81 328	NA	3031	30 240	NA	1042	10 318	NA
Country, No.									
NZ	8678	74 873	NA	954	9540	NA	749	7410	NA
NSW, Australia	647	6455		1519	15 120		293	2908	
Norway and Sweden	Not available	Not available		558	5580		Not available	Not available	
Infant sex									
Female	4506 (48.3)	39 512 (48.6)	0.01	1450 (47.8)	14 363 (47.5)	0.01	511 (49.0)	4088 (49.3)	0.01
Male	4817 (51.7)	41 794 (51.4)	0.01	1581 (52.2)	15 877 (52.5)	0.01	531 (51.0)	5230 (50.7)	0.01
Maternal age at childbirth, y									
<25	3104 (33.3)	34 412 (42.3)	0.19	632 (20.9)	6224 (20.6)	0.01	217 (20.8)	2627 (25.5)	0.11
25-29	2662 (28.5)	22 403 (27.5)	0.02	931 (30.7)	9538 (31.5)	0.02	320 (30.7)	3169 (30.7)	0.00
30-34	2101 (22.5)	14 706 (18.1)	0.11	831 (27.4)	8275 (27.4)	0.00	300 (28.8)	2739 (26.5)	0.05
≥35	1458 (15.6)	9807 (12.1)	0.10	637 (21.0)	6203 (20.5)	0.01	205 (19.7)	1783 (17.3)	0.06
Indigenous mother^d^	4387 (47.0)	49 994 (61.5)	0.29	609 (24.6)	6003 (24.3)	0.01	338 (32.4)	3568 (34.6)	0.05
Maternal country of birth, same as country in which gave birth^e^	590 (91.2)	5989 (92.8)	0.06	1753 (84.4)	17 655 (85.3)	0.02	255 (87.0)	2569 (88.3)	0.04
Maternal relationship, with a partner^e^	347 (53.6)	3474 (53.8)	0.00	1097 (52.8)	11 102 (53.6)	0.01	201 (68.6)	2076 (71.4)	0.06
Socioeconomic deprivation of residence, quintile^f^									
First (least disadvantage)	923 (9.9)	6038 (7.4)	0.09	528 (21.4)	5130 (20.8)	0.01	147 (14.1)	1299 (12.6)	0.04
Second	1340 (14.4)	9072 (11.2)	0.10	581 (23.5)	5921 (24.0)	0.01	190 (18.2)	1809 (17.5)	0.02
Third	1648 (17.7)	12 141 (14.9)	0.07	471 (19.0)	4666 (18.9)	0.00	201 (19.3)	1955 (18.9)	0.01
Fourth	2407 (25.8)	19 723 (24.3)	0.04	431 (17.4)	4339 (17.6)	0.00	220 (21.1)	2354 (22.8)	0.04
Fifth (most disadvantage)	3005 (32.2)	34 327 (42.2)	0.21	458 (18.5)	4559 (18.5)	0.00	284 (27.3)	2901 (28.1)	0.02
Maternal BMI^g^									
Underweight (BMI <18.5)	218 (2.5)	1728 (2.3)	0.01	22 (1.5)	206 (1.4)	0.01	13 (1.7)	155 (2.1)	0.03
Normal (BMI 18.5 to <25)	3007 (34.7)	24 143 (32.2)	0.05	528 (34.9)	5424 (35.9)	0.02	257 (34.3)	2620 (35.4)	0.02
Overweight (BMI 25 to <30)	2502 (28.8)	22 139 (29.6)	0.02	417 (27.6)	4041 (26.7)	0.02	215 (28.7)	2108 (28.4)	0.01
Obesity (BMI ≥30)	2537 (29.2)	25 535 (34.1)	0.10	405 (26.8)	4135 (27.3)	0.01	237 (31.6)	2342 (31.6)	0.00
Parity									
Nulliparous	3754 (40.3)	27 456 (33.8)	0.13	1024 (33.8)	10 118 (33.5)	0.01	341 (32.7)	3294 (31.9)	0.02
1	4254 (45.6)	42 577 (52.4)	0.13	1209 (39.9)	12 221 (40.4)	0.01	502 (48.2)	4899 (47.5)	0.01
≥2	1317 (14.1)	11 295 (13.9)	0.01	798 (26.3)	7901 (26.1)	0.00	199 (19.1)	2125 (20.6)	0.04
A previous child having a major congenital malformation	252 (2.7)	2624 (3.2)	0.03	85 (2.8)	805 (2.7)	0.01	31 (3.0)	297 (2.9)	0.01
Maternal hospitalization during 12 mo before date of conception									
None	6921 (74.2)	59 481 (73.1)	0.02	2388 (78.8)	24 113 (79.7)	0.02	790 (75.8)	7680 (74.4)	0.03
1	1597 (17.1)	14 393 (17.7)	0.02	470 (15.5)	4425 (14.6)	0.02	174 (16.7)	1739 (16.9)	0.00
≥2	807 (8.7)	7454 (9.2)	0.02	173 (5.7)	1702 (5.6)	0.00	78 (7.5)	899 (8.7)	0.04
Maternal morbidity									
Mental health disorder	2383 (25.6)	14 311 (17.6)	0.19	737 (24.3)	7073 (23.4)	0.02	723 (69.4)	6994 (67.8)	0.03
Chronic airway disorder	1855 (19.9)	12 711 (15.6)	0.11	530 (17.5)	5067 (16.8)	0.02	267 (25.6)	2498 (24.2)	0.03
Gastroesophageal reflux	648 (6.9)	3879 (4.8)	0.09	265 (8.7)	2519 (8.3)	0.01	102 (9.8)	906 (8.8)	0.03
Use of nonsteroid anti-inflammatory drugs	2876 (30.8)	20 207 (24.8)	0.13	636 (21.0)	6134 (20.3)	0.02	322 (30.9)	3048 (29.5)	0.03
Use of steroids	927 (9.9)	6166 (7.6)	0.08	251 (8.3)	2406 (8.0)	0.01	117 (11.2)	1060 (10.3)	0.03
Anemia and coagulation disorders	452 (4.8)	3886 (4.8)	0.00	121 (4.0)	1098 (3.6)	0.02	48 (4.6)	423 (4.1)	0.02
Drug and alcohol disorders	407 (4.4)	3147 (3.9)	0.02	76 (2.5)	736 (2.4)	0.00	29 (2.8)^h^	391 (3.8)	0.06
Thyroid disorder	86 (0.9)	530 (0.7)	0.03	65 (2.1)	590 (2.0)	0.01	15 (1.4)^h^	97 (0.9)	0.05
Cardiovascular disease	214 (2.3)	1469 (1.8)	0.03	98 (3.2)	883 (2.9)	0.02	25 (2.4)^h^	226 (2.2)	0.01
Preexisting diabetes	111 (1.2)	675 (0.8)	0.04	60 (2.0)	505 (1.7)	0.02	15 (1.4)^h^	92 (0.9)	0.05
Preexisting hypertension	92 (1.0)	578 (0.7)	0.03	45 (1.5)	378 (1.3)	0.02	13 (1.2)^h^	109 (1.1)	0.02
Epilepsy	113 (1.2)^h^	667 (0.8)	0.04	25 (0.8)	235 (0.8)	0.01	22 (2.1)^h^	200 (1.9)	0.01
Chronic kidney disease	131 (1.4)^h^	1212 (1.5)	0.01	25 (0.8)^h^	182 (0.6)	0.03	12 (1.2)^h^	107 (1.0)	0.01

Abbreviations: BMI, body mass index (calculated as weight in kilograms divided by height in meters squared); diff, difference; NA, not applicable; NSW, New South Wales, Australia; NZ, New Zealand.

^a^ See eAppendices 3 to 5 in Supplement 1 for the distribution across years, education, remoteness, and additional categories (including missing/unknown) available in each country before and after propensity score matching.

^b^ Exposed infants were matched to unexposed infants (1:10) on propensity score and year of conception (NZ, NSW) or year of birth (Norway, Sweden). Unexposed infants were born to women who smoked during the first trimester but were not dispensed a prescribed smoking cessation pharmacotherapy during 90 days before conception and the first trimester.

^c^ Absolute standardized difference.

^d^ Indigenous status: Australian Aboriginal and/or Torres Strait Islander background (NZ) or Māori ethnicity (NZ); this was not available in Norway and Sweden.

^e^ Country of birth and relationship information was available in the data from NSW, Norway, and Sweden but not available in NZ data.

^f^ Socioeconomic deprivation quintile information was available in NZ and NSW data, but not available in Norway and Sweden data.

^g^ BMI information was available in NZ and Norway and Sweden data, but not available in NSW data.

^h^ For privacy, small counts are not shown. Cell values of less than 6 in NSW data were replaced with a 6, cell values of less than 5 in Norway and Sweden data were replaced with a 5, and other related cells were adjusted accordingly.

The prevalence of MCMs overall did not differ substantially between infants exposed to any NRT formulation (37.6 per 1000 live births) and unexposed infants (34.4 per 1000; aRR, 1.10; 95% CI, 0.98-1.22; Table 2). There were no marked differences between NRT-exposed and unexposed infants in malformations of the heart, limbs, genital organs, kidney and urinary tract, respiratory system, nervous system, abdominal wall, or orofacial clefts. Risk estimates for eye malformations (aRR, 2.06; 95% CI, 0.92-4.63) and ear malformations (aRR, 1.08; 95% CI, 0.14-8.60) were imprecise due to the few infants with an outcome (Table 2).

**Table 2.** Major Congenital Malformations, Overall and Subgroups, in Live-Born Infants Exposed to Nicotine Replacement Therapy, Varenicline, and Bupropion During the First Trimester and Matched Unexposed Infants^a^^,^^b^

Major congenital malformation	Exposed^c^			Unexposed^c^			Adjusted RR (95% CI)	*P* value from Poisson model	Benjamini-Hochberg–corrected *P* value	Estimates
	No.	Total No.	Prevalence (95% CI)	No.	Total No.	Prevalence (95% CI)				
**Nicotine replacement therapy (any formulation)**										
Overall	351	9325	37.6 (33.8-41.8)	2798	81 328	34.4 (33.1-35.7)	1.10 (0.98-1.22)	.09	.69	NZ and NSW^d^
Congenital heart defects	82	9325	8.8 (7.0-10.9)	711	81 328	8.7 (8.1-9.4)	0.99 (0.79-1.24)	.93	.97	NZ and NSW^d^
Limb anomalies	78	9325	8.4 (6.6-10.4)	597	81 328	7.3 (6.8-8.0)	1.17 (0.93-1.49)	.18	.75	NZ and NSW^d^
Genital organs	57	8678	6.6 (5.0-8.5)	435	74 873	5.8 (5.3-6.4)	1.13 (0.86-1.50)	.38	.94	NZ^e^
Kidney and urinary tract	42	8678	4.8 (3.5-6.5)	270	74 873	3.6 (3.2-4.1)	1.31 (0.94-1.82)	.11	.69	NZ^e^
Digestive system	33	8678	3.8 (2.6-5.3)	187	74 873	2.5 (2.2-2.9)	1.53 (1.05-2.23)	.03	.41	NZ^e^
Respiratory system	17	8678	2.0 (1.1-3.1)	134	74 873	1.8 (1.5-2.1)	1.10 (0.67-1.82)	.71	.97	NZ^e^
Orofacial clefts	16	8678	1.8 (1.1-3.0)	155	74 873	2.1 (1.8-2.4)	0.93 (0.56-1.54)	.77	.97	NZ^e^
Nervous system	10	8678	1.2 (0.6-2.1)	132	74 873	1.8 (1.5-2.1)	0.64 (0.33-1.22)	.17	.75	NZ^e^
Abdominal wall	11	8678	1.3 (0.6-2.3)	83	74 873	1.1 (0.9-1.4)	1.27 (0.67-2.41)	.46	.94	NZ^e^
Eye	7	8678	0.8 (0.3-1.7)	30	74 873	0.4 (0.3-0.6)	2.06 (0.92-4.63)	.08	.69	NZ^e^
Ear	<3	8678	(Supp)	8	74 873	0.1 (0.1-0.2)	1.08 (0.14-8.60)	.94	.97	NZ^e^
Others	31	8678	3.6 (2.4-5.1)	238	74 873	3.2 (2.8-3.6)	1.12 (0.77-1.63)	.55	.94	NZ^e^
**Varenicline**										
Overall	99	3031	32.7 (26.6-39.8)	1106	30 240	36.6 (34.5-38.8)	0.90 (0.73-1.10)	.29	.93	NZ, NSW, and Norway and Sweden^f^
Congenital heart defects	27	3031	8.9 (5.9-13.0)	294	30 240	9.7 (8.6-10.9)	0.99 (0.67-1.47)	.97	.97	NZ, NSW, and Norway and Sweden^f^
Limb anomalies	15	2473	6.1 (3.4-10.0)	183	24 660	7.4 (6.4-8.6)	0.85 (0.51-1.42)	.53	.94	NZ and NSW^d^
Genital organs	17	2473	6.9 (4.0-11.0)	147	24 660	6.0 (5.0-7.0)	1.26 (0.77-2.06)	.36	.94	NZ and NSW^d^
Kidney and urinary tract	11	954	11.5 (5.8-20.6)	40	9540	4.2 (3.0-5.7)	2.75 (1.42-5.34)	<.01	.09	NZ^e^
Digestive system	0	954	NA	23	9540	2.4 (1.4-3.5)	NA	NA	NA	NZ^e^
Respiratory system	<3	954	(Supp)	11	9540	1.2 (0.6-2.1)	0.91 (0.12-6.90)	.93	.97	NZ^e^
Orofacial clefts	<3	954	(Supp)	21	9540	2.2 (1.4-3.4)	0.47 (0.06-3.52)	.47	.94	NZ^e^
Nervous system	3	954	3.1 (0.7-9.2)	21	9540	2.2 (1.4-3.4)	1.43 (0.43-4.76)	.56	.94	NZ^e^
Abdominal wall	<3	954	(Supp)	12	9540	1.3 (0.7-2.2)	0.83 (0.11-6.31)	.86	.97	NZ^e^
Eye	0	954	NA	5	9540	0.5 (0.2-1.2)	NA	NA	NA	NZ^e^
Ear	0	954	NA	<3	9540	(Supp)	NA	NA	NA	NZ^e^
Others	9	1519	5.9 (2.7-11.3)	71	15 120	4.7 (3.7-5.9)	1.25 (0.63-2.49)	.52	.94	NSW^g^
**Bupropion**										
Overall	37	1042	35.5 (25.0-48.9)	400	10 318	38.8 (35.1-42.8)	0.93 (0.67-1.29)	.66	.96	NZ and NSW^d^
Congenital heart defects	5	749	6.7 (2.2-15.6)	86	7410	11.6 (9.3-14.3)	0.55 (0.21-1.42)	.22	.77	NZ^e^
Limb anomalies	5	749	6.7 (2.2-15.6)	58	7410	7.8 (5.9-10.1)	0.86 (0.34-2.18)	.76	.97	NZ^e^
Genital organs	7	749	9.4 (3.8-19.3)	53	7410	7.2 (5.4-9.4)	1.38 (0.66-2.90)	.39	.94	NZ^e^
Kidney and urinary tract	4	749	5.3 (1.5-13.7)	35	7410	4.7 (3.3-6.6)	1.27 (0.53-3.08)	.59	.95	NZ^e^
Digestive system	0	749	NA	22	7410	3.0 (1.9-4.5)	NA	NA	NA	NZ^e^
Respiratory system	<3	749	(Supp)	18	7410	2.4 (1.4-3.8)	1.10 (0.26-4.66)	.90	.97	NZ^e^
Orofacial clefts	0	749	NA	12	7410	1.6 (0.8-2.8)	NA	NA	NA	NZ^e^
Nervous system	0	749	NA	10	7410	1.4 (0.7-2.5)	NA	NA	NA	NZ^e^
Abdominal wall	<3	749	(Supp)	8	7410	1.1 (0.5-2.1)	1.24 (0.16-9.57)	.84	.97	NZ^e^
Eye	<3	749	(Supp)	<3	7410	(Supp)	4.96 (0.46-53.24)	.19	.75	NZ^e^
Ear	0	749	NA	0	7410	NA	NA	NA	NA	NZ^e^
Others	3	749	4.0 (0.8-11.7)	22	7410	3.0 (1.9-4.5)	1.35 (0.41-4.41)	.62	.95	NZ^e^

Abbreviations: NA, not applicable; NSW, New South Wales, Australia; NZ, New Zealand; RR, relative risk; supp, data suppression.

^a^ For privacy, data suppression was applied to small counts (<3 for NZ, <6 for NSW, and <5 for Norway and Sweden) and such cells were not included in the pooled RR.

^b^ See eTables 17 and 18 in Supplement 1 for country-specific unadjusted and adjusted results.

^c^ Exposed infants were matched to unexposed infants (1:10) on propensity score and year of conception (NZ and NSW) or year of birth (Norway and Sweden). Unexposed infants were born to women who smoked during the first trimester but were not dispensed a prescribed smoking cessation pharmacotherapy during 90 days before conception and the first trimester.

^d^ Estimates were pooled across the NZ and NSW cohorts.

^e^ Estimates were based on the NZ cohort.

^f^ Estimates were pooled across the NZ, NSW, and Norway and Sweden cohorts.

^g^ Estimates were based on the NSW cohort.

The prevalence of digestive system MCMs was higher in NRT-exposed infants than in unexposed infants (3.8 vs 2.5 per 1000 live births; aRR, 1.53; 95% CI, 1.05-2.23); however, the *P* value changed from .03 to .41 following adjustment for multiple comparisons (Table 2). As presented in Table 3, although NRT-exposed infants had higher RR for specific digestive system MCMs, the prevalence of the following malformations was very low: Hirschsprung disease (0.8 vs 0.2 per 1000; aRR, 3.69; 95% CI, 1.47-9.28; Benjamini-Hochberg–corrected *P* = .26), anorectal atresia or/and stenosis (0.4 vs 0.1; aRR, 3.24; 95% CI, 0.86-12.18), and atresia of bile ducts (0.4 vs 0.2; aRR, 1.92; 95% CI, 0.55-6.67). Disaggregation of nicotine patches (4173 exposed infants) and fast-acting formulations (2435 exposed infants; Table 4) yielded results that were similar to those for exposure to any NRT formulation.

**Table 3.** Specific Major Congenital Malformations in Live-Born Infants Exposed to Nicotine Replacement Therapy, Varenicline, and Bupropion During the First Trimester and Matched Unexposed Infants in the New Zealand Cohort^a^

Specific congenital malformations	Exposed^b^		Unexposed^b^		Adjusted relative risk (95% CI)	*P* value from Poisson model	Benjamini-Hochberg–corrected *P* value
	No.	Prevalence (95% CI)	No.	Prevalence (95% CI)			
**Nicotine replacement therapy, any formulation (8678 exposed, 74 873 unexposed)**							
Congenital heart defects							
Ventricular septal defect	22	2.5 (1.6-3.8)	214	2.9 (2.5-3.3)	0.84 (0.54-1.30)	.44	.86
Atrial septal defect	34	3.9 (2.7-5.5)	341	4.6 (4.1-5.1)	0.81 (0.57-1.16)	.26	.68
Patent ductus arteriosus in term infants	20	2.3 (1.4-3.6)	172	2.3 (2.0-2.7)	1.04 (0.66-1.65)	.86	.94
Tetralogy and pentalogy of Fallot	4	0.5 (0.1-1.2)	27	0.4 (0.2-0.5)	1.38 (0.48-3.92)	.55	.86
Mitral valve atresia/stenosis	3	0.4 (0.1-1.0)	20	0.3 (0.2-0.4)	1.36 (0.43-4.26)	.60	.89
Limb anomalies							
Clubfoot, talipes equinovarus	23	2.7 (1.7-4.0)	277	3.7 (3.3-4.2)	0.78 (0.51-1.20)	.26	.68
Polydactyly	16	1.8 (1.1-3.0)	99	1.3 (1.1-1.6)	1.40 (0.81-2.39)	.23	.68
Hip dislocation and/or dysplasia	5	0.6 (0.2-1.3)	17	0.2 (0.1-0.4)	2.48 (0.95-6.45)	.06	.58
Limb reduction defects	5	0.6 (0.2-1.3)	20	0.3 (0.2-0.4)	2.18 (0.82-5.76)	.12	.68
Genital organs							
Hypospadias	26	3.0 (2.0-4.4)	148	2.0 (1.7-2.3)	1.48 (0.96-2.28)	.08	.58
Orofacial clefts							
Cleft lip with or without cleft palate	9	1.0 (0.5-2.0)	56	0.8 (0.6-1.0)	1.49 (0.73-3.03)	.27	.68
Cleft palate	10	1.2 (0.6-2.1)	129	1.7 (1.4-2.1)	0.70 (0.37-1.32)	.27	.68
Kidney and urinary tract							
Congenital hydronephrosis	12	1.4 (0.7-2.4)	71	1.0 (0.7-1.2)	1.38 (0.74-2.57)	.30	.68
Multicystic kidney dysplasia	6	0.7 (0.3-1.5)	22	0.3 (0.2-0.4)	2.49 (0.98-6.34)	.06	.58
Digestive system							
Hirschsprung disease	7	0.8 (0.3-1.7)	15	0.2 (0.1-0.3)	3.69 (1.47-9.28)	.01	.26
Anorectal atresia and/or stenosis	3	0.4 (0.1-1.0)	8	0.1 (0.1-0.2)	3.24 (0.86-12.18)	.08	.58
Atresia of bile ducts	3	0.4 (0.1-1.0)	13	0.2 (0.1-0.3)	1.92 (0.55-6.67)	.30	.68
Nervous system							
Severe microcephaly	4	0.5 (0.1-1.2)	27	0.4 (0.2-0.5)	1.09 (0.38-3.10)	.88	.94
**Varenicline (954 exposed, 9540 unexposed)**							
Congenital heart defects							
Ventricular septal defect	3	3.1 (0.7-9.2)	36	3.8 (2.6-5.2)	0.92 (0.29-2.92)	.88	.94
Atrial septal defect	5	5.2 (1.7-12.2)	51	5.4 (4.0-7.0)	0.92 (0.38-2.24)	.86	.94
Kidney and urinary tract							
Congenital hydronephrosis	4	4.2 (1.1-10.7)	12	1.3 (0.7-2.2)	3.34 (1.08-10.29)	.04	.58
**Bupropion (749 exposed, 7410 unexposed)**							
Congenital heart defects							
Ventricular septal defect	4	5.3 (1.5-13.7)	22	3.0 (1.9-4.5)	1.79 (0.62-5.16)	.28	.68
Genital organs							
Hypospadias	3	4.0 (0.8-11.7)	27	3.6 (2.4-5.3)	1.18 (0.42-3.33)	.75	.94

^a^ See eTables 20 and 21 in Supplement 1 for unadjusted and adjusted results of specific major congenital malformations.

^b^ Exposed infants were matched to unexposed infants (1:10) on propensity score and year of conception. Unexposed infants were born to women who smoked during the first trimester but were not dispensed a prescribed smoking cessation pharmacotherapy during the 90 days before conception and the first trimester.

**Table 4.** Major Congenital Malformations, Overall and Subgroups, in Live-Born Infants Exposed to Nicotine Transdermal Patches and Fast-Acting Formulations During the First Trimester and Matched Unexposed Infants^a^^,^^b^

Major congenital malformation	Exposed^c^			Unexposed^c^			Adjusted RR (95% CI)	*P* value from Poisson model	Benjamini-Hochberg corrected–*P* value	Estimates
	No.	Total No.	Prevalence (95% CI)	No.	Total No.	Prevalence (95% CI)				
**Transdermal patches only**										
Overall	167	4173	40.0 (34.2-46.6)	1446	41 649	34.7 (33.0-36.6)	1.17 (1.00-1.37)	.05	.60	NZ and NSW^d^
Congenital heart defects	43	4173	10.3 (7.5-13.9)	385	41 649	9.2 (8.3-10.2)	1.17 (0.85-1.60)	.33	.86	NZ and NSW^d^
Limb anomalies	36	4173	8.6 (6.0-11.9)	284	41 649	6.8 (6.1-7.7)	1.31 (0.93-1.84)	.12	.76	NZ and NSW^d^
Genital organs	21	3526	6.0 (3.7-9.1)	210	35 194	6.0 (5.2-6.8)	1.01 (0.64-1.59)	.96	.97	NZ^e^
Kidney and urinary tract	19	3526	5.4 (3.2-8.4)	139	35 194	4.0 (3.3-4.7)	1.38 (0.86-2.22)	.18	.80	NZ^e^
Digestive system	16	3526	4.5 (2.6-7.4)	91	35 194	2.6 (2.1-3.2)	1.77 (1.04-2.99)	.03	.60	NZ^e^
Respiratory system	7	3526	2.0 (0.8-4.1)	57	35 194	1.6 (1.2-2.1)	1.16 (0.50-2.67)	.73	.86	NZ^e^
Orofacial clefts	7	3526	2.0 (0.8-4.1)	64	35 194	1.8 (1.4-2.3)	1.10 (0.52-2.33)	.81	.88	NZ^e^
Nervous system	4	3526	1.1 (0.3-2.9)	54	35 194	1.5 (1.2-2.0)	0.77 (0.29-2.05)	.60	.86	NZ^e^
Abdominal wall	4	3526	1.1 (0.3-2.9)	34	35 194	1.0 (0.7-1.4)	1.18 (0.42-3.31)	.76	.86	NZ^e^
Eye	<3	3526	(Supp)	18	35 194	0.5 (0.3-0.8)	0.56 (0.07-4.14)	.57	.86	NZ^e^
Ear	<3	3526	(Supp)	4	35 194	0.1 (0.0-0.3)	2.50 (0.28-22.18)	.41	.86	NZ^e^
Others	15	3526	4.3 (2.4-7.0)	109	35 194	3.1 (2.5-3.7)	1.43 (0.83-2.46)	.19	.80	NZ^e^
**Fast-acting formulations only (lozenges, gums)**										
Overall	89	2435	36.6 (29.4-45.0)	880	24 290	36.2 (33.9-38.7)	1.00 (0.81-1.24)	.97	.97	NZ^e^
Congenital heart defects	29	2435	11.9 (8.0-17.1)	247	24 290	10.2 (8.9-11.5)	1.17 (0.80-1.73)	.41	.86	NZ^e^
Limb anomalies	22	2435	9.0 (5.7-13.7)	174	24 290	7.2 (6.1-8.3)	1.27 (0.81-1.99)	.30	.86	NZ^e^
Genital organs	11	2435	4.5 (2.3-8.1)	159	24 290	6.6 (5.6-7.7)	0.73 (0.41-1.30)	.28	.86	NZ^e^
Kidney and urinary tract	7	2435	2.9 (1.2-5.9)	85	24 290	3.5 (2.8-4.3)	0.82 (0.38-1.77)	.62	.86	NZ^e^
Digestive system	12	2435	4.9 (2.6-8.6)	72	24 290	3.0 (2.3-3.7)	1.63 (0.88-3.01)	.12	.76	NZ^e^
Respiratory system	5	2435	2.1 (0.7-4.8)	41	24 290	1.7 (1.2-2.3)	1.22 (0.48-3.07)	.68	.86	NZ^e^
Orofacial clefts	5	2435	2.1 (0.7-4.8)	43	24 290	1.8 (1.3-2.4)	1.16 (0.46-2.90)	.75	.86	NZ^e^
Nervous system	3	2435	1.2 (0.3-3.6)	51	24 290	2.1 (1.6-2.8)	0.59 (0.18-1.89)	.37	.86	NZ^e^
Abdominal wall	<3	2435	(Supp)	27	24 290	1.1 (0.7-1.6)	0.74 (0.18-3.09)	.68	.86	NZ^e^
Eye	<3	2435	(Supp)	12	24 290	0.5 (0.3-0.9)	0.83 (0.11-6.31)	.68	.86	NZ^e^
Ear	0	2435	NA	3	24 290	0.1 (0.0-0.4)	NA	NA	NA	NZ^e^
Others	7	2435	2.9 (1.2-5.9)	86	24 290	3.5 (2.8-4.4)	0.83 (0.38-1.79)	.64	.86	NZ^e^

Abbreviations: NA, not applicable; NSW, New South Wales, Australia; NZ, New Zealand; supp, data suppression.

^a^ For privacy, data suppression was applied to small counts (<3 for NZ and <6 for NSW).

^b^ See eTable 19 in Supplement 1 for unadjusted results.

^c^ Exposed infants were matched to unexposed infants (1:10) on propensity score and year of conception. Unexposed infants were born to women who smoked during the first trimester but were not dispensed a prescribed smoking cessation pharmacotherapy during 90 days before conception and the first trimester.

^d^ Estimates were pooled across the NZ and NSW cohorts.

^e^ Estimates were based on the NZ cohort.

Analyses regarding varenicline showed no marked differences in the risk of MCMs overall (32.7 vs 36.6 per 1000 live births; aRR, 0.90; 95% CI, 0.73-1.10), malformation of the heart (8.9 vs 9.7; aRR, 0.99; 95% CI, 0.67-1.47), limbs (6.1 vs 7.4; aRR, 0.85; 95% CI, 0.51-1.42), or genital organs (6.9 vs 6.0; aRR, 1.26; 95% CI, 0.77-2.06). Varenicline-exposed infants had higher prevalence of kidney and urinary tract MCMs (11.5 vs 4.2 per 1000; aRR, 2.75; 95% CI, 1.42-5.34), but this was based on small numbers of affected infants (11 exposed and 40 unexposed), and the *P* value changed from <.01 to .09 following Benjamini-Hochberg correction. More specifically, varenicline-exposed infants had higher prevalence of congenital hydronephrosis (4.2 vs 1.3 per 1000; aRR, 3.34; 95% CI, 1.08-10.29; Benjamini-Hochberg–corrected *P* = .58; Table 3).

Analyses regarding bupropion showed no difference in the prevalence of MCMs overall (35.5 vs 38.8 per 1000 live births; aRR, 0.93; 95% CI, 0.67-1.29). Bupropion-exposed infants had lower prevalence of cardiac MCMs (6.7 vs 11.6 per 1000 live births; aRR, 0.55; 95% CI, 0.21-1.42), while there were no differences in MCM of the limbs (6.7 vs 7.8 per 1000 live births; aRR, 0.86; 95% CI, 0.34-2.18), genital organs (9.4 vs 7.2 per 1000 live births; aRR, 1.38; 95% CI, 0.66-2.90), or kidney and urinary tract (5.3 vs 4.7 per 1000 live births; aRR, 1.27; 95% CI, 0.53-3.08), noting that these were based on small numbers of exposed infants. Data for other subgroups malformations were too sparse.

The sensitivity analyses that restricted the exposed groups to infants of women recorded as smoking (5545 receiving NRT, 1095 varenicline, and 522 bupropion) yielded results that were consistent with the main analyses. The sensitivity analyses that restricted the exposed groups to infants of women who received 2 or more dispensings (3096 of NRT, 249 of varenicline, and 128 of bupropion) also yielded consistent results, with 2 exceptions: the aRR of NRT-associated eye MCMs was 3.33 (95% CI, 1.10-10.02; based on 4 exposed and 12 unexposed infants), and the aRR of varenicline-associated kidney and urinary tract MCMs was 7.19 (95% CI, 2.38-21.79; based on 5 exposed and 7 unexposed infants; eTable 22 in Supplement 1). The analyses quantifying potential bias due to inclusion of only live births revealed that under the most extreme scenarios modeled (ie, among pregnancies with an MCM, the probability of miscarriage or termination was ≥50% in the unexposed and ≥70% in the exposed group), the observed aRR for MCMs overall would shift from 1.10 to 1.38 for NRT, 0.90 to 1.13 for varenicline, and 0.93 to 1.16 for bupropion (eFigures 1-3 in Supplement 1). The *E* value (eTable 23 in Supplement 1) for the association between NRT and digestive system MCMs was 2.43, ie, residual confounding from an unmeasured factor could fully explain the observed association if the unmeasured factor was associated with NRT exposure and digestive system MCMs with an RR of at least 2.43. The *E* value for the association between NRT and eye MCMs was 3.54, and between varenicline and kidney/urinary tract MCMs it was 4.94.

## Discussion

To our knowledge, this cohort study was the largest to date that investigated the risk of MCMs following prenatal exposure to smoking cessation pharmacotherapies. Our results suggested no increased risks of MCMs overall following exposure to NRT, varenicline, or bupropion compared with smoking during the first trimester. Our study also found no evidence of higher risk of several malformation subgroups among NRT-exposed and varenicline-exposed infants. Our data suggested no difference in the teratogenic risks of NRT patches and fast-acting formulations.

The null findings for NRT were reassuring, as NRT is offered to pregnant people when behavioral therapies fail.^2,6^ Our previous work using the same source data showed that up to 7% of women who smoked used an NRT product (eg, patches, lozenges, or gums) during the first trimester of pregnancy.^7^ Our study findings did not support previous reports that NRT was associated with an increased risk of musculoskeletal^4^ and respiratory malformations^5^; these findings may be attributable to maternal smoking.^4,5^ However, we observed an elevated risk of digestive system malformations, partly owing to a higher prevalence of Hirschsprung disease, anorectal anomalies, and bile duct anomalies among NRT-exposed infants. Compared with infants exposed to maternal smoking but not to smoking cessation pharmacotherapies, the difference in prevalence for these outcomes was small (1.3 per 1000 births for any digestive malformation, 0.6 per 1000 births for Hirschsprung disease, 0.3 per 1000 births for anorectal atresia and/or stenosis, and 0.2 per 1000 births for bile duct stenosis). Nicotine acts on the nervous system and induces vascular dysfunction, such as increased vasoconstriction and endothelial injury, potentially causing abnormal fetal morphology.^28^ However, this mechanism did not seem to be directly associated with the etiology of Hirschsprung disease nor anorectal anomalies, which are multifactorial and potentially involve genetic variants.^29,30^ Hirschsprung disease is frequently associated with syndromes, such as Down syndrome, Waardenburg-Shah syndrome, or Goldberg-Shprintzen,^29^ and anorectal anomalies occur commonly in multianomaly sequences, such as VACTERL or CHARGE associations.^30^ The association between NRT and digestive system MCMs was no longer statistically significant after adjustment for multiple comparisons, suggesting the possibility of a chance finding.

Our finding regarding the association of varenicline with MCMs overall was consistent with previous studies.^11,12^ To our knowledge, our study was the first to provide evidence on the risk of cardiac, limb, and genital organ malformations for varenicline, and we found no increased risk. However, we observed a higher prevalence of kidney and urinary tract MCMs among varenicline-exposed infants (11.5 per 1000 live births) compared with unexposed infants (4.2 per 1000 live births), and specifically a higher prevalence of congenital hydronephrosis (4.2 vs 1.3 per 1000 live births). To date, to our knowledge, no mechanism has been identified by which varenicline could disrupt normal fetal organogenesis. Congenital malformations of kidney and urinary tract can be isolated or associated with environmental and genetic factors.^31^ Our finding regarding the increased risk of kidney and urinary tract malformations associated with varenicline should be interpreted cautiously because it was based on only 11 exposed and 40 unexposed infants, and the association was no longer statistically significant after adjustment for multiple comparisons. More data on the potential teratogenicity of varenicline are needed given that it is the most effective pharmacotherapy for smoking cessation.^2,3^

Data on the safety of bupropion are emerging but remain sparse.^13,14^ While 3 studies found no evidence of increased risks of congenital malformations, another 3 studies reported an increased risk of cardiac defects.^13,14^ However, evidence from these prior studies is subject to selection bias and confounding by indication.^13,32^ A large-scale study of bupropion as an antidepressant highlighted that the association between heart defects and bupropion and other antidepressants may be confounded by the underlying condition. After controlling for depression, bupropion was only associated with diaphragmatic hernia.^32^ To our knowledge, our study was the first rigorous investigation of teratogenic effects of bupropion for smoking cessation. The use of bupropion during pregnancy for smoking cessation was very low (<0.4% among women who smoked),^7^ limiting our ability to robustly estimate the risk of MCM subgroups. Nevertheless, our study suggests no evidence that bupropion was associated with an increased risk of MCMs overall.

### Strengths and Limitations

The main strengths of this study were the use of clinical, population-wide data with rich person-level information, which prevented recall bias and allowed us to control for key covariates. Nevertheless, there were several limitations. This study did not examine long-term functional defects, such as adverse neurodevelopmental outcomes. Furthermore, we assumed that all women in the study smoked at some point during the first trimester, and sensitivity analyses among those who self-reported that they smoked during the first trimester confirmed the robustness of the main findings. However, we lacked data on whether women smoked throughout the entire trimester. Additionally, some infants of women without a pharmacotherapy dispensing might have been exposed to OTC NRT. Nonetheless, we expect such misclassification to be minimal and unlikely to bias the results because many of our pooled estimates were largely based on data from NZ, where OTC NRT use is limited.^7^ Moreover, women in the exposed groups might have discontinued therapy when they realized that they were pregnant. However, pregnancy recognition typically occurs around week 6 of gestation,^33^ by which time exposure to these medicines could already have affected the development of key body systems.^34^ The analyses that were restricted to women who received 2 or more dispensings (indicating a higher likelihood of actual pharmacotherapy use) further confirmed the overall pattern of null findings. Finally, stillborn infants were excluded due to concerns about the poor quality of malformation recording among stillbirths,^35,36^ and because stillbirths were rare in our study. Similarly, we lacked data on pregnancy terminations. However, quantitative bias analyses indicated that restricting the analyses to live births is unlikely to have influenced our findings.

## Conclusions

The results of this cohort study suggest that neither NRT, varenicline, nor bupropion use during the first trimester was associated with an increased risk of MCMs overall compared with smoking. We also found no increased risk of malformations of several body systems following varenicline and NRT exposure, including NRT patches specifically. Although we observed an elevated risk of digestive organ MCMs for NRT and kidney and urinary tract MCMs for varenicline, the numbers of affected infants were small, and these findings were likely due to chance. Overall, our findings are reassuring given the extensive detrimental effects of smoking on maternal and child health. Larger studies providing more robust estimates of risk for the remaining malformation subgroups are needed.

## Notes

Supplement 1. eAppendix 1. Description of data sources and cohort inclusion period eAppendix 2. Definitions and contextual information eAppendix 3. Characteristics of infants in New Zealand cohort, before and after propensity score matching eAppendix 4. Characteristics of infants in New South Wales cohort, Australia, before and after propensity score matching eAppendix 5. Characteristics of infants in Norway and Sweden cohort, before and after propensity score matching eAppendix 6. Major congenital malformations, overall and subgroups: unadjusted and adjusted results eAppendix 7. Specific major congenital malformations, based on New Zealand cohort eAppendix 8. Sensitivity analyses eTable 1. Description of population data collection used in this study eTable 2. Availability, recommended dosing and days of supply calculation eTable 3. Definition of major congenital malformation categories (subgroups) eTable 4. Definition of specific major congenital malformations eTable 5. Known and potentially teratogenic medicines eTable 6. Definitions of infant and maternal socio-demographic characteristics and obstetric variables eTable 7. Definitions of pre-existing maternal morbidities eTable 8. Characteristics of infants exposed to nicotine replacement therapy (NRT any formulation) and unexposed infants in New Zealand cohort, before and after propensity score matching eTable 9. Characteristics of infants exposed to varenicline and unexposed infants in New Zealand cohort, before and after propensity score matching eTable 10. Characteristics of infants exposed to bupropion and unexposed infants in New Zealand cohort, before and after propensity score matching eTable 11. Characteristics of infants exposed to transdermal nicotine patches only and unexposed infants in New Zealand cohort, before and after propensity score matching eTable 12. Characteristics of infants exposed to fast-acting nicotine formulations (lozenges, gums) only and unexposed infants in New Zealand cohort, before and after propensity score matching 22 eTable 13. Characteristics of infants exposed to transdermal nicotine patches and unexposed infants in New South Wales cohort, Australia, before and after propensity score matching eTable 14. Characteristics of infants exposed to varenicline and unexposed infants in New South Wales cohort, Australia, before and after propensity score matching eTable 15. Characteristics of infants exposed to bupropion and unexposed infants in New South Wales cohort, Australia, before and after propensity score matching eTable 16. Characteristics of infants exposed to varenicline and unexposed infants in Norway and Sweden cohort, before and after propensity score matching eTable 17. Major congenital malformations (overall and subgroups) in liveborn infants exposed to nicotine replacement therapy, varenicline, or bupropion in the first trimester and unexposed infants, before propensity score matching: country-specific number, prevalence and unadjusted relative risk eTable 18. Major congenital malformations (overall and subgroups) in liveborn infants exposed to nicotine replacement therapy, varenicline, or bupropion in the first trimester and unexposed infants, after propensity score matching: country-specific number, prevalence and adjusted relative risk, and pooled relative risk eTable 19. Major congenital malformations (overall and subgroups) in liveborn infants exposed to nicotine transdermal patches and fast-acting formulations in the first trimester and unexposed infants, before propensity score matching: country-specific number, prevalence and unadjusted relative risk eTable 20. Specific major congenital malformations in liveborn infants exposed to nicotine replacement therapy, varenicline, bupropion in the first trimester and unexposed infants, based on New Zealand cohort before propensity score matching: number, prevalence and unadjusted relative risk eTable 21. Specific major congenital malformations in liveborn infants exposed to nicotine replacement therapy, varenicline, bupropion in the first trimester and unexposed infants, based on New Zealand cohort after propensity score matching: number, prevalence and adjusted relative risk eTable 22. Sensitivity analyses: number, prevalence and adjusted relative risk eTable 23. E-values for the risk of MCM subgroups with potential signals following exposure to NRT and varenicline eFigure 1. Plausible values for corrected relative risks for the effects of exposure to nicotine replacement therapy (any formulation) in the first trimester and major congenital malformations eFigure 2. Plausible values for corrected relative risks (RR) for the effects of exposure to varenicline in the first trimester and major congenital malformations (MCMs) eFigure 3. Plausible values for corrected relative risks (RR) for the effects of exposure to bupropion in the first trimester and major congenital malformations (MCMs) overall eReferences.

Supplement 2. Data sharing statement
