# Supplementary material for: Risk of Major Congenital Malformations Following Prenatal Exposure to Smoking Cessation Medicines
Source: JAMA Intern Med. 2025 Mar 31;185(6):656–67. doi: 10.1001/jamainternmed.2025.0290 (PMC11959474; doi:10.1001/jamainternmed.2025.0290)
Supplement: Supplement 1. — eAppendix 1. Description of data sources and cohort inclusion period eAppendix 2. Definitions and contextual information eAppendix 3. Characteristics of infants in New Zealand cohort, before and after propensity score matching eAppendix 4. Characteristics of infants in New South Wales cohort, Australia, before and after propensity score matching eAppendix 5. Characteristics of infants in Norway and Sweden cohort, before and after propensity score matching eAppendix 6. Major congenital malformations, overall and subgroups: unadjusted and adjusted results eAppendix 7. Specific major congenital malformations, based on New Zealand cohort eAppendix 8. Sensitivity analyses eTable 1. Description of population data collection used in this study eTable 2. Availability, recommended dosing and days of supply calculation eTable 3. Definition of major congenital malformation categories (subgroups) eTable 4. Definition of specific major congenital malformations eTable 5. Known and potentially teratogenic medicines eTable 6. Definitions of infant and maternal socio-demographic characteristics and obstetric variables eTable 7. Definitions of pre-existing maternal morbidities eTable 8. Characteristics of infants exposed to nicotine replacement therapy (NRT any formulation) and unexposed infants in New Zealand cohort, before and after propensity score matching eTable 9. Characteristics of infants exposed to varenicline and unexposed infants in New Zealand cohort, before and after propensity score matching eTable 10. Characteristics of infants exposed to bupropion and unexposed infants in New Zealand cohort, before and after propensity score matching eTable 11. Characteristics of infants exposed to transdermal nicotine patches only and unexposed infants in New Zealand cohort, before and after propensity score matching eTable 12. Characteristics of infants exposed to fast-acting nicotine formulations (lozenges, gums) only and unexposed infants in New Zealand cohort, before and after pr [file jamainternmed-e250290-s001.pdf]

Supplemental Online Content

Tran DT, Cohen JM, Donald S, et al. Risk of major congenital malformations following prenatal exposure to smoking cessation medicines. *JAMA Intern Med*. Published online March 31, 2025. doi: 10.1001/jamainternmed.2025.0290

eAppendix 1. Description of data sources and cohort inclusion period
eAppendix 2. Definitions and contextual information
eAppendix 3. Characteristics of infants in New Zealand cohort, before and after propensity score matching
eAppendix 4. Characteristics of infants in New South Wales cohort, Australia, before and after propensity score matching
eAppendix 5. Characteristics of infants in Norway and Sweden cohort, before and after propensity score matching
eAppendix 6. Major congenital malformations, overall and subgroups: unadjusted and adjusted results
eAppendix 7. Specific major congenital malformations, based on New Zealand cohort
eAppendix 8. Sensitivity analyses
eTable 1. Description of population data collection used in this study
eTable 2. Availability, recommended dosing and days of supply calculation
eTable 3. Definition of major congenital malformation categories (subgroups)
eTable 4. Definition of specific major congenital malformations
eTable 5. Known and potentially teratogenic medicines
eTable 6. Definitions of infant and maternal socio-demographic characteristics and obstetric variables
eTable 7. Definitions of pre-existing maternal morbidities
eTable 8. Characteristics of infants exposed to nicotine replacement therapy (NRT any formulation) and unexposed infants in New Zealand cohort, before and after propensity score matching
eTable 9. Characteristics of infants exposed to varenicline and unexposed infants in New Zealand cohort, before and after propensity score matching
eTable 10. Characteristics of infants exposed to bupropion and unexposed infants in New Zealand cohort, before and after propensity score matching
eTable 11. Characteristics of infants exposed to transdermal nicotine patches only and unexposed infants in New Zealand cohort, before and after propensity score matching
eTable 12. Characteristics of infants exposed to fast-acting nicotine formulations (lozenges, gums) only and unexposed infants in New Zealand cohort, before and after propensity score matching 22
eTable 13. Characteristics of infants exposed to transdermal nicotine patches and unexposed infants in New South Wales cohort, Australia, before and after propensity score matching
eTable 14. Characteristics of infants exposed to varenicline and unexposed infants in New South Wales cohort, Australia, before and after propensity score matching

© 2025 Tran DT et al. *JAMA Internal Medicine*.

eTable 15. Characteristics of infants exposed to bupropion and unexposed infants in New South Wales cohort, Australia, before and after propensity score matching
eTable 16. Characteristics of infants exposed to varenicline and unexposed infants in Norway and Sweden cohort, before and after propensity score matching
eTable 17. Major congenital malformations (overall and subgroups) in liveborn infants exposed to nicotine replacement therapy, varenicline, or bupropion in the first trimester and unexposed infants, before propensity score matching: country-specific number, prevalence and unadjusted relative risk
eTable 18. Major congenital malformations (overall and subgroups) in liveborn infants exposed to nicotine replacement therapy, varenicline, or bupropion in the first trimester and unexposed infants, after propensity score matching: country-specific number, prevalence and adjusted relative risk, and pooled relative risk
eTable 19. Major congenital malformations (overall and subgroups) in liveborn infants exposed to nicotine transdermal patches and fast-acting formulations in the first trimester and unexposed infants, before propensity score matching: country-specific number, prevalence and unadjusted relative risk
eTable 20. Specific major congenital malformations in liveborn infants exposed to nicotine replacement therapy, varenicline, bupropion in the first trimester and unexposed infants, based on New Zealand cohort before propensity score matching: number, prevalence and unadjusted relative risk
eTable 21. Specific major congenital malformations in liveborn infants exposed to nicotine replacement therapy, varenicline, bupropion in the first trimester and unexposed infants, based on New Zealand cohort after propensity score matching: number, prevalence and adjusted relative risk
eTable 22. Sensitivity analyses: number, prevalence and adjusted relative risk
eTable 23. E-values for the risk of MCM subgroups with potential signals following exposure to NRT and varenicline
eFigure 1. Plausible values for corrected relative risks for the effects of exposure to nicotine replacement therapy (any formulation) in the first trimester and major congenital malformations
eFigure 2. Plausible values for corrected relative risks (RR) for the effects of exposure to varenicline in the first trimester and major congenital malformations (MCMs)
eFigure 3. Plausible values for corrected relative risks (RR) for the effects of exposure to bupropion in the first trimester and major congenital malformations (MCMs) overall
eReferences.

This supplemental material has been provided by the authors to give readers additional information about their work.

© 2025 Tran DT et al. *JAMA Internal Medicine*.

1. eAppendix 1: Description of data sources and cohort inclusion period

Description of data sources

We conducted a cohort study using linked administrative data from New Zealand (NZ), Australia (New South Wales only), Norway and Sweden. New South Wales (NSW) is the most populous state in Australia. To adhere to privacy laws, we analysed unit record data within each country using a common protocol and shared summarized data. The Nordic Pregnancy Drug Safety Studies (NorPreSS) collaboration enabled us to analyse data from Norway and Sweden as a combined cohort. In each country, whole-of-population data relating to pregnancies resulting in birth were linked to records of prescription medicine dispensings, inpatient and/or outpatient care, and mortality. Specific data sources and the years for which data were available for this study are described below.

eTable 1: Description of population data collection used in this study.

Country	Data collection	Data available for this study	Description	Further resources
**New Zealand**	National Maternity Collection	January 2005 - December 2020	Records of all livebirths and stillbirths of at least 20 weeks gestation	See Donald et al (2018)[1]
	Pharmaceutical Collection	January 2005- December 2021	Records of publicly funded pharmaceutical products dispensed in community pharmacies	
	National Minimum Dataset	January 1988 – December 2021	Records of all discharges from public hospitals and publicly funded events in private hospitals	
	Mortality Collection	January 2005 to December 2020	Causes of death for all deaths registered in New Zealand, and all registered stillbirths	
	National Health Index		Demographic information for all people with a National Health Index number.	
	Programme for the Integration of Mental Health Data	July 2008 – December 2021	The national mental health and addiction information collection of service activity and outcomes data for health consumers.	
**Australia, New South Wales**	Perinatal Data Collection	July 2001 to December 2019	Records of all livebirths and stillbirths of at least 20 weeks gestation or at least 400g birth weight in NSW.	See Tran et al (2024)[2]
	Pharmaceutical Benefits Scheme	January 2002 to December 2022	Records of every publicly funded pharmaceutical product dispensed in pharmacies or private hospitals	
	Admitted Patient Data Collection	July 2001 to June 2020	Records of every public and private hospital discharges in NSW	
	Cause of Death Unit Record File	July 2001 to December 2020	Causes of death for deaths registered in NSW	
**Norway**	Medical Birth Registry of Norway	January 2004 to December 2020	Records of all livebirths and stillbirths of at least 12 weeks gestation	See Cohen et al (2021),[3]<br><br>Furu et al (2010)[4]
	Norwegian Prescription Database	January 2004 to December 2020	Records of all prescribed pharmaceuticals dispensed in pharmacies to individuals	
	Norwegian Patient Registry	January 2008 to December 2020	Records of all hospital admissions, outpatient consultations, and specialist consultations	
	Norwegian Cause of Death Registry	January 2004 to December 2020	Registration of the date of death, and the main and contributing causes of death	
**Sweden**	Swedish Medical Birth Register	January 2005 to December 2019	Records of all livebirths and stillbirths of at least 22 weeks gestation	See Cnattingius et al. (2023)[5],<br><br>Ludvigson et al. (2011)[6]
	Swedish Prescribed Drug Register	January 2005 to December 2019	Records of all prescription pharmaceutical dispensings	
	Swedish National Patient Register	January 2005 to December 2019	Records of all hospital admissions, outpatient consultations, and specialist consultations	
	Swedish Cause of Death Register	January 2005 to December 2019	Causes of deaths for all deaths registered in Sweden	
	Longitudinal Integrated Database	January 2005 to December 2019	Health insurance and labour market studies from Statistics Sweden which contains data on highest achieved education	

New Zealand

- The National Maternity Collection is a national collection of pregnancies that resulted in deliveries of livebirths and stillbirths of at least 20 weeks of gestation, including home births and hospital births. This data collection contains information about pre-pregnancy maternal health, neonatal outcomes, complications during pregnancy, birth and postnatal period for mothers and babies.
- The Pharmaceutical Collection contains records of publicly funded pharmaceutical products dispensed in community pharmacies. Individual medicines have a unique chemical ID number and are organised according to therapeutic groups. In general, Level 1 therapeutic groups are organised by body system (e.g., nervous system), with Level 2 therapeutic groups organised by functional group (e.g., antidepressants) and Level 3 by drug class (e.g., selective serotonin reuptake inhibitors). Medicines purchased over the counter and those administered in public hospitals are not included.
- The National Minimum Dataset is a national collection of public and private hospital discharge information, including coded clinical data for inpatients and day patients. Information includes patient demographics, diagnoses, procedures, and external causes of injuries. Diagnoses are coded according to the International Statistical Classification of Diseases and Related Health Problems, 10th Revision-Australian Modification (ICD-10-AM) while procedures are coded according to the Australian Classification of Health Interventions (ACHI).
- The Mortality Collection classifies the underlying cause of death for all deaths registered in NZ, and all registerable stillbirths, using ICD-10-AM classification and the World Health Organisation International Statistical Classification Rules and Guidelines for Mortality Coding.
- The National Health Index includes demographic data such as ethnicity and an area-based measure of deprivation.
- The Programme for the Integration of Mental Health Data is the national mental health and addiction information collection of service activity and outcomes data for health consumers.

Further details about the New Zealand pregnancy cohort are reported elsewhere.[1]

New South Wales, Australia

- The NSW Perinatal Data Collection includes records for all livebirths and stillbirths of at least 20 weeks gestation or at least 400g birth weight delivered in NSW. Information on maternal characteristics, complications during pregnancy and delivery, and neonatal outcomes are recorded by the attending midwife or doctor.
- The Pharmaceutical Benefits Scheme (PBS) data include a record for every PBS listed medicines dispensed to eligible individuals in community pharmacies and private hospitals. Information includes PBS item number, generic name, anatomical therapeutic chemical (ATC) code, date of prescribing, date of supply, quantity, and patient beneficiary status (social security or general). Medicines purchased over the counter and those administered in public hospitals are not included.
- The Admitted Patient Data Collection includes a record of every public and private hospital discharges in NSW. Information includes patient demographics, diagnoses, procedures, and external causes of injuries. Diagnoses are coded according to the ICD-10-AM while procedures are coded according to the ACHI.
- The Cause of Death Unit Record File provides information about causes of death and coded according to the International Statistical Classification of Diseases and Related Problems, 10th Revision (ICD-10).

More details regarding the NSW linked data are reported elsewhere.[2]

Norway

- The Medical Birth Registry of Norway is a mandatory registration of all livebirths and stillbirths and includes pregnancies of at least 12 weeks of gestation. The register contains information about pre-pregnancy maternal health, complications during pregnancy or birth, neonatal outcomes, infant diagnoses, or evidence of congenital abnormalities. It also contains data about miscarriages and pregnancy termination for fetal anomaly.[7]
- The Norwegian Prescription Database is a mandatory registration of all medicines dispensed in pharmacies in Norway. Information includes ATC codes, medicine strength, defined daily doses in a package, package size and dispensing date. Medicines that are purchased over the counter or supplied to hospitals and nursing homes

are not included. Since March 2009, indication for reimbursement was coded according to ICD-10 codes and version 2 of the International Classification of Primary Care.[4]
- The Norwegian Patient Registry is a nationwide registry covering all hospital admissions, outpatient consultations, and specialist consultations. Diagnoses (primary and several secondary diagnoses) have been coded according to ICD-10 since 2008.[8]
- The Norwegian Cause of Death Registry includes registration of the date of death, and the main and contributing causes of death using ICD-10 codes since 1996.[8]

Sweden
- The Swedish Medical Birth Register covers all livebirths and stillbirths of at least 22 weeks gestation (gestation ≥28 weeks before 2008). Information includes pre-pregnancy maternal health, complications during pregnancy or birth, neonatal outcomes, and infant diagnoses.[5]
- The Swedish Prescribed Drug Register contains complete national data on all prescription pharmaceuticals dispensed. Dispensings records contain ATC codes, date of dispensing, the dose, and quantity. Hospital administered medications, vaccines and over the counter medications are not included.[4]
- The Swedish National Patient Register covers all hospital admissions, outpatient consultations, and specialist consultations. Diagnoses are coded according to ICD-10 and surgical procedures coded according to Nordic Classification of Surgical Procedures.[6]
- The Swedish Cause of Death Register comprises data on all deaths of people registered in Sweden.[9]
- Longitudinal Integrated Database for health insurance and labour market studies from Statistics Sweden which contains data on highest achieved education each year. [10]

Further information about the data sources of the Nordic Pregnancy Drug Safety Studies collaboration is reported elsewhere.[3,4]

Cohort inclusion period
The cohort inclusion period varies according to data availability. The base cohort included births with an estimated date of conception as below:
- New Zealand: conceptions from 1 January 2006 to 30 September 2019 (childbirths 2006-2020),
- New South Wales: conceptions from 1 July 2002 to 31 March 2018 (childbirths 2002-2018),
- Norway: conceptions from 1 January 2009 to 31 March 2019 (childbirths 2009-2019), and
- Sweden: conceptions from 1 July 2006 to 31 March 2018 (childbirths 2007-2018).

© 2025 Tran DT et al. *JAMA Internal Medicine*.

2. eAppendix 2: Definitions and contextual information

2.1. Definition of date of conception

Date of conception (DoC) was estimated using one of the following methods:[1,11]

- the last menstrual period (LMP) + 14 days, where LMP was available or could be inferred;
- date of childbirth – 7 * weeks of gestation + 14 days, where LMP was not available;
- expected date of delivery – 7 * weeks of gestation + 14 days, where both LMP and date of childbirth were not available.

2.2. Definition of smoking in the first trimester of pregnancy

In NZ, the National Maternity Collection contains items regarding smoking status and quantity of cigarettes smoked at the first antenatal visit (around week 10 of pregnancy). In NSW, from 2011 the Perinatal Data Collection contains items regarding whether the woman smoked in the first 20 weeks of pregnancy and quantity of cigarettes smoked in this period. Before 2011, NSW data only included an item asking whether a woman smoked at all during pregnancy, hence we'll assume that if a woman smoked during pregnancy she also smoked in the first 20 weeks. In Norway and Sweden, the Medical Birth Registry contains items regarding whether the woman smoked in the first trimester and quantity of cigarettes smoked in this period. We collectively referred smoking status in the above-described period as smoking in the first trimester.

We acknowledge, based on prior work[12] and exploratory analyses, that there may be inconsistencies in some settings between recorded smoking information[12]; for example, smoking status was recorded as "non-smoking" while the number of cigarettes smoked per day was recorded as greater than zero. We classified maternal smoking status in the first trimester as below:

- "Yes, smoked in the first trimester" when there was an affirmative response to a question asking whether a woman smoked in the first trimester or when the quantity of cigarettes smoked was greater than zero.
- "No, did not smoke in the first trimester" when there was a negative response to a question asking whether a woman smoked in the first trimester and quantity of cigarettes smoked was zero or unknown/missing; or when response to a question asking whether a woman smoked in in the first trimester was unknown/missing and quantity of cigarettes smoked was zero.
- "Smoking status unknown" when a response to a question asking whether a woman smoked in the first trimester were unknown/missing and quantity of cigarettes smoked were also unknown/missing.

In a study by Robijn et. al.[13] using the same data sources, we found that nearly a third of women who used a smoking cessation pharmacotherapy during pregnancy (2642 out of 9166) did not report that they smoked in the first trimester. For these cases, we applied a published algorithm[12] to separate potentially successful quit attempts from those that were potentially unsuccessful, using the timing and the amount of smoking cessation pharmacotherapy dispensed. Based on the algorithm, we re-classified 85% of these cases (2238/2642) as "smoked in pregnancy", hence "smoked in the first trimester". Overall, Robijn et. al.[13] indicated that 96% of women who used a smoking cessation pharmacotherapy in pregnancy smoked in the first trimester (either self-reported and documented 6524/9166 or re-classified 2238/9166).

2.3. Contextual information regarding smoking cessation pharmacotherapies

Government subsidy schemes for smoking cessation therapies vary between countries.
In New Zealand, nicotine replacement therapies (NRT, patches, lozenges, and gums) were subsidised from at least 2004, varenicline was subsidised from 2010 and bupropion was subsidised from 2009. Although NRT products can be purchased over the counter, discussions with NZ tobacco control experts, pharmacists and Pharmac (the New Zealand drug buying agency) suggest that most of the NRT obtained by individuals is likely to be via prescription and other sources captured by Pharmaceutical Collection data (author LP personal communication).

In Australia, NRT products can be purchased over the counter or via prescription. Subsidy for NRT transdermal patches commenced in 2008 for Aboriginal and Torres Strait Islander people, and the subsidy was extended to the general population in 2011.[14] Fast-acting formulations of NRT were subsidised from late 2019,[14] after the inclusion period for

© 2025 Tran DT et al. *JAMA Internal Medicine*.

NSW cohort. Varenicline (subsidised since 2008) and bupropion (subsidised since 2001) are prescription medicines only.
In Norway, NRT products are available over the counter (since 2005), but it is also possible to obtain NRT via prescription. Varenicline is available through prescription only (subsidised since 2007). Bupropion when sold under the brand name Zyban™ is indicated for smoking cessation only.

In Sweden, NRT patches and lozenges are available mostly over the counter (since 2006). Varenicline is available through prescription only (subsidised since 2007). Bupropion when sold under the brand name Zyban™ is indicated for smoking cessation only.

eTable 2 shows the availability, recommended dosing, recommended course duration, and method for calculating days of supply. In Australia, Norway and Sweden, medicines are coded according to the ATC classification system while in New Zealand, medicines are coded using the chemical ID (ChemID).

eTable 2: Availability, recommended dosing and days of supply calculation

Smoking cessation pharmacotherapies and code	Form	Strength	Pack size	Defined Daily Dose (DDD) and recommended course duration	Quantity (QTY)	Days of supply
**Nicotine Replacement Therapy (NRT)**						
**ATC N07BA01**<br>or<br>**NZ ChemID 3722**	Patch	7mg,<br>14mg,<br>21mg, 25mg*	1 or 7 or 28	One patch per day<br><br>Course of 8 to 12 weeks (56-84 days)	QTY = pack size * number dispensed	QTY/1
	Lozenge	1 mg,<br>2mg,<br>4mg# per piece	36 or 96 or 216 or 384	As needed, maximum of 30mg per day<br><br>Course of 8 to 12 weeks (56-84 days)	QTY=strength * pack size * number dispensed	QTY/30
	Gum	1mg,¥<br>2mg,<br>4mg per piece	96 or 216 or 384			
	Spray§	13.6mg per ml	13.2ml			
	Inhaler§	15mg	20	As needed, maximum of 60mg per day<br>Course of 8 to 12 weeks (56-84 days)		QTY/60
**Varenicline**						
**ATC N07BA03**<br>or<br>**NZ ChemID 3920**	Tablet	0.5mg and 1mg	25	0.5mg once a day for days 1-3, 2x 0.5mg per day for days 4-7, 2x 1mg per day for remainder of treatment.<br><br>Course of 12 weeks (84 days)	QTY = pack size * number dispensed	14
	Tablet	0.5mg and 1mg	53			28
	Tablet	1mg	56 or 112			QTY/2
	Tablet	0.5mg and 1mg	165			84
	Tablet	0.5mg and 1mg	53			28
	Tablet	1mg	56			QTY/2
**Bupropion**						
**ATC N07BA02/N06AX12**<br>or<br>**NZ ChemID 3892**	Tablet	150mg	30 or 90	2x 150mg per day.<br><br>Course of at least 7 weeks (49 days)	QTY = pack size * number dispensed	QTY/2

* 25mg patch not available on subsidy in New Zealand;
# 4mg lozenge not available on subsidy in New Zealand;
¥ 1mg gum not available on subsidy in New Zealand;
§ Spray and inhaler not available on subsidy in New Zealand.

2.4. Definition of major congenital malformations (MCMs)

We used the following data sources to identify MCMs:

- New Zealand: National Minimum Dataset and Mortality Collection, in the 18 months after birth.
- New South Wales: Admitted Patient Data Collection and Cause of Death Unit Record File in the 18 months after birth.
- Norway and Sweden, in the 12 months after birth:

© 2025 Tran DT et al. *JAMA Internal Medicine*.

- Medical birth registers capture congenital anomalies detected in the first clinical examination, during neonatal stay in intensive care unit, and diagnoses up to one year after birth in Norway; and up to three months after birth in Sweden.
- National patient registers: If a MCM is only identified in records from outpatient specialist care, it is required that diagnosis from the same subgroup was recorded in at least two separate visits.
- Cause of Death Registers.

We classified MCM subgroups based on the EUROCAT 1.4 classification,[15] with minor exceptions due to national coding practices. For New Zealand and New South Wales data, we further required the presence of ACHI codes indicating surgical repair of "undescended testes" and "talipes" to define these anomalies. In Norway and Sweden, we did not include Q65 "Congenital deformities of hip" due to concerns about low validity.

eTable 3 presents the ICD-10/ICD-10-AM codes we used to define MCM subgroups. Outcome "MCM overall" was defined as presence of any MCM subgroup as shown in eTable 3, excluding chromosomal anomalies, genetic anomalies, and congenital malformations due to teratogenic infection.

eTable 3: Definition of major congenital malformation categories (subgroups)

**Major congenital malformations subgroups**	**ICD-10 or ICD-10-AM**
**Nervous system**	Q00-Q07,<br>Except Q046.1, Q07.80, Q07.82
**Eye**	Q10-Q15<br>Except Q10.1-Q10.3, Q10.5, Q13.5
**Ear**	Q16, Q17.8
**Congenital heart defects**	Q20-Q26<br>Except Q21.11, Q24.6, Q25.0 if GA<37wk, Q25.41, Q25.6 if GA<37wk, Q26.1
**Respiratory system**	Q30.0, Q30.2, Q30.8, Q31.0-Q31.1, Q31.8,<br>Q32-Q34
**Oro-facial clefts**	Q35-Q37
**Digestive system**	Q38-Q45, Q79.0,<br>Except Q38.1, Q38.2, Q38.50, Q43.0, Q43.20, Q43.81, Q43.82, Q44.4, Q45.83
**Abdominal wall**	Q79.2, Q79.3, Q79.5
**Genital organs**	Q50.0, Q51, Q52.0-Q52.2, Q52.4, Q52.6, Q54-Q56<br><br>***Where ICD-10-AM & ACHI code available:*** Included Q53 if had surgical repair of undescended testes: *ACHI code*: 37803-00 & 37803-01
**Kidney and urinary tract**	Q60-Q64, Q79.4,<br>Except Q61.0, Q61.9, Q62.7, Q63.3
**Limb anomalies**	Q68.1, Q69-Q74<br>Q65, except Q65.3 – Q65.6<br><br>***Where ICD-10-AM & ACHI code available***: Included Q66.0, Q66.1, Q66.4 if had surgical repair of talipes: *ACHI code:* 49718-01, 49724-00, 49724-01, 49727-00, 50321-00, 50324-00, 50324-01, 50327-00
**Other congenital malformations**	
Peripheral vascular system	Q27.1, Q27.3, Q27.8, Q28;
Other congenital malformations of musculoskeletal	Q75.0, Q77.5, Q75.8, Q76, Q77, Q78.2-Q78.8, Q79.1, Q79.8,
Breast, integument or skin	Q80, Q81, Q82, Q83, Q84.0, Q84.3, Q84.4, Q85.1, Q85.8<br>Except Q82.5, Q82.8, Q83.3
Congenital hypothyroidism	E03.0, E03.1
Phenylketonuria	E70.0, E70.1
Congenital malformations of other endocrine glands	Q89.0-Q89.2
Situs inversus	Q89.3
Conjoined twins	Q89.4
Other specified congenital malformations	Q89.7, Q89.8
**Chromosomal anomaly, genetic syndrome, teratogenic infection**	***EXCLUDED exposed and unexposed pregnancies if an infant had one of these MCMs.***<br>Q90-Q93, Q96-Q99,

© 2025 Tran DT et al. *JAMA Internal Medicine*.

Major congenital malformations subgroups	ICD-10 or ICD-10-AM
	D56.0, D56.1, D82.1, Q87, Q61.9, Q75.1, Q75.4, Q78.0, Q78.1, P35.0-P35.2, P35.4, P37.1

eTable 4 presents the ICD-10/ICD-10-AM codes we used to define specific MCMs. Acknowledging that this study would unlikely achieve sufficient statistical power to assess the risk of all specific MCMs, we focused on MCMs with prevalence ≥10 per 10,000 livebirths, according to EUROCAT prevalence estimates.[16]

eTable 4: Definition of specific major congenital malformations

Specific major congenital malformation	ICD-10 or ICD-10-AM
**Congenital heart defects**	
Ventricular septal defect	Q21.0
Atrial septal defect	Q21.1, except Q21.11
Atrioventricular septal defect	Q21.2
Pulmonary valve stenosis	Q22.1
Coarctation of aorta	Q25.1
Tetralogy and pentalogy of Fallot	Q21.3
Complete transposition of great arteries	Q20.3
Patent ductus arteriosus in term infants	Q25.0 if GA ≥37weeks
Hypoplastic left heart	Q23.4
Double outlet right ventricle	Q20.1
Aortic valve atresia/stenosis	Q25.2
Single ventricle	Q20.4
Total anomalous pulmonary venous return	Q26.2
Common arterial truncus	Q20.0
Tricuspid atresia and stenosis	Q22.4
Hypoplastic right heart	Q22.6
Mitral valve atresia/stenosis	Q23.2, Q23.3
Ebstein's anomaly	Q22.5
**Limb anomalies**	
Club foot – talipes equinovarus	Q66.0<br>***Where ICD-10-AM & ACHI code available***: Included Q660, Q661, Q664 if had surgical repair of talipes: *ACHI code:* 49718-01, 49724-00, 49724-01, 49727-00, 50321-00, 50324-00, 50324-01, 50327-00
Polydactyly	Q69
Hip dislocation and/or dysplasia	Q65.0-Q65.2, Q65.80, Q65.81
Limb reduction defects	Q71-Q73
Syndactyly	Q70
**Kidney and urinary tract**	
Congenital hydronephrosis	Q62.0
Multicystic renal dysplasia	Q61.40, Q61.41
Bilateral renal agenesis including Potter sequence	Q60.1, Q60.6
Posterior urethral valve	Q64.20, Q79.4
Bladder exstrophy and/or epispadias	Q64.0, Q64.1
**Nervous system**	
Neural tube defects	Q00, Q01, Q05
Hydrocephaly	Q03
Spina bifida	Q05
Anencephaly and similar	Q00
Severe microcephaly	Q02
Agenesis of corpus callosum	Q04.01
Arhinencephaly or holoprosencephaly	Q04.1, Q04.2
Encephalocele and meningocele	Q01
**Genital organs**	
Hypospadias	Q54
Indeterminate sex	Q56
**Digestive system**	

Specific major congenital malformation	ICD-10 or ICD-10-AM
Ano-rectal atresia or/and stenosis	Q42.0-Q42.3
Diaphragmatic hernia	Q79.0
Oesophageal atresia with or without trachea-oesophageal fistula	Q39.0-Q39.1
Duodenal atresia or stenosis	Q41.0
Hirschsprung's disease	Q43.1
Atresia or stenosis of other parts of small intestine	Q41.1-Q41.8
Atresia of bile ducts	Q44.2
Annular pancreas	Q45.1
**Oro-facial clefts**	
Cleft lip with or without cleft palate	Q36, Q37
Cleft palate	Q35

2.5. Definitions of exclusion criteria

We excluded the following births.

- Multiple births: Multiple births carry elevated risk of congenital malformations, likely a mediating factor in the association between medicine exposure and MCM outcomes.
- Missing or invalid information to estimate DoC: Valid information was required to calculate DoC and to define some specific MCMs (e.g. patent ductus arteriosus in full-term birth). We excluded a birth when both date of childbirth and expected date of delivery were missing; when gestational age was missing or invalid (≥44 weeks or ≥ 314 days); or when calculation of DoC was not possible.
- Conception occurred within 6 months from a prior childbirth: Short interpregnancy intervals are associated with higher risk of congenital anomalies (likely due to folic acid depletion).[17] We calculated the interpregnancy interval as the difference between DoC and date of prior childbirth. A birth was excluded if parity ≥1 and 0≤ interval <180 days.
- Infant having a MCM due to chromosomal anomaly, genetic anomaly, and viral infections (see eTable 3 for definition of these congenital anomalies).
- Mother diagnosed with viral infections in the first trimester, identified by the following ICD-10 codes
  - A92.8 Other specified mosquito-borne viral fevers (includes zika virus),
  - B06 Rubella,
  - B25 Cytomegaloviral disease,
  - B58 Toxoplasmosis, and
  - O35.3 Maternal care for (suspected) fetal damage due to cytomegaloviral or rubella viral disease.
- Mother had a dispensing of medicines that are considered potentially teratogenic or known teratogens, based on methods of previous studies,[18,19] and category D&X medicines classified by the Australian Therapeutic Goods Administration.[20] These medicines are listed in eTable 5.
- Mother had dispensings of two or more smoking cessation pharmacotherapies in the first trimester.
- Interstate resident (NSW data) or overseas visitors (NSW and NZ data): For these women, data relating to before, during and post pregnancy were likely to be incomplete.
- Missing personal identification number for infants (unable to link infant's records): This was applicable to only a small number of infants in NZ.

© 2025 Tran DT et al. *JAMA Internal Medicine*.

eTable 5: Known and potentially teratogenic medicines

	Medicines	ATC code *
**Known teratogens**		
Vitamin K antagonists	Warfarin	B01AA03
Systematic or topical retinoid	tretinoin	D10AD01
	Isotretinoin	D10BA01 (oral), D10AD04 (topical), D10AD54 (combinations, topical)
	Etretinate	D05BB01
	Acitretin	D05BB02
	Alitretinoin	D11AH04
Synthetic prostaglandin	Misoprostol	A02BB01, G02AD06, M01AE56, M01AB55 single or combined
Immunosuppressants	Thalidomide	L04AX02
	Leflunomide	L04AA13
	Teriflunomide	L04AA31
	Lenalidomide	L04AX04
	Pomalidomide	L04AX06
	Methotrexate	L04AX03
	Mycophenolic acid	L04AA06
	Ozanimod	L04AA38
	Siponimod	L04AA42
	Tofacitinib	L04AA29
Antiepileptics	Valproate	N03AG01
Hepatitis C treatment		J05AP
Selective estrogen receptor modulators	Raloxifene	G03XC01
**Potential teratogens**		
Agents acting on the renin-angiotensin system		C09
Antipsychotics, anxiolytics and hypnotics	Lithium	N05AN01
Antiepileptics [excl. valproate]		N03A [excluding N03AG01]
Antigonadotropins and similar agents	Danazol	G03XA01
Antithyroid agents	Propylthiouracil	H03BA02
	Carbimazole	H03BB01
	Methimazole	H03BB02, H03BB52
Antineoplastic drugs		L01
Antibiotics: Tetracycline and derivatives	Tetracycline	J01AA07, J01AA20
	Tigecycline	J01AA12
	Metacycline	J01AA05
	Minocycline	J01AA08
	Oxytetracycline	J01AA06, J01AA56
Antibiotics: aminoglycosides	Amikacin	J01GB06
	Gentamicin	J01GB03
	Kanamycin	J01GB04
	Neomycin	J01GB05
	Tobramycin	J01GB01
Antiviral	Efavirenz	J05AR06
	Cidofovir	J05AB12
	Ganciclovir	J05AB06
	Valganciclovir	J05AB14
Antifungal	Fluconazole	J02AC01
Agents used in gout and hyperuricaemia	Colchicine	M04AC01
Endothelin receptor antagonist		C02KX

© 2025 Tran DT et al. *JAMA Internal Medicine*.

2.6. Definitions of covariates

eTable 6: Definitions of infant and maternal socio-demographic characteristics and obstetric variables

Covariate	Country-specific definition
Calendar year of childbirth	• Based on date of childbirth
Child's sex	• As recorded in perinatal, maternity data, medical birth registries
Maternal age at delivery	• As recorded in perinatal, maternity data, medical birth registries
Maternal Indigenous status	• Australia: Aboriginal and/or Torres Strait Islander status recorded in the most recent perinatal record; if missing supplemented by information recorded in the corresponding delivery hospital admission record<br>• New Zealand: Māori ethnicity ever recorded in perinatal, hospital admission, mortality, National Health Index or pharmaceutical dispensing records.<br>• Norway and Sweden: Indigenous status not recorded
Maternal country of birth	• Australian: Country of birth recorded in the most recent perinatal record, if missing, supplemented by information recorded in the corresponding delivery hospital admission record<br>• New Zealand: Not available<br>• Norway/Sweden: As recorded in medical birth registries or population register
Maternal relationship (have a partner)	• Australia: As recorded in the hospital admission associated with the delivery.<br>• New Zealand: Not available<br>• Norway/Sweden: As recorded in medical birth registries or population register
Maternal education	• Australia: Not available<br>• New Zealand: Not available<br>• Norway/Sweden: Education level in the year of childbirth
Socioeconomic deprivation of maternal residence	• Australia: Quintile of the Relative Socio-economic Disadvantage for the area of residence. This was based on the Australian Bureau of Statistics 2016 Census<br>• New Zealand: The New Zealand Index of Deprivation<br>• Norway/Sweden: not available
Remoteness of maternal residence	• Australia: based on the Australian Accessibility and Remoteness Index of Australia Plus, mapped to the area of maternal residence. There are five categories: major cities, inner regional, outer regional, remote and very remote.<br>• New Zealand: based on the Geographical Classification for Health which is similar to the Australian Accessibility and Remoteness Index of Australia Plus. There are five categories: Urban 1, Urban 2, Rural 1, Rural 2, and Rural 3<br>• Norway/Sweden: not available
Maternal Body Mass Index (BMI)	• Australia: Not available.<br>• New Zealand: BMI is recorded in the National Maternity Collection (some missing data).<br>• Norway/Sweden: As recorded in medical birth registries or calculated from maternal weight and height at first antenatal visit (some missing data)
Parity	• As recorded in perinatal, maternity data, medical birth registries
Older sibling(s) having a congenital malformation	• Older sibling(s) had a major congenital malformation (see eTable 4)
Maternal hospitalisation in 12 months prior to date of conception	• The number of maternal hospital admission for any reason, not counting transfers during a hospital stay

We identified maternal morbidities using perinatal, hospital admission, and dispensing data, based on prior methods.[11,21] We acknowledged that pre-existing chronic conditions are generally managed in settings other than hospitals, thus maternal morbidities may not be recorded in inpatient hospital data unless a woman was admitted due to complications in pregnancy or to give birth. Meanwhile, some chronic health conditions (e.g., asthma, gastro-oesophageal reflux) may occasionally develop an acute episode or get worse during pregnancy. Hence, when using inpatient data, we adjusted the lookback period accordingly. When analysing NZ dispensing data, NZ Chemical IDs were mapped to the listed ATC codes. See eTable 7 for ICD-10 codes, ATC code, and items from perinatal data used to identify maternal morbidities.

© 2025 Tran DT et al. *JAMA Internal Medicine*.

eTable 7: Definitions of pre-existing maternal morbidities

Maternal morbidity	ICD-10 / ICD-10-AM codes or other data sources	Dispensing data ATC codes *
	**Lookback period for ascertainment: DoC-365 to date of childbirth *(unless indicated otherwise)***	**Lookback period for ascertainment: DoC-365 to DoC-1**
Mental health disorder	F31-F34, F38, F39, F40, F41, F44, F48, F20-F25, F28-F30, O99.3,<br><br>For NZ, we additionally included the national mental health and addiction service activity and outcomes data	N05BA01 - N05BA12, N05BE01<br>N05AN01**<br>N06AA01-N06AG02, N06AX03 - N06AX11, N06AX12, N06AX13 - N06AX18, N06AX21 - N06AX26, N05AA01 - N05AB02, N05AB06 - N05AL07, N05AX07 - N05AX13
Chronic airway disorder	J31, J32, J35, J37, J40-J44, J47, R05, O99.5<br><br>***(Lookback period: DoC-365 to DoC-1)***:<br>J45, J46, J98, J99	R03AC02 - R03DC03, R03DX05
Gastro-oesophageal reflux	***(Lookback period: DoC-365 to DoC-1)***<br>K21.0, K21.9	A02BA01 - A02BX05
Use of non-steroid anti-inflammatory drugs		M01AB01 - M01AH06
Use of steroids		H02AB01 - H02AB10
Anaemia and coagulation disorders	D56-D57, D65-D68, D50-D53, D55, D58-D64	B01AA03 - B01AB06, B01AE07, B01AF01, B01AF02, B01AX05<br><br>B01AC04 - B01AC07, B01AC12 - B01AC30, B01AC09, B01AC11 †
Drug or alcohol disorders	F10 -F16, F18, F19, Z50.2, Z50.3, Z72.1, Z72.2	N07BB01 - N07BB99
Thyroid disorder	E00-E07, E89.0	H03BA02 - H03BB01<br>H03AA01 - H03AA02
Cardiovascular disease	I05-I09, I34-I39, I50, I20, I25, I27, I28, Q20-Q25, O99.4<br><br>***(lookback period: DoC-365 to DoC-1)***:<br>I00-I02, I21-I24, I26, I30-I33, I40-I43, I44-I49, I51-I52, I60-I64, G45.8, G45.9, I65, I66, I67.2, I70, I73, I74, I77	C03DA02 - C03DA99, C07AB07, C07AB12, C07AG02, [(C03CA01 - C03CC01) and (C09AA01- C09AX99 or C09CA01 - C09CX99)], C07AB02 if metoprolol succinate) ‡<br><br>C07AA01 - C07AA06, C07AA08 - C07AB01, [C07AB02 if not metoprolol succinate ‡], C07AB03, C07AG01, C08CA01 - C08DB01, C09BB02 - C09BB10, C09DB01 - C09DB04, C09DX01, C09DX03, C10BX03 §
Pre-existing diabetes	E10-E14, O24.0, O24.1, O24.2, O24.3<br><br>NSW perinatal data: a check-box for pre-existing diabetes £	A10A<br>A10BB – A10BK §§
Pre-existing hypertension	I10, I11, I12, I13, I15, O10<br><br>NSW perinatal data: a check-box for chronic hypertension £	C03AA01–C03BA11, C03DB01, C03DB99, C03EA01, C09BA02– C09BA09, C09DA01, C09DA02–C09DA08, C02AB01–C02AC05, C02DB02–C02DB99, (C09AA01- C09AX99 or C09CA01–C09CX99)‡‡
Epilepsy	G40, F80.3	N03AA01 - N03AX99
Chronic renal disease	N02-N08, N11-N12, N14-N16, N18-N19, N25-N28, Q60-Q63, N39.1, N39.2, T82.4, T86.1, Z49, Z94.0, Z99.2<br><br>***(lookback period DoC-365 to DoC-1):***<br>N00, N01, N17	A11CC01 - A11CC04, B03XA01 - B03XA03, V03AE02, V03AE03, V03AE05
Rheumatic disease	D89.1, M05, M06.1, M30.0, M30.1, M30.8, M31.3-M31.7, M32, M33.0-M33.9, M34, M35.0, M35.2, M35.3, M45, M94.1	

*: NZ Chemical IDs were mapped to these ATC codes.
** Lithium (WHO N05AN01 code) was recorded as N06AX in Australian PBS data
†: Australian data: use PBS items (05030R, 05035B, 05042J, 10111E, 10117L, 10129D, 10130E, 05751Q, 06456T) instead of B01AC09, B01AC11. These PBS items represent epoprostenol and iloprost.
‡: Australian data: use PBS items (08732N, 08733P, 08734Q, 08735R). These PBS items represent metoprolol succinate.
§ Combination product for hyperlipidaemia and ischaemic heart disease: hypertension
£: Perinatal record supplements the identification of these conditions when these conditions were not recorded in hospital records.
‡‡: Either (C09AA01- C09AX99) or (C09CA01–C09CX99) but not both.
§§ Metformin (A10BA) was not included due to its use for other conditions among women of reproductive age, such as polycystic ovary syndrome.

© 2025 Tran DT et al. *JAMA Internal Medicine*.

3. eAppendix 3: Characteristics of infants in New Zealand cohort, before and after propensity score matching

eTable 8: Characteristics of infants exposed to nicotine replacement therapy (NRT any formulation) and unexposed infants in New Zealand cohort, before and after propensity score matching

Characteristics	Before matching			After matching		
	Exposed to any NRT	Unexposed*	Diff †	Exposed to any NRT	Unexposed*	Diff † §
**Total number**	**9242**	**89659**		**8678**	**74873**	
**Year of conception**						
2007	188 (2.0%)	7333 (8.2%)	0.28	175 (2.0%)	1665 (2.2%)	0.01
2008	386 (4.2%)	8728 (9.7%)	0.22	383 (4.4%)	3773 (5.0%)	0.03
2009	614 (6.6%)	8434 (9.4%)	0.10	603 (6.9%)	5705 (7.6%)	0.03
2010	973 (10.5%)	7799 (8.7%)	0.06	929 (10.7%)	7785 (10.4%)	0.01
2011	1032 (11.2%)	7267 (8.1%)	0.10	940 (10.8%)	7255 (9.7%)	0.04
2012	976 (10.6%)	6930 (7.7%)	0.10	902 (10.4%)	6914 (9.2%)	0.04
2013	889 (9.6%)	6990 (7.8%)	0.06	832 (9.6%)	6981 (9.3%)	0.01
2014	875 (9.5%)	6784 (7.6%)	0.07	800 (9.2%)	6764 (9.0%)	0.01
2015	823 (8.9%)	6591 (7.4%)	0.06	766 (8.8%)	6583 (8.8%)	0.00
2016	805 (8.7%)	6252 (7.0%)	0.06	742 (8.6%)	6243 (8.3%)	0.01
2017	630 (6.8%)	6197 (6.9%)	0.00	613 (7.1%)	5794 (7.7%)	0.03
2018	624 (6.8%)	5636 (6.3%)	0.02	590 (6.8%)	5566 (7.4%)	0.02
2019	427 (4.6%)	4718 (5.3%)	0.03	403 (4.6%)	3845 (5.1%)	0.02
**Baby's sex**						
Male	4773 (51.6%)	46314 (51.7%)	0.00	4488 (51.7%)	38561 (51.5%)	0.00
Female	4466 (48.3%)	43318 (48.3%)	0.00	4188 (48.3%)	36290 (48.5%)	0.00
**Maternal age at childbirth (year) §**						
Under 25	2983 (32.3%)	41351 (46.1%)	0.29	2923 (33.7%)	32652 (43.6%)	0.20
25-29	2641 (28.6%)	23709 (26.4%)	0.05	2487 (28.7%)	20559 (27.5%)	0.03
30-34	2106 (22.8%)	14721 (16.4%)	0.16	1922 (22.1%)	12962 (17.3%)	0.12
35 and older	1512 (16.4%)	9878 (11.0%)	0.16	1346 (15.5%)	8700 (11.6%)	0.11
**Maternal Indigenous status ‡**						
Yes	4347 (47.0%)	60048 (67.0%)	0.41	4244 (48.9%)	48635 (65.0%)	0.33
No	4892 (52.9%)	29566 (33.0%)	0.41	4434 (51.1%)	26238 (35.0%)	0.33
**Socio-economic deprivation quintile of maternal residence**						
Quintile 1 (Least disadvantaged)	801 (8.7%)	4148 (4.6%)	0.16	701 (8.1%)	3749 (5.0%)	0.12
Quintile 2	1250 (13.5%)	7858 (8.8%)	0.15	1128 (13.0%)	6943 (9.3%)	0.12
Quintile 3	1655 (17.9%)	12917 (14.4%)	0.10	1545 (17.8%)	11144 (14.9%)	0.08
Quintile 4	2461 (26.6%)	22692 (25.3%)	0.03	2346 (27.0%)	19136 (25.6%)	0.03
Quintile 5 (Most disadvantaged)	3071 (33.2%)	41893 (46.7%)	0.28	2958 (34.1%)	33901 (45.3%)	0.23
**Remoteness of maternal residence**						
Urban 1	4973 (53.8%)	43191 (48.2%)	0.11	4626 (53.3%)	36447 (48.7%)	0.09
Urban 2	2208 (23.9%)	24038 (26.8%)	0.07	2100 (24.2%)	20001 (26.7%)	0.06
Rural 1, Rural 2, Rural 3	2052 (22.2%)	22012 (24.6%)	0.06	1952 (22.5%)	18425 (24.6%)	0.05
**Maternal body mass index (BMI)**						
Underweight (BMI <18.5)	235 (2.5%)	2082 (2.3%)	0.01	218 (2.5%)	1728 (2.3%)	0.01
Normal (BMI 18.5 to <25)	3160 (34.2%)	29280 (32.7%)	0.03	3007 (34.7%)	24143 (32.2%)	0.05
Overweight (BMI 25 to <30)	2630 (28.5%)	26488 (29.5%)	0.02	2502 (28.8%)	22139 (29.6%)	0.02
Obese (BMI >=30)	2651 (28.7%)	30326 (33.8%)	0.11	2537 (29.2%)	25535 (34.1%)	0.10
**Parity**						
Nulliparous	3915 (42.4%)	29729 (33.2%)	0.19	3544 (40.8%)	25379 (33.9%)	0.14
One	4217 (45.6%)	46528 (51.9%)	0.13	4050 (46.7%)	40487 (54.1%)	0.15
Two or more	1110 (12.0%)	13402 (14.9%)	0.09	1084 (12.5%)	9007 (12.0%)	0.01
**A previous child having a major congenital malformation**						
Nulliparous	3915 (42.4%)	29729 (33.2%)	0.19	3544 (40.8%)	25379 (33.9%)	0.14
Yes	233 (2.5%)	2709 (3.0%)	0.03	229 (2.6%)	2406 (3.2%)	0.03

© 2025 Tran DT et al. *JAMA Internal Medicine*.

Characteristics	Before matching			After matching		
	Exposed to any NRT	Unexposed*	Diff †	Exposed to any NRT	Unexposed*	Diff † §
**Total number**	**9242**	**89659**		**8678**	**74873**	
No	5094 (55.1%)	57221 (63.8%)	0.18	4905 (56.5%)	47088 (62.9%)	0.13
**Maternal hospitalisation in 12 months prior to date of conception**						
None	6822 (73.8%)	64533 (72.0%)	0.04	6407 (73.8%)	54225 (72.4%)	0.03
One	1607 (17.4%)	16583 (18.5%)	0.03	1505 (17.3%)	13550 (18.1%)	0.02
Two or more	813 (8.8%)	8543 (9.5%)	0.03	766 (8.8%)	7098 (9.5%)	0.02
**Maternal morbidity**						
Mental health disorder §	2483 (26.9%)	13162 (14.7%)	0.30	2172 (25.0%)	12275 (16.4%)	0.21
Chronic airway disorder	1927 (20.9%)	13135 (14.6%)	0.16	1740 (20.1%)	11583 (15.5%)	0.12
Gastro-oesophageal reflux	671 (7.3%)	3612 (4.0%)	0.14	590 (6.8%)	3343 (4.5%)	0.10
Use of non-steroid anti-inflammation drugs	3107 (33.6%)	21377 (23.8%)	0.22	2812 (32.4%)	19558 (26.1%)	0.14
Use of steroids	989 (10.7%)	6402 (7.1%)	0.13	890 (10.3%)	5785 (7.7%)	0.09
Anaemia and coagulation disorders	441 (4.8%)	4104 (4.6%)	0.01	417 (4.8%)	3517 (4.7%)	0.01
Drug or alcohol disorders	398 (4.3%)	3174 (3.5%)	0.04	368 (4.2%)	2792 (3.7%)	0.03
Thyroid disorder	85 (0.9%)	424 (0.5%)	0.05	72 (0.8%)	387 (0.5%)	0.04
Cardiovascular disease	218 (2.4%)	1355 (1.5%)	0.06	189 (2.2%)	1250 (1.7%)	0.04
Pre-existing diabetes	129 (1.4%)	626 (0.7%)	0.07	102 (1.2%)	595 (0.8%)	0.04
Pre-existing hypertension	102 (1.1%)	510 (0.6%)	0.06	79 (0.9%)	468 (0.6%)	0.03
Epilepsy	124 (1.3%)	682 (0.8%)	0.06	107 (1.2%)	639 (0.9%)	0.04
Chronic renal disease	133 (1.4%)	1428 (1.6%)	0.01	125 (1.4%)	1178 (1.6%)	0.01
Rheumatic disease	7 (0.1%)	20 (0.0%)	0.02	3 (0.0%)	19 (0.0%)	0.01

Data are the number (percentages) and † absolute standardised differences.

*: Unexposed infants were born to women who smoked in the first trimester and were not dispensed a prescribed smoking cessation pharmacotherapy during 90 days before conception and the first trimester. In the matched sample, exposed infants were matched to unexposed infants (1:10) on propensity score and year of conception.

‡: Māori ethnicity.

§: After matching, characteristics with absolute standardised difference >0.2 were included in conditional Poisson regression models.

© 2025 Tran DT et al. *JAMA Internal Medicine*.

eTable 9: Characteristics of infants exposed to varenicline and unexposed infants in New Zealand cohort, before and after propensity score matching

Characteristics	Before matching			After matching		
	Exposed to varenicline	Unexposed*	Diff †	Exposed to varenicline	Unexposed*	Diff †
**Total number**	**955**	**65164**		**954**	**9540**	
**Year of conception**						
2010	6 (0.6%)	7799 (12.0%)	0.48	6 (0.6%)	60 (0.6%)	0.00
2011	111 (11.6%)	7267 (11.2%)	0.01	111 (11.6%)	1110 (11.6%)	0.00
2012	183 (19.2%)	6930 (10.6%)	0.24	183 (19.2%)	1830 (19.2%)	0.00
2013	122 (12.8%)	6990 (10.7%)	0.06	122 (12.8%)	1220 (12.8%)	0.00
2014	96 (10.1%)	6784 (10.4%)	0.01	95 (10.0%)	950 (10.0%)	0.00
2015	134 (14.0%)	6591 (10.1%)	0.12	134 (14.0%)	1340 (14.0%)	0.00
2016	90 (9.4%)	6252 (9.6%)	0.01	90 (9.4%)	900 (9.4%)	0.00
2017	87 (9.1%)	6197 (9.5%)	0.01	87 (9.1%)	870 (9.1%)	0.00
2018	71 (7.4%)	5636 (8.6%)	0.04	71 (7.4%)	710 (7.4%)	0.00
2019	55 (5.8%)	4718 (7.2%)	0.06	55 (5.8%)	550 (5.8%)	0.00
**Baby's sex**						
Male	487 (51.0%)	33553 (51.5%)	0.01	487 (51.0%)	4777 (50.1%)	0.02
Female	468 (49.0%)	31591 (48.5%)	0.01	467 (49.0%)	4763 (49.9%)	0.02
**Maternal age at childbirth (year)**						
Under 25	212 (22.2%)	29217 (44.8%)	0.49	212 (22.2%)	2059 (21.6%)	0.02
25-29	322 (33.7%)	18032 (27.7%)	0.13	322 (33.8%)	3373 (35.4%)	0.03
30-34	244 (25.5%)	10898 (16.7%)	0.22	244 (25.6%)	2425 (25.4%)	0.00
35 and older	177 (18.5%)	7017 (10.8%)	0.22	176 (18.4%)	1683 (17.6%)	0.02
**Maternal Indigenous status ‡**						
Yes	441 (46.2%)	44765 (68.7%)	0.47	441 (46.2%)	4344 (45.5%)	0.01
No	513 (53.7%)	20369 (31.3%)	0.47	513 (53.8%)	5196 (54.5%)	0.01
**Socio-economic deprivation quintile of maternal residence**						
Quintile 1 (Least disadvantaged)	73 (7.6%)	2867 (4.4%)	0.14	73 (7.7%)	760 (8.0%)	0.01
Quintile 2	123 (12.9%)	5463 (8.4%)	0.15	123 (12.9%)	1258 (13.2%)	0.01
Quintile 3	180 (18.8%)	9057 (13.9%)	0.13	180 (18.9%)	1817 (19.0%)	0.00
Quintile 4	258 (27.0%)	16355 (25.1%)	0.04	257 (26.9%)	2500 (26.2%)	0.02
Quintile 5 (Most disadvantaged)	321 (33.6%)	31319 (48.1%)	0.30	321 (33.6%)	3205 (33.6%)	0.00
**Remoteness of maternal residence**						
Urban 1	491 (51.4%)	30761 (47.2%)	0.08	491 (51.5%)	4841 (50.7%)	0.01
Urban 2	238 (24.9%)	17781 (27.3%)	0.05	238 (24.9%)	2482 (26.0%)	0.02
Rural 1, Rural 2, Rural 3	226 (23.7%)	16526 (25.4%)	0.04	225 (23.6%)	2217 (23.2%)	0.01
**Maternal body mass index (BMI)**						
Underweight (BMI <18.5)	11 (1.2%)	1420 (2.2%)	0.08	11 (1.2%)	95 (1.0%)	0.02
Normal (BMI 18.5 to <25)	321 (33.6%)	20337 (31.2%)	0.05	321 (33.6%)	3313 (34.7%)	0.02
Overweight (BMI 25 to <30)	289 (30.3%)	19348 (29.7%)	0.01	289 (30.3%)	2822 (29.6%)	0.02
Obese (BMI >=30)	298 (31.2%)	23522 (36.1%)	0.10	298 (31.2%)	3120 (32.7%)	0.03
**Parity**						
Nulliparous	285 (29.8%)	20689 (31.7%)	0.04	284 (29.8%)	2683 (28.1%)	0.04
One	575 (60.2%)	37889 (58.1%)	0.04	575 (60.3%)	5975 (62.6%)	0.05
Two or more	95 (9.9%)	6586 (10.1%)	0.01	95 (10.0%)	882 (9.2%)	0.02
**A previous child having a major congenital malformation**						
Nulliparous	285 (29.8%)	20689 (31.7%)	0.04	284 (29.8%)	2683 (28.1%)	0.04
Yes	31 (3.2%)	2328 (3.6%)	0.02	31 (3.2%)	300 (3.1%)	0.01
No	639 (66.9%)	42147 (64.7%)	0.05	639 (67.0%)	6557 (68.7%)	0.04
**Maternal hospitalisation in 12 months prior to date of conception**						
None	712 (74.6%)	46714 (71.7%)	0.06	711 (74.5%)	7130 (74.7%)	0.00
One	167 (17.5%)	12021 (18.4%)	0.03	167 (17.5%)	1641 (17.2%)	0.01
Two or more	76 (8.0%)	6429 (9.9%)	0.07	76 (8.0%)	769 (8.1%)	0.00

© 2025 Tran DT et al. *JAMA Internal Medicine*.

Characteristics	Before matching			After matching		
	Exposed to varenicline	Unexposed*	Diff †	Exposed to varenicline	Unexposed*	Diff †
**Total number**	**955**	**65164**		**954**	**9540**	
**Maternal morbidity**						
Mental health disorder	276 (28.9%)	10168 (15.6%)	0.32	276 (28.9%)	2752 (28.8%)	0.00
Chronic airway disorder	196 (20.5%)	9529 (14.6%)	0.16	196 (20.5%)	1981 (20.8%)	0.01
Gastro-oesophageal reflux	72 (7.5%)	2829 (4.3%)	0.14	72 (7.5%)	709 (7.4%)	0.00
Use of non-steroid anti-inflammation drugs	346 (36.2%)	16933 (26.0%)	0.22	346 (36.3%)	3476 (36.4%)	0.00
Use of steroids	129 (13.5%)	4857 (7.5%)	0.20	129 (13.5%)	1261 (13.2%)	0.01
Anaemia and coagulation disorders	48 (5.0%)	3243 (5.0%)	0.00	48 (5.0%)	456 (4.8%)	0.01
Drug or alcohol disorders	19 (2.0%)	2467 (3.8%)	0.11	19 (2.0%)	201 (2.1%)	0.01
Thyroid disorder	9 (0.9%)	313 (0.5%)	0.06	9 (0.9%)	86 (0.9%)	0.00
Cardiovascular disease	28 (2.9%)	1080 (1.7%)	0.09	28 (2.9%)	260 (2.7%)	0.01
Pre-existing diabetes	28 (2.9%)	535 (0.8%)	0.16	28 (2.9%)	245 (2.6%)	0.02
Pre-existing hypertension	8 (0.8%)	393 (0.6%)	0.03	8 (0.8%)	59 (0.6%)	0.03
Epilepsy	10 (1.0%)	537 (0.8%)	0.02	10 (1.0%)	94 (1.0%)	0.01
Chronic renal disease	10 (1.0%)	1076 (1.7%)	0.05	10 (1.0%)	101 (1.1%)	0.00
Rheumatic disease	0 (0%)	15 (0%)	0.00	0 (0%)	15 (0%)	-

Data are the number (percentages) and † absolute standardised differences.

*: Unexposed infants were born to women who smoked in the first trimester and were not dispensed a prescribed smoking cessation pharmacotherapy during 90 days before conception and the first trimester. In the matched sample, exposed infants were matched to unexposed infants (1:10) on propensity score and year of conception.

‡: Māori ethnicity.

© 2025 Tran DT et al. *JAMA Internal Medicine*.

eTable 10: Characteristics of infants exposed to bupropion and unexposed infants in New Zealand cohort, before and after propensity score matching

Characteristics	Before matching			After matching		
	Exposed to bupropion	Unexposed*	Diff †	Exposed to bupropion	Unexposed*	Diff †
**Total number**	**754**	**73598**		**749**	**7410**	
**Year of conception**						
2009	33 (4.4%)	8434 (11.5%)	0.26	33 (4.4%)	330 (4.5%)	0.00
2010	133 (17.6%)	7799 (10.6%)	0.20	132 (17.6%)	1275 (17.2%)	0.01
2011	100 (13.3%)	7267 (9.9%)	0.11	100 (13.4%)	982 (13.3%)	0.00
2012	120 (15.9%)	6930 (9.4%)	0.20	119 (15.9%)	1190 (16.1%)	0.00
2013	69 (9.2%)	6990 (9.5%)	0.01	69 (9.2%)	690 (9.3%)	0.00
2014	71 (9.4%)	6784 (9.2%)	0.01	71 (9.5%)	702 (9.5%)	0.00
2015	50 (6.6%)	6591 (9.0%)	0.09	50 (6.7%)	500 (6.7%)	0.00
2016	54 (7.2%)	6252 (8.5%)	0.05	52 (6.9%)	520 (7.0%)	0.00
2017	44 (5.8%)	6197 (8.4%)	0.10	44 (5.9%)	440 (5.9%)	0.00
2018	45 (6.0%)	5636 (7.7%)	0.07	45 (6.0%)	441 (6.0%)	0.00
2019	35 (4.6%)	4718 (6.4%)	0.08	34 (4.5%)	340 (4.6%)	0.00
**Baby's sex**						
Male	382 (50.7%)	37912 (51.5%)	0.02	380 (50.7%)	3759 (50.7%)	0.00
Female	372 (49.3%)	35665 (48.5%)	0.02	369 (49.3%)	3651 (49.3%)	0.00
**Maternal age at childbirth (year)**						
Under 25	154 (20.4%)	33393 (45.4%)	0.55	154 (20.6%)	2040 (27.5%)	0.16
25-29	233 (30.9%)	20021 (27.2%)	0.08	233 (31.1%)	2243 (30.3%)	0.02
30-34	218 (28.9%)	12179 (16.5%)	0.30	215 (28.7%)	1865 (25.2%)	0.08
35 and older	149 (19.8%)	8005 (10.9%)	0.25	147 (19.6%)	1262 (17.0%)	0.07
**Maternal Indigenous status ‡**						
Yes	319 (42.3%)	50086 (68.1%)	0.54	319 (42.6%)	3424 (46.2%)	0.07
No	435 (57.7%)	23479 (31.9%)	0.54	430 (57.4%)	3986 (53.8%)	0.07
**Socio-economic deprivation quintile of maternal residence**						
Quintile 1 (Least disadvantaged)	63 (8.4%)	3311 (4.5%)	0.16	62 (8.3%)	569 (7.7%)	0.02
Quintile 2	107 (14.2%)	6326 (8.6%)	0.18	106 (14.2%)	960 (13.0%)	0.03
Quintile 3	151 (20.0%)	10389 (14.1%)	0.16	150 (20.0%)	1426 (19.2%)	0.02
Quintile 4	183 (24.3%)	18476 (25.1%)	0.02	182 (24.3%)	1908 (25.7%)	0.03
Quintile 5 (Most disadvantaged)	250 (33.2%)	34984 (47.5%)	0.30	249 (33.2%)	2547 (34.4%)	0.02
**Remoteness of maternal residence**						
Urban 1	371 (49.2%)	35056 (47.6%)	0.03	368 (49.1%)	3655 (49.3%)	0.00
Urban 2	202 (26.8%)	19990 (27.2%)	0.01	202 (27.0%)	2003 (27.0%)	0.00
Rural 1, Rural 2, Rural 3	181 (24.0%)	18448 (25.1%)	0.02	179 (23.9%)	1752 (23.6%)	0.01
**Maternal body mass index (BMI)**						
Underweight (BMI <18.5)	13 (1.7%)	1634 (2.2%)	0.04	13 (1.7%)	155 (2.1%)	0.03
Normal (BMI 18.5 to <25)	257 (34.1%)	23347 (31.7%)	0.05	257 (34.3%)	2620 (35.4%)	0.02
Overweight (BMI 25 to <30)	216 (28.6%)	21824 (29.7%)	0.02	215 (28.7%)	2108 (28.4%)	0.01
Obese (BMI >=30)	237 (31.4%)	25992 (35.3%)	0.08	237 (31.6%)	2342 (31.6%)	0.00
**Parity**						
Nulliparous	248 (32.9%)	23711 (32.2%)	0.01	246 (32.8%)	2313 (31.2%)	0.03
One	416 (55.2%)	41349 (56.2%)	0.02	414 (55.3%)	4044 (54.6%)	0.01
Two or more	90 (11.9%)	8538 (11.6%)	0.01	89 (11.9%)	1053 (14.2%)	0.07
**A previous child having a major congenital malformation**						
Nulliparous	248 (32.9%)	23711 (32.2%)	0.01	246 (32.8%)	2313 (31.2%)	0.03
Yes	22 (2.9%)	2480 (3.4%)	0.03	22 (2.9%)	216 (2.9%)	0.00
No	484 (64.2%)	47407 (64.4%)	0.00	481 (64.2%)	4881 (65.9%)	0.03
**Maternal hospitalisation in 12 months prior to date of conception**						
None	563 (74.7%)	52788 (71.7%)	0.07	559 (74.6%)	5356 (72.3%)	0.05
One	129 (17.1%)	13569 (18.4%)	0.03	128 (17.1%)	1312 (17.7%)	0.02
Two or more	62 (8.2%)	7241 (9.8%)	0.06	62 (8.3%)	742 (10.0%)	0.06

© 2025 Tran DT et al. *JAMA Internal Medicine*.

Characteristics	Before matching			After matching		
	Exposed to bupropion	Unexposed*	Diff †	Exposed to bupropion	Unexposed*	Diff †
**Total number**	**754**	**73598**		**749**	**7410**	
**Maternal morbidity**						
Mental health disorder	661 (87.7%)	11297 (15.3%)	2.10	656 (87.6%)	6399 (86.4%)	0.04
Chronic airway disorder	201 (26.7%)	10812 (14.7%)	0.30	199 (26.6%)	1852 (25.0%)	0.04
Gastro-oesophageal reflux	72 (9.5%)	3118 (4.2%)	0.21	70 (9.3%)	648 (8.7%)	0.02
Use of non-steroid anti-inflammation drugs	280 (37.1%)	18708 (25.4%)	0.25	279 (37.2%)	2710 (36.6%)	0.01
Use of steroids	95 (12.6%)	5480 (7.4%)	0.17	94 (12.6%)	883 (11.9%)	0.02
Anaemia and coagulation disorders	35 (4.6%)	3491 (4.7%)	0.00	34 (4.5%)	318 (4.3%)	0.01
Drug or alcohol disorders	23 (3.1%)	2729 (3.7%)	0.04	23 (3.1%)	339 (4.6%)	0.08
Thyroid disorder	9 (1.2%)	346 (0.5%)	0.08	9 (1.2%)	66 (0.9%)	0.03
Cardiovascular disease	17 (2.3%)	1165 (1.6%)	0.05	16 (2.1%)	159 (2.1%)	0.00
Pre-existing diabetes	9 (1.2%)	565 (0.8%)	0.04	9 (1.2%)	71 (1.0%)	0.02
Pre-existing hypertension	7 (0.9%)	426 (0.6%)	0.04	7 (0.9%)	67 (0.9%)	0.00
Epilepsy	23 (3.1%)	598 (0.8%)	0.16	22 (2.9%)	200 (2.7%)	0.01
Chronic renal disease	6 (0.8%)	1182 (1.6%)	0.07	6 (0.8%)	76 (1.0%)	0.02
Rheumatic disease	≈3 (0.4%)	16 (0.02%)	0.08	0 (0.0%)	6 (0.08%)	0.04

Data are the number (percentages) and † absolute standardised differences.

*: Unexposed infants were born to women who smoked in the first trimester and were not dispensed a prescribed smoking cessation pharmacotherapy during 90 days before conception and the first trimester. In the matched sample, exposed infants were matched to unexposed infants (1:10) on propensity score and year of conception.

‡: Māori ethnicity.

≈: For data privacy, cell value <3 is not shown, and was replaced with a 3; percentage was adjusted accordingly.

© 2025 Tran DT et al. *JAMA Internal Medicine*.

eTable 11: Characteristics of infants exposed to transdermal nicotine patches only and unexposed infants in New Zealand cohort, before and after propensity score matching

Characteristics	Before matching			After matching		
	Exposed to patches only	Unexposed*	Diff †	Exposed to patches only	Unexposed*	Diff †
**Total number**	**3546**	**89659**		**3526**	**35194**	
**Year of conception**						
2007	137 (3.9%)	7333 (8.2%)	0.18	132 (3.7%)	1293 (3.7%)	0.00
2008	208 (5.9%)	8728 (9.7%)	0.14	208 (5.9%)	2072 (5.9%)	0.00
2009	217 (6.1%)	8434 (9.4%)	0.12	216 (6.1%)	2151 (6.1%)	0.00
2010	380 (10.7%)	7799 (8.7%)	0.07	379 (10.7%)	3777 (10.7%)	0.00
2011	385 (10.9%)	7267 (8.1%)	0.09	383 (10.9%)	3830 (10.9%)	0.00
2012	371 (10.5%)	6930 (7.7%)	0.10	370 (10.5%)	3700 (10.5%)	0.00
2013	366 (10.3%)	6990 (7.8%)	0.09	366 (10.4%)	3660 (10.4%)	0.00
2014	309 (8.7%)	6784 (7.6%)	0.04	307 (8.7%)	3070 (8.7%)	0.00
2015	312 (8.8%)	6591 (7.4%)	0.05	309 (8.8%)	3090 (8.8%)	0.00
2016	305 (8.6%)	6252 (7%)	0.06	305 (8.7%)	3041 (8.6%)	0.00
2017	205 (5.8%)	6197 (6.9%)	0.05	205 (5.8%)	2050 (5.8%)	0.00
2018	208 (5.9%)	5636 (6.3%)	0.02	205 (5.8%)	2050 (5.8%)	0.00
2019	143 (4%)	4718 (5.3%)	0.06	141 (4%)	1410 (4%)	0.00
**Baby's sex**						
Male	1815 (51.2%)	46314 (51.7%)	0.01	1805 (51.2%)	17943 (51%)	0.00
Female	1731 (48.8%)	43318 (48.3%)	0.01	1721 (48.8%)	17251 (49%)	0.00
**Maternal age at childbirth (year)**						
Under 25	1091 (30.8%)	41351 (46.1%)	0.32	1086 (30.8%)	11190 (31.8%)	0.02
25-29	1042 (29.4%)	23709 (26.4%)	0.07	1040 (29.5%)	10675 (30.3%)	0.02
30-34	858 (24.2%)	14721 (16.4%)	0.19	853 (24.2%)	8224 (23.4%)	0.02
35 and older	555 (15.7%)	9878 (11%)	0.14	547 (15.5%)	5105 (14.5%)	0.03
**Maternal Indigenous status ‡**						
Yes	1659 (46.8%)	60048 (67%)	0.42	1655 (46.9%)	17121 (48.6%)	0.03
No	1885 (53.2%)	29566 (33%)	0.42	1871 (53.1%)	18073 (51.4%)	0.03
**Socio-economic deprivation quintile of maternal residence**						
Quintile 1 (Least disadvantaged)	299 (8.4%)	4148 (4.6%)	0.15	298 (8.5%)	2660 (7.6%)	0.03
Quintile 2	446 (12.6%)	7858 (8.8%)	0.12	442 (12.5%)	4248 (12.1%)	0.01
Quintile 3	643 (18.1%)	12917 (14.4%)	0.10	637 (18.1%)	6386 (18.1%)	0.00
Quintile 4	953 (26.9%)	22692 (25.3%)	0.04	948 (26.9%)	9607 (27.3%)	0.01
Quintile 5 (Most disadvantaged)	1202 (33.9%)	41893 (46.7%)	0.26	1201 (34.1%)	12293 (34.9%)	0.02
**Remoteness of maternal residence**						
Urban 1	529 (14.9%)	13491 (15%)	0.00	528 (15%)	5385 (15.3%)	0.01
Urban 2	234 (6.6%)	7165 (8%)	0.05	233 (6.6%)	2333 (6.6%)	0.00
Rural 1, Rural 2, Rural 3	2775 (78.3%)	68585 (76.5%)	0.04	2765 (78.4%)	27476 (78.1%)	0.01
**Maternal body mass index (BMI)**						
Underweight (BMI <18.5)	99 (2.8%)	2082 (2.3%)	0.03	99 (2.8%)	948 (2.7%)	0.01
Normal (BMI 18.5 to <25)	1281 (36.1%)	29280 (32.7%)	0.07	1278 (36.2%)	12762 (36.3%)	0.00
Overweight (BMI 25 to <30)	999 (28.2%)	26488 (29.5%)	0.03	996 (28.2%)	10211 (29%)	0.02
Obese (BMI >=30)	964 (27.2%)	30326 (33.8%)	0.14	962 (27.3%)	10178 (28.9%)	0.04
**Parity**						
Nulliparous	1441 (40.6%)	29729 (33.2%)	0.16	1430 (40.6%)	13359 (38%)	0.05
One	1640 (46.2%)	46528 (51.9%)	0.11	1635 (46.4%)	17471 (49.6%)	0.07
Two or more	465 (13.1%)	13402 (14.9%)	0.05	461 (13.1%)	4364 (12.4%)	0.02
**A previous child having a major congenital malformation**						
Nulliparous	1441 (40.6%)	29729 (33.2%)	0.16	1430 (40.6%)	13359 (38%)	0.05
Yes	97 (2.7%)	2709 (3%)	0.02	97 (2.8%)	1016 (2.9%)	0.01
No	2008 (56.6%)	57221 (63.8%)	0.15	1999 (56.7%)	20819 (59.2%)	0.05
**Maternal hospitalisation in 12 months prior to date of conception**						
None	2629 (74.1%)	64533 (72%)	0.05	2616 (74.2%)	26104 (74.2%)	0.00

© 2025 Tran DT et al. *JAMA Internal Medicine*.

Characteristics	Before matching			After matching		
	Exposed to patches only	Unexposed*	Diff †	Exposed to patches only	Unexposed*	Diff †
**Total number**	**3546**	**89659**		**3526**	**35194**	
One	604 (17%)	16583 (18.5%)	0.04	599 (17%)	5982 (17%)	0.00
Two or more	313 (8.8%)	8543 (9.5%)	0.02	311 (8.8%)	3108 (8.8%)	0.00
**Maternal morbidity**						
Mental health disorder	869 (24.5%)	13162 (14.7%)	0.25	859 (24.4%)	8126 (23.1%)	0.03
Chronic airway disorder	753 (21.2%)	13135 (14.6%)	0.17	743 (21.1%)	6984 (19.8%)	0.03
Gastro-oesophageal reflux	247 (7%)	3612 (4%)	0.13	244 (6.9%)	2218 (6.3%)	0.02
Use of non-steroid anti-inflammation drugs	1168 (32.9%)	21377 (23.8%)	0.20	1160 (32.9%)	11547 (32.8%)	0.00
Use of steroids	391 (11%)	6402 (7.1%)	0.14	387 (11%)	3601 (10.2%)	0.02
Anaemia and coagulation disorders	161 (4.5%)	4104 (4.6%)	0.00	160 (4.5%)	1493 (4.2%)	0.01
Drug or alcohol disorders	117 (3.3%)	3174 (3.5%)	0.01	117 (3.3%)	1159 (3.3%)	0.00
Thyroid disorder	38 (1.1%)	424 (0.5%)	0.07	37 (1%)	294 (0.8%)	0.02
Cardiovascular disease	79 (2.2%)	1355 (1.5%)	0.05	76 (2.2%)	705 (2%)	0.01
Pre-existing diabetes	52 (1.5%)	626 (0.7%)	0.07	51 (1.4%)	437 (1.2%)	0.02
Pre-existing hypertension	39 (1.1%)	510 (0.6%)	0.06	37 (1%)	315 (0.9%)	0.02
Epilepsy	55 (1.6%)	682 (0.8%)	0.07	55 (1.6%)	449 (1.3%)	0.02
Chronic renal disease	53 (1.5%)	1428 (1.6%)	0.01	53 (1.5%)	540 (1.5%)	0.00
Rheumatic disease	4 (0.1%)	20 (0.0%)	0.03	4 (0.1%)	18 (0.1%)	0.02

Data are the number (percentages) and † absolute standardised differences.

*: Unexposed infants were born to women who smoked in the first trimester and were not dispensed a prescribed smoking cessation pharmacotherapy during 90 days before conception and the first trimester. In the matched sample, exposed infants were matched to unexposed infants (1:10) on propensity score and year of conception.

‡: Māori ethnicity.

© 2025 Tran DT et al. *JAMA Internal Medicine*.

eTable 12: Characteristics of infants exposed to fast-acting nicotine formulations (lozenges, gums) only and unexposed infants in New Zealand cohort, before and after propensity score matching

Characteristics	Before matching			After matching		
	Exposed to fast-acting nicotine formulations only	Unexposed*	Diff †	Exposed to fast-acting nicotine formulations only	Unexposed*	Diff †
**Total number**	**2454**	**89659**		**2435**	**24290**	
**Year of conception**						
2007	34 (1.4%)	7333 (8.2%)	0.32	34 (1.4%)	340 (1.4%)	0.00
2008	106 (4.3%)	8728 (9.7%)	0.21	106 (4.4%)	1051 (4.3%)	0.00
2009	218 (8.9%)	8434 (9.4%)	0.02	218 (9%)	2153 (8.9%)	0.00
2010	248 (10.1%)	7799 (8.7%)	0.05	247 (10.1%)	2463 (10.1%)	0.00
2011	259 (10.6%)	7267 (8.1%)	0.08	259 (10.6%)	2573 (10.6%)	0.00
2012	233 (9.5%)	6930 (7.7%)	0.06	228 (9.4%)	2280 (9.4%)	0.00
2013	218 (8.9%)	6990 (7.8%)	0.04	218 (9%)	2180 (9%)	0.00
2014	219 (8.9%)	6784 (7.6%)	0.05	216 (8.9%)	2160 (8.9%)	0.00
2015	186 (7.6%)	6591 (7.4%)	0.01	183 (7.5%)	1830 (7.5%)	0.00
2016	199 (8.1%)	6252 (7%)	0.04	197 (8.1%)	1970 (8.1%)	0.00
2017	208 (8.5%)	6197 (6.9%)	0.06	207 (8.5%)	2070 (8.5%)	0.00
2018	190 (7.7%)	5636 (6.3%)	0.06	187 (7.7%)	1870 (7.7%)	0.00
2019	136 (5.5%)	4718 (5.3%)	0.01	135 (5.5%)	1350 (5.6%)	0.00
**Baby's sex**						
Male	1268 (51.7%)	46314 (51.7%)	0.00	1261 (51.8%)	12592 (51.8%)	0.00
Female	1185 (48.3%)	43318 (48.3%)	0.00	1173 (48.2%)	11688 (48.1%)	0.00
**Maternal age at childbirth (year)**						
Under 25	836 (34.1%)	41351 (46.1%)	0.25	836 (34.3%)	8389 (34.5%)	0.00
25-29	692 (28.2%)	23709 (26.4%)	0.04	689 (28.3%)	7166 (29.5%)	0.03
30-34	517 (21.1%)	14721 (16.4%)	0.12	511 (21%)	5037 (20.7%)	0.01
35 and older	409 (16.7%)	9878 (11%)	0.16	399 (16.4%)	3698 (15.2%)	0.03
**Maternal Indigenous status** ‡						
Yes	1113 (45.4%)	60048 (67%)	0.45	1109 (45.5%)	11382 (46.9%)	0.03
No	1340 (54.6%)	29566 (33%)	0.45	1326 (54.5%)	12908 (53.1%)	0.03
**Socio-economic deprivation quintile of maternal residence**						
Quintile 1 (Least disadvantaged)	230 (9.4%)	4148 (4.6%)	0.19	225 (9.2%)	2062 (8.5%)	0.03
Quintile 2	339 (13.8%)	7858 (8.8%)	0.16	338 (13.9%)	3341 (13.8%)	0.00
Quintile 3	471 (19.2%)	12917 (14.4%)	0.13	469 (19.3%)	4672 (19.2%)	0.00
Quintile 4	639 (26%)	22692 (25.3%)	0.02	634 (26%)	6539 (26.9%)	0.02
Quintile 5 (Most disadvantaged)	774 (31.5%)	41893 (46.7%)	0.31	769 (31.6%)	7676 (31.6%)	0.00
**Remoteness of maternal residence**						
Urban 1	362 (14.8%)	13491 (15%)	0.01	362 (14.9%)	3724 (15.3%)	0.01
Urban 2	167 (6.8%)	7165 (8%)	0.05	166 (6.8%)	1635 (6.7%)	0.00
Rural 1, Rural 2, Rural 3	1924 (78.4%)	68585 (76.5%)	0.05	1907 (78.3%)	18931 (77.9%)	0.01
**Maternal body mass index (BMI)**						
Underweight (BMI <18.5)	60 (2.4%)	2082 (2.3%)	0.01	60 (2.5%)	578 (2.4%)	0.01
Normal (BMI 18.5 to <25)	854 (34.8%)	29280 (32.7%)	0.05	854 (35.1%)	8545 (35.2%)	0.00
Overweight (BMI 25 to <30)	694 (28.3%)	26488 (29.5%)	0.03	693 (28.5%)	6920 (28.5%)	0.00
Obese (BMI >=30)	680 (27.7%)	30326 (33.8%)	0.13	679 (27.9%)	7412 (30.5%)	0.06
**Parity**						
Nulliparous	1151 (46.9%)	29729 (33.2%)	0.28	1134 (46.6%)	10807 (44.5%)	0.04
One	1044 (42.5%)	46528 (51.9%)	0.19	1043 (42.8%)	11182 (46%)	0.06
Two or more	259 (10.6%)	13402 (14.9%)	0.13	258 (10.6%)	2301 (9.5%)	0.04
**A previous child having a major congenital malformation**						
Nulliparous	1151 (46.9%)	29729 (33.2%)	0.28	1134 (46.6%)	10807 (44.5%)	0.04
Yes	51 (2.1%)	2709 (3%)	0.06	51 (2.1%)	521 (2.1%)	0.00
No	1252 (51%)	57221 (63.8%)	0.26	1250 (51.3%)	12962 (53.4%)	0.04

© 2025 Tran DT et al. *JAMA Internal Medicine*.

Characteristics	Before matching			After matching		
	Exposed to fast-acting nicotine formulations only	Unexposed*	Diff †	Exposed to fast-acting nicotine formulations only	Unexposed*	Diff †
**Total number**	**2454**	**89659**		**2435**	**24290**	
**Maternal hospitalisation in 12 months prior to date of conception**						
None	1842 (75.1%)	64533 (72%)	0.07	1828 (75.1%)	18289 (75.3%)	0.01
One	406 (16.5%)	16583 (18.5%)	0.05	403 (16.6%)	3988 (16.4%)	0.00
Two or more	206 (8.4%)	8543 (9.5%)	0.04	204 (8.4%)	2013 (8.3%)	0.00
**Maternal morbidity**						
Mental health disorder	652 (26.6%)	13162 (14.7%)	0.30	642 (26.4%)	6390 (26.3%)	0.00
Chronic airway disorder	524 (21.4%)	13135 (14.6%)	0.18	517 (21.2%)	5103 (21%)	0.01
Gastro-oesophageal reflux	163 (6.6%)	3612 (4%)	0.12	161 (6.6%)	1620 (6.7%)	0.00
Use of non-steroid anti-inflammation drugs	832 (33.9%)	21377 (23.8%)	0.22	822 (33.8%)	8568 (35.3%)	0.03
Use of steroids	263 (10.7%)	6402 (7.1%)	0.13	262 (10.8%)	2581 (10.6%)	0.00
Anaemia and coagulation disorders	110 (4.5%)	4104 (4.6%)	0.00	108 (4.4%)	1032 (4.2%)	0.01
Drug or alcohol disorders	120 (4.9%)	3174 (3.5%)	0.07	117 (4.8%)	1160 (4.8%)	0.00
Thyroid disorder	18 (0.7%)	424 (0.5%)	0.03	18 (0.7%)	161 (0.7%)	0.01
Cardiovascular disease	61 (2.5%)	1355 (1.5%)	0.07	59 (2.4%)	573 (2.4%)	0.00
Pre-existing diabetes	38 (1.5%)	626 (0.7%)	0.08	35 (1.4%)	364 (1.5%)	0.01
Pre-existing hypertension	27 (1.1%)	510 (0.6%)	0.06	25 (1%)	217 (0.9%)	0.01
Epilepsy	35 (1.4%)	682 (0.8%)	0.06	33 (1.4%)	336 (1.4%)	0.00
Chronic renal disease	35 (1.4%)	1428 (1.6%)	0.01	35 (1.4%)	344 (1.4%)	0.00
Rheumatic disease	≈3 (0.1%)	20 (0.0%)	0.04	≈3 (0.1%)	19 (0.1%)	0.01

Data are the number (percentages) and † absolute standardised differences.

*: Unexposed infants were born to women who smoked in the first trimester and were not dispensed a prescribed smoking cessation pharmacotherapy during 90 days before conception and the first trimester. In the matched sample, exposed infants were matched to unexposed infants (1:10) on propensity score and year of conception.

‡: Māori ethnicity.

≈: For data privacy, cell value <3 is not shown, and was replaced with a 3; percentage was adjusted accordingly.

© 2025 Tran DT et al. *JAMA Internal Medicine*.

4. eAppendix 4: Characteristics of infants in New South Wales cohort, Australia, before and after propensity score matching

eTable 13: Characteristics of infants exposed to transdermal nicotine patches and unexposed infants in New South Wales cohort, Australia, before and after propensity score matching

Characteristics	Before matching			After matching		
	Exposed to transdermal nicotine patches	Unexposed*	Diff †	Exposed to transdermal nicotine patches	Unexposed*	Diff †
**Total number**	**647**	**67379**		**647**	**6455**	
**Year of conception**						
2010	8 (1.2%)	17808 (26.4%)	0.78	8 (1.2%)	80 (1.2%)	0.00
2011	92 (14.2%)	8022 (11.9%)	0.07	92 (14.2%)	920 (14.3%)	0.00
2012	90 (13.9%)	7257 (10.8%)	0.10	90 (13.9%)	900 (13.9%)	0.00
2013	81 (12.5%)	7003 (10.4%)	0.07	81 (12.5%)	810 (12.5%)	0.00
2014	72 (11.1%)	6705 (10.0%)	0.04	72 (11.1%)	720 (11.2%)	0.00
2015	83 (12.8%)	6160 (9.1%)	0.12	83 (12.8%)	830 (12.9%)	0.00
2016	105 (16.2%)	6274 (9.3%)	0.21	105 (16.2%)	1041 (16.1%)	0.00
2017	105 (16.2%)	6548 (9.7%)	0.19	105 (16.2%)	1047 (16.2%)	0.00
2018	11 (1.7%)	1602 (2.4%)	0.05	11 (1.7%)	107 (1.7%)	0.00
**Baby's sex**						
Male	329 (50.9%)	34680 (51.5%)	0.01	329 (50.9%)	3233 (50.1%)	0.02
Female	318 (49.1%)	32696 (48.5%)	0.01	318 (49.1%)	3222 (49.9%)	0.02
**Maternal age at childbirth (year)**						
Under 25	181 (28.0%)	23981 (35.6%)	0.16	181 (28.0%)	1760 (27.3%)	0.02
25-29	175 (27.0%)	18965 (28.1%)	0.02	175 (27.0%)	1844 (28.6%)	0.03
30-34	179 (27.7%)	14550 (21.6%)	0.14	179 (27.7%)	1744 (27.0%)	0.01
35 and older	112 (17.3%)	9882 (14.7%)	0.07	112 (17.3%)	1107 (17.1%)	0.00
**Maternal Indigenous status ‡**						
Yes	143 (22.1%)	13421 (19.9%)	0.05	143 (22.1%)	1359 (21.1%)	0.03
No	504 (77.9%)	53928 (80.0%)	0.05	504 (77.9%)	5096 (78.9%)	0.03
**Maternal country of birth**						
Native born	590 (91.2%)	59259 (87.9%)	0.11	590 (91.2%)	5989 (92.8%)	0.06
Overseas born	57 (8.8%)	8107 (12.0%)	0.11	57 (8.8%)	466 (7.2%)	0.06
**Maternal relationship status**						
With a partner	347 (53.6%)	35666 (52.9%)	0.01	347 (53.6%)	3474 (53.8%)	0.00
Without a partner	294 (45.4%)	31241 (46.4%)	0.02	294 (45.4%)	2931 (45.4%)	0.00
Missing or unknown	6 (1.0%)	472 (0.7%)	0.03	6 (1.0%)	50 (0.8%)	0.01
**Socio-economic deprivation quintile of maternal residence**						
Quintile 1 (Least disadvantaged)	222 (34.3%)	25550 (37.9%)	0.08	222 (34.3%)	2289 (35.5%)	0.02
Quintile 2	212 (32.8%)	20962 (31.1%)	0.04	212 (32.8%)	2129 (33.0%)	0.00
Quintile 3	103 (15.9%)	11352 (16.8%)	0.03	103 (15.9%)	997 (15.4%)	0.01
Quintile 4	61 (9.4%)	5312 (7.9%)	0.05	61 (9.4%)	587 (9.1%)	0.01
Quintile 5 (Most disadvantaged)	47 (7.3%)	3837 (5.7%)	0.06	47 (7.3%)	426 (6.6%)	0.03
**Remoteness of maternal residence**						
Major cities	355 (54.9%)	38783 (57.6%)	0.05	355 (54.9%)	3515 (54.5%)	0.01
Inner regional	219 (33.8%)	19373 (28.8%)	0.11	219 (33.8%)	2274 (35.2%)	0.03
Outer regional, remote, very remote	71 (11.0%)	8866 (13.2%)	0.07	71 (11.0%)	639 (9.9%)	0.04
**Parity**						
Nulliparous	210 (32.5%)	23757 (35.3%)	0.06	210 (32.5%)	2077 (32.2%)	0.01
One	204 (31.5%)	17673 (26.2%)	0.12	204 (31.5%)	2090 (32.4%)	0.02
Two or more	233 (36.0%)	25936 (38.5%)	0.05	233 (36.0%)	2288 (35.4%)	0.01
**A previous child having a major congenital malformation**						
Nulliparous	210 (32.5%)	23714 (35.2%)	0.06	210 (32.5%)	2077 (32.2%)	0.01
Yes	23 (3.6%)	2502 (3.7%)	0.01	23 (3.6%)	218 (3.4%)	0.01
No	414 (64.0%)	41163 (61.1%)	0.06	414 (64.0%)	4160 (64.4%)	0.01
**Maternal hospitalisation in 12 months prior to date of conception**						
None	514 (79.4%)	55399 (82.2%)	0.07	514 (79.4%)	5256 (81.4%)	0.05
One	92 (14.2%)	8847 (13.1%)	0.03	92 (14.2%)	843 (13.1%)	0.03
Two or more	41 (6.3%)	3133 (4.6%)	0.07	41 (6.3%)	356 (5.5%)	0.03
**Maternal morbidity**						

© 2025 Tran DT et al. *JAMA Internal Medicine*.

Characteristics	Before matching			After matching		
	Exposed to transdermal nicotine patches	Unexposed*	Diff †	Exposed to transdermal nicotine patches	Unexposed*	Diff †
**Total number**	**647**	**67379**		**647**	**6455**	
Mental health disorder	211 (32.6%)	14032 (20.8%)	0.27	211 (32.6%)	2036 (31.5%)	0.02
Chronic airway disorder	115 (17.8%)	8532 (12.7%)	0.14	115 (17.8%)	1128 (17.5%)	0.01
Gastro-oesophageal reflux	58 (9.0%)	3270 (4.9%)	0.16	58 (9.0%)	536 (8.3%)	0.02
Use of non-steroid anti-inflammation drugs	64 (9.9%)	4476 (6.6%)	0.12	64 (9.9%)	649 (10.1%)	0.01
Use of steroids	37 (5.7%)	2779 (4.1%)	0.07	37 (5.7%)	381 (5.9%)	0.01
Anaemia and coagulation disorders	35 (5.4%)	3102 (4.6%)	0.04	35 (5.4%)	369 (5.7%)	0.01
Drug or alcohol disorders	39 (6.0%)	4023 (6.0%)	0.00	39 (6.0%)	355 (5.5%)	0.02
Thyroid disorder	14 (2.2%)	622 (0.9%)	0.10	14 (2.2%)	143 (2.2%)	0.00
Cardiovascular disease	25 (3.9%)	1209 (1.8%)	0.13	25 (3.9%)	219 (3.4%)	0.03
Pre-existing diabetes	9 (1.4%)	574 (0.9%)	0.05	9 (1.4%)	80 (1.2%)	0.01
Pre-existing hypertension	13 (2.0%)	623 (0.9%)	0.09	13 (2.0%)	110 (1.7%)	0.02
Epilepsy	≈6 (≈0.9%)	467 (0.7%)	0.03	≈6 (≈0.9%)	28 (0.4%)	0.06
Chronic renal disease	≈6 (≈0.9%)	549 (0.8%)	0.01	≈6 (≈0.9%)	34 (0.5%)	0.05
Rheumatic disease	≈6 (≈0.9%)	8 (0.01%)	0.02	≈6 (≈0.9%)	≈6 (≈0.1%)	0.01

Data are the number (percentages) and † absolute standardised differences.

*: Unexposed infants were born to women who smoked in the first trimester and were not dispensed a prescribed smoking cessation pharmacotherapy during 90 days before conception and the first trimester. In the matched sample, exposed infants were matched to unexposed infants (1:10) on propensity score and year of conception.

‡: Australian Aboriginal and/or Torres Strait Islander background.

≈: For data privacy, cell value <6 is not shown, and was replaced with a 6; percentage was adjusted accordingly.

© 2025 Tran DT et al. *JAMA Internal Medicine*.

eTable 14: Characteristics of infants exposed to varenicline and unexposed infants in New South Wales cohort, Australia, before and after propensity score matching

Characteristics	Before matching			After matching		
	Exposed to varenicline	Unexposed*	Diff †	Exposed to varenicline	Unexposed*	Diff †
**Total number**	**1519**	**87593**		**1519**	**15120**	
**Year of conception**						
2008	143 (9.4%)	20214 (23.1%)	0.38	143 (9.4%)	1429 (9.5%)	0.00
2009	214 (14.1%)	9317 (10.6%)	0.11	214 (14.1%)	2123 (14.0%)	0.00
2010	256 (16.9%)	8491 (9.7%)	0.21	256 (16.9%)	2523 (16.7%)	0.00
2011	187 (12.3%)	8022 (9.2%)	0.10	187 (12.3%)	1870 (12.4%)	0.00
2012	154 (10.1%)	7257 (8.3%)	0.06	154 (10.1%)	1540 (10.2%)	0.00
2013	122 (8.0%)	7003 (8.0%)	0.00	122 (8.0%)	1210 (8.0%)	0.00
2014	110 (7.2%)	6705 (7.7%)	0.02	110 (7.2%)	1097 (7.3%)	0.00
2015	94 (6.2%)	6160 (7.0%)	0.03	94 (6.2%)	940 (6.2%)	0.00
2016	102 (6.7%)	6274 (7.2%)	0.02	102 (6.7%)	1020 (6.7%)	0.00
2017	107 (7.0%)	6548 (7.5%)	0.02	107 (7.0%)	1068 (7.1%)	0.00
2018	30 (2.0%)	1602 (1.8%)	0.01	30 (2.0%)	300 (2.0%)	0.00
**Baby's sex**						
Male	813 (53.5%)	45011 (51.4%)	0.04	813 (53.5%)	8166 (54.0%)	0.01
Female	706 (46.5%)	42573 (48.6%)	0.04	706 (46.5%)	6954 (46.0%)	0.01
**Maternal age at childbirth (year)**						
Under 25	358 (23.6%)	31445 (35.9%)	0.27	358 (23.6%)	3560 (23.5%)	0.00
25-29	459 (30.2%)	24522 (28.0%)	0.05	459 (30.2%)	4699 (31.1%)	0.02
30-34	390 (25.7%)	18628 (21.3%)	0.10	390 (25.7%)	3832 (25.3%)	0.01
35 and older	312 (20.5%)	12997 (14.8%)	0.15	312 (20.5%)	3029 (20.0%)	0.01
**Maternal Indigenous status** ‡						
Yes	168 (11.1%)	16593 (18.9%)	0.22	168 (11.1%)	1659 (11.0%)	0.00
No	1350 (88.9%)	70964 (81.0%)	0.22	1350 (88.9%)	13450 (89.0%)	0.00
**Maternal country of birth**						
Born in New Zealand	1302 (85.7%)	76791 (87.7%)	0.06	1302 (85.7%)	13088 (86.6%)	0.02
Born in another country	217 (14.3%)	10783 (12.3%)	0.06	217 (14.3%)	2032 (13.4%)	0.02
**Relationship status**						
With a partner	1010 (66.5%)	47142 (53.8%)	0.26	1010 (66.5%)	10292 (68.1%)	0.03
Without a partner	500 (32.9%)	39744 (45.4%)	0.26	500 (32.9%)	4758 (31.5%)	0.03
Missing or unknown	9 (0.6%)	707 (0.8%)	0.1	9 (0.6%)	70 (0.4%)	0.02
**Socio-economic deprivation quintile of maternal residence**						
Quintile 1 (Least disadvantaged)	455 (30.0%)	32996 (37.7%)	0.16	455 (30.0%)	4370 (28.9%)	0.02
Quintile 2	458 (30.2%)	27285 (31.1%)	0.02	458 (30.2%)	4663 (30.8%)	0.01
Quintile 3	291 (19.2%)	14790 (16.9%)	0.06	291 (19.2%)	2849 (18.8%)	0.01
Quintile 4	174 (11.5%)	7010 (8.0%)	0.12	174 (11.5%)	1839 (12.2%)	0.02
Quintile 5 (Most disadvantaged)	137 (9.0%)	5103 (5.8%)	0.12	137 (9.0%)	1354 (9.0%)	0.00
**Remoteness of maternal residence**						
Major cities	934 (61.5%)	50723 (57.9%)	0.07	934 (61.5%)	9436 (62.4%)	0.02
Inner regional	434 (28.6%)	25096 (28.7%)	0.00	434 (28.6%)	4206 (27.8%)	0.02
Outer regional, remote, very remote	147 (9.7%)	11377 (13.0%)	0.10	147 (9.7%)	1433 (9.5%)	0.01
**Parity**						
Nulliparous	513 (33.8%)	31425 (35.9%)	0.04	513 (33.8%)	5197 (34.4%)	0.01
One	451 (29.7%)	22905 (26.1%)	0.08	451 (29.7%)	4380 (29.0%)	0.02
Two or more	555 (36.5%)	33247 (38.0%)	0.03	555 (36.5%)	5543 (36.7%)	0.00
**A previous child having a major congenital malformation**						
Nulliparous	513 (33.8%)	31352 (35.8%)	0.04	513 (33.8%)	5197 (34.4%)	0.01
Yes	48 (3.2%)	3024 (3.5%)	0.02	48 (3.2%)	435 (2.9%)	0.02
No	958 (63.1%)	53217 (60.8%)	0.05	958 (63.1%)	9488 (62.8%)	0.01
**Maternal hospitalisation in 12 months prior to date of conception**						
None	1220 (80.3%)	72000 (82.2%)	0.05	1220 (80.3%)	12354 (81.7%)	0.04
One	230 (15.1%)	11571 (13.2%)	0.06	230 (15.1%)	2108 (13.9%)	0.03
Two or more	69 (4.5%)	4022 (4.6%)	0.00	69 (4.5%)	658 (4.4%)	0.01
**Maternal morbidity**						
Mental health disorder	310 (20.4%)	17393 (19.9%)	0.01	310 (20.4%)	2860 (18.9%)	0.04
Chronic airway disorder	244 (16.1%)	10748 (12.3%)	0.11	244 (16.1%)	2259 (14.9%)	0.03
Gastro-oesophageal reflux	125 (8.2%)	4016 (4.6%)	0.15	125 (8.2%)	1172 (7.8%)	0.02

© 2025 Tran DT et al. *JAMA Internal Medicine*.

Characteristics	Before matching			After matching		
	Exposed to varenicline	Unexposed*	Diff †	Exposed to varenicline	Unexposed*	Diff †
**Total number**	**1519**	**87593**		**1519**	**15120**	
Use of non-steroid anti-inflammation drugs	142 (9.3%)	5533 (6.3%)	0.11	142 (9.3%)	1259 (8.3%)	0.04
Use of steroids	84 (5.5%)	3166 (3.6%)	0.09	84 (5.5%)	799 (5.3%)	0.01
Anaemia and coagulation disorders	54 (3.6%)	3659 (4.2%)	0.03	54 (3.6%)	461 (3.0%)	0.03
Drug or alcohol disorders	33 (2.2%)	5289 (6.0%)	0.20	33 (2.2%)	313 (2.1%)	0.01
Thyroid disorder	21 (1.4%)	728 (0.8%)	0.05	21 (1.4%)	179 (1.2%)	0.02
Cardiovascular disease	38 (2.5%)	1417 (1.6%)	0.06	38 (2.5%)	345 (2.3%)	0.01
Pre-existing diabetes	20 (1.3%)	716 (0.8%)	0.05	20 (1.3%)	163 (1.1%)	0.02
Pre-existing hypertension	27 (1.8%)	827 (0.9%)	0.07	27 (1.8%)	236 (1.6%)	0.02
Epilepsy	10 (0.7%)	611 (0.7%)	0.00	10 (0.7%)	108 (0.7%)	0.01
Chronic renal disease	10 (0.7%)	706 (0.8%)	0.02	10 (0.7%)	81 (0.5%)	0.02
Rheumatic disease	0 (0.0%)	10 (0.01%)	0.00	0 (0.0%)	≈6 (0.04%)	0.00

Data are the number (percentages) and † absolute standardised differences.

*: Unexposed infants were born to women who smoked in the first trimester and were not dispensed a prescribed smoking cessation pharmacotherapy during 90 days before conception and the first trimester. In the matched sample, exposed infants were matched to unexposed infants (1:10) on propensity score and year of conception.

‡: Australian Aboriginal and/or Torres Strait Islander background.

≈: For data privacy, cell value <6 is not shown, and was replaced with a 6; percentage was adjusted accordingly.

© 2025 Tran DT et al. *JAMA Internal Medicine*.

eTable 15: Characteristics of infants exposed to bupropion and unexposed infants in New South Wales cohort, Australia, before and after propensity score matching

Characteristics	Before matching			After matching		
	Exposed to bupropion	Unexposed*	Diff †	Exposed to bupropion	Unexposed*	Diff †
**Total number**	**293**	**136636**		**293**	**2908**	
**Year of conception**						
2002	25 (8.5%)	5864 (4.3%)	0.17	25 (8.5%)	250 (8.6%)	0.00
2003	52 (17.7%)	10981 (8.0%)	0.29	52 (17.7%)	516 (17.7%)	0.00
2004	32 (10.9%)	10966 (8.0%)	0.10	32 (10.9%)	320 (11.0%)	0.00
2005	48 (16.4%)	10701 (7.8%)	0.26	48 (16.4%)	480 (16.5%)	0.00
2006	38 (13.0%)	10531 (7.7%)	0.17	38 (13.0%)	379 (13.0%)	0.00
2007	33 (11.3%)	10169 (7.4%)	0.13	33 (11.3%)	330 (11.3%)	0.00
2008	17 (5.8%)	10045 (7.4%)	0.06	17 (5.8%)	170 (5.8%)	0.00
2009	7 (2.4%)	9317 (6.8%)	0.21	7 (2.4%)	70 (2.4%)	0.00
2010	7 (2.4%)	8491 (6.2%)	0.19	7 (2.4%)	70 (2.4%)	0.00
2011						
2012 §	9 (3.1%)	15279 (11.2%)	0.32	9 (3.1%)	82 (2.8%)	0.01
2013						
2014 §	8 (2.7%)	13708 (10.0%)	0.30	8 (2.7%)	80 (2.8%)	0.00
2015						
2016 §	8 (2.7%)	12434 (9.1%)	0.27	8 (2.7%)	80 (2.8%)	0.00
2017						
2018 §	9 (3.1%)	8150 (6.0%)	0.14	9 (3.1%)	81 (2.8%)	0.02
**Baby's sex**						
Male	151 (51.5%)	70266 (51.4%)	0.00	151 (51.5%)	1471 (50.6%)	0.02
Female	142 (48.5%)	66345 (48.6%)	0.00	142 (48.5%)	1437 (49.4%)	0.02
**Maternal age at childbirth (year)**						
Under 25	63 (21.5%)	49762 (36.4%)	0.33	63 (21.5%)	587 (20.2%)	0.03
25-29	87 (29.7%)	37691 (27.6%)	0.05	87 (29.7%)	926 (31.8%)	0.05
30-34	85 (29.0%)	29487 (21.6%)	0.17	85 (29.0%)	874 (30.1%)	0.02
35 and older	58 (19.8%)	19693 (14.4%)	0.14	58 (19.8%)	521 (17.9%)	0.05
**Maternal Indigenous status** ‡						
Yes	19 (6.5%)	24054 (17.6%)	0.35	19 (6.5%)	144 (5.0%)	0.07
No	273 (93.2%)	112531 (82.4%)	0.33	273 (93.2%)	2753 (94.7%)	0.06
**Maternal country of birth**						
Born in Australia	255 (87.0%)	120254 (88.0%)	0.03	255 (87.0%)	2569 (88.3%)	0.04
Born in another country	38 (13.0%)	16349 (12.0%)	0.03	38 (13.0%)	339 (11.7%)	0.04
**Maternal relationship status**						
With a partner	201 (68.6%)	73538 (53.8%)	0.31	201 (68.6%)	2076 (71.4%)	0.06
Without a partner	83 (28.3%)	61651 (45.1%)	0.35	83 (28.3%)	733 (25.2%)	0.07
Missing or unknown	9 (3.1%)	1447 (1.1%)	0.21	9 (3.1%)	99 (3.4%)	0.02
**Socio-economic deprivation quintile of maternal residence**						
Quintile 1 (Least disadvantaged)	85 (29.0%)	49601 (36.3%)	0.16	85 (29.0%)	730 (25.1%)	0.09
Quintile 2	84 (28.7%)	42923 (31.4%)	0.06	84 (28.7%)	849 (29.2%)	0.01
Quintile 3	51 (17.4%)	23653 (17.3%)	0.00	51 (17.4%)	529 (18.2%)	0.02
Quintile 4	38 (13.0%)	11362 (8.3%)	0.15	38 (13.0%)	446 (15.3%)	0.07
Quintile 5 (Most disadvantaged)	35 (11.9%)	8640 (6.3%)	0.20	35 (11.9%)	354 (12.2%)	0.01
**Remoteness of maternal residence**						
Major cities	177 (60.4%)	78964 (57.8%)	0.05	177 (60.4%)	1809 (62.2%)	0.04
Inner regional	80 (27.3%)	39205 (28.7%)	0.03	80 (27.3%)	748 (25.7%)	0.04
Outer regional, remote, very remote	36 (12.3%)	18030 (13.2%)	0.03	36 (12.3%)	351 (12.1%)	0.01
**Parity**						
Nulliparous	95 (32.4%)	48863 (35.8%)	0.07	95 (32.4%)	981 (33.7%)	0.03
One	88 (30.0%)	36927 (27.0%)	0.07	88 (30.0%)	855 (29.4%)	0.01
Two or more	110 (37.5%)	50816 (37.2%)	0.01	110 (37.5%)	1072 (36.9%)	0.01
**A previous child having a major congenital malformation**						
Nulliparous	95 (32.4%)	48781 (35.7%)	0.07	95 (32.4%)	981 (33.7%)	0.03
Yes	9 (3.1%)	3733 (2.7%)	0.02	9 (3.1%)	81 (2.8%)	0.02
No	189 (64.5%)	84122 (61.6%)	0.06	189 (64.5%)	1846 (63.5%)	0.02
**Maternal hospitalisation in 12 months prior to date of conception**						
None	231 (78.8%)	111856 (81.9%)	0.08	231 (78.8%)	2324 (79.9%)	0.03

© 2025 Tran DT et al. *JAMA Internal Medicine*.

Characteristics	Before matching			After matching		
	Exposed to bupropion	Unexposed*	Diff †	Exposed to bupropion	Unexposed*	Diff †
**Total number**	**293**	**136636**		**293**	**2908**	
One	46 (15.7%)	18509 (13.5%)	0.06	46 (15.7%)	427 (14.7%)	0.03
Two or more	16 (5.5%)	6271 (4.6%)	0.04	16 (5.5%)	157 (5.4%)	0.00
**Maternal morbidity**						
Mental health disorder	67 (22.9%)	27029 (19.8%)	0.08	67 (22.9%)	595 (20.5%)	0.06
Chronic airway disorder	68 (23.2%)	16644 (12.2%)	0.29	68 (23.2%)	646 (22.2%)	0.02
Gastro-oesophageal reflux	32 (10.9%)	5563 (4.1%)	0.26	32 (10.9%)	258 (8.9%)	0.07
Use of non-steroid anti-inflammation drugs	43 (14.7%)	8508 (6.2%)	0.28	43 (14.7%)	338 (11.6%)	0.09
Use of steroids	23 (7.8%)	4013 (2.9%)	0.22	23 (7.8%)	177 (6.1%)	0.07
Anaemia and coagulation disorders	14 (4.8%)	5186 (3.8%)	0.05	14 (4.8%)	105 (3.6%)	0.06
Drug or alcohol disorders	≈6 (2.0%)	9279 (6.8%)	0.23	≈6 (2.0%)	52 (1.8%)	0.02
Thyroid disorder	≈6 (2.0%)	1067 (0.8%)	0.11	≈6 (2.0%)	31 (1.1%)	0.08
Cardiovascular disease	9 (3.1%)	1968 (1.4%)	0.11	9 (3.1%)	67 (2.3%)	0.05
Pre-existing diabetes	≈6 (2.0%)	982 (0.7%)	0.11	≈6 (2.0%)	21 (0.7%)	0.11
Pre-existing hypertension	≈6 (2.0%)	1279 (0.9%)	0.09	≈6 (2.0%)	42 (1.4%)	0.05
Epilepsy	0 (0.0%)	946 (0.7%)	0.12	0 (0.0%)	≈6 (0.2%)	0.03
Chronic renal disease	≈6 (2.0%)	1049 (0.8%)	0.11	≈6 (2.0%)	31 (1.1%)	0.08
Rheumatic disease	0 (0.0%)	≈6 (0.0%)	0.00	0 (0.0%)	≈6 (0.2%)	0.04

Data are the number (percentages) and † absolute standardised differences.

*: Unexposed infants were born to women who smoked in the first trimester and were not dispensed a prescribed smoking cessation pharmacotherapy during 90 days before conception and the first trimester. In the matched sample, exposed infants were matched to unexposed infants (1:10) on propensity score and year of conception.

§: For data privacy, small counts are not shown, results for 2011 and 2012 were combined, results for 2013 and 2014 were combined, results for 2015 and 2016 were combined, results for 2017 and 2018 were combined.

‡: Australian Aboriginal and/or Torres Strait Islander background.

≈: For data privacy, cell value <6 is not shown, and was replaced with a 6; percentage was adjusted accordingly.

© 2025 Tran DT et al. *JAMA Internal Medicine*.

5. eAppendix 5: Characteristics of infants in Norway and Sweden cohort, before and after propensity score matching

eTable 16: Characteristics of infants exposed to varenicline and unexposed infants in Norway and Sweden cohort, before and after propensity score matching

Characteristics	Before matching			After matching		
	Exposed to varenicline	Unexposed*	Diff †	Exposed to varenicline	Unexposed*	Diff †
**Total number**	**558**	**97885**		**558**	**5580**	
**Year of childbirth**						
2007	≈6 (1.1%)	4981 (5.1%)		≈6 (1.1%)	≈60 (1.1%)	0.00
2008	44 (7.9%)	6554 (6.7%)	0.05	44 (7.9%)	440 (7.9%)	0.00
2009	44 (7.9%)	8003 (8.2%)	0.01	44 (7.9%)	440 (7.9%)	0.00
2010	56 (10.0%)	12017 (12.3%)	0.07	56 (10.0%)	560 (10.0%)	0.00
2011	68 (12.2%)	11073 (11.3%)	0.03	68 (12.2%)	680 (12.2%)	0.00
2012	80 (14.3%)	10014 (10.2%)	0.13	80 (14.3%)	800 (14.3%)	0.00
2013	65 (11.6%)	9111 (9.3%)	0.08	65 (11.6%)	650 (11.6%)	0.00
2014	47 (8.4%)	8783 (9.0%)	0.02	47 (8.4%)	470 (8.4%)	0.00
2015	38 (6.8%)	7700 (7.9%)	0.04	38 (6.8%)	380 (6.8%)	0.00
2016	23 (4.1%)	7069 (7.2%)	0.13	23 (4.1%)	230 (4.1%)	0.00
2017	43 (7.7%)	6002 (6.1%)	0.06	43 (7.7%)	430 (7.7%)	0.00
2018	39 (7.0%)	5437 (5.6%)	0.06	39 (7.0%)	390 (7.0%)	0.00
2019	≈5 (0.9%)	1141 (1.2%)	0.03	≈5 (0.9%)	≈50 (0.9%)	0.00
**Baby's sex**						
Male	281 (50.4%)	50523 (51.6%)	0.03	281 (50.4%)	2934 (52.6%)	0.04
Female	277 (49.6%)	47362 (48.4%)	0.03	277 (49.6%)	2646 (47.4%)	0.04
**Maternal age at childbirth (year)**						
Under 25	62 (11.1%)	29886 (30.5%)	0.49	62 (11.1%)	605 (10.8%)	0.01
25-29	150 (26.9%)	30485 (31.1%)	0.09	150 (26.9%)	1466 (26.3%)	0.01
30-34	197 (35.3%)	22166 (22.6%)	0.28	197 (35.3%)	2018 (36.2%)	0.02
35 and older	149 (26.7%)	15348 (15.7%)	0.27	149 (26.7%)	1491 (26.7%)	0.00
**Maternal country of birth**						
Native born	451 (80.8%)	76849 (78.5%)	0.06	451 (80.8%)	4567 (81.8%)	0.03
Overseas born	106 (19.0%)	20792 (21.2%)	0.06	106 (19.0%)	1004 (18.0%)	0.03
**Relationship status**						
With a partner	87 (15.6%)	19324 (19.7%)	0.11	87 (15.6%)	810 (14.5%)	0.03
Without a partner	467 (83.7%)	78145 (79.8%)	0.10	467 (83.7%)	4731 (84.8%)	0.03
**Education**						
Compulsory education	144 (25.8%)	37711 (38.5%)	0.27	144 (25.8%)	1428 (25.6%)	0.00
Secondary education	303 (54.3%)	48500 (49.5%)	0.10	303 (54.3%)	3041 (54.5%)	0.00
Post-secondary education	106 (19.0%)	8768 (9.0%)	0.29	106 (19.0%)	1087 (19.5%)	0.01
**Maternal body mass index (BMI)**						
Underweight (BMI <18.5)	11 (2.0%)	3379 (3.5%)	0.09	11 (2.0%)	111 (2.0%)	0.00
Normal (BMI 18.5 to <25)	207 (37.1%)	41487 (42.4%)	0.11	207 (37.1%)	2111 (37.8%)	0.02
Overweight (BMI 25 to <30)	128 (22.9%)	22511 (23.0%)	0.00	128 (22.9%)	1219 (21.8%)	0.03
Obese (BMI >=30)	107 (19.2%)	16661 (17.0%)	0.06	107 (19.2%)	1015 (18.2%)	0.03
**Parity**						
Nulliparous	227 (40.7%)	43256 (44.2%)	0.07	227 (40.7%)	2238 (40.1%)	0.01
One	183 (32.8%)	30358 (31.0%)	0.04	183 (32.8%)	1866 (33.4%)	0.01
Two or more	148 (26.5%)	24271 (24.8%)	0.04	148 (26.5%)	1476 (26.5%)	0.00
**A previous child having a major congenital malformation**						
Nulliparous	227 (40.7%)	43256 (44.2%)	0.07	227 (40.7%)	2238 (40.1%)	0.01
Yes	6 (1.1%)	1256 (1.3%)	0.02	6 (1.1%)	70 (1.3%)	0.02
No	325 (58.2%)	53373 (54.5%)	0.08	325 (58.2%)	3272 (58.6%)	0.01
**Maternal hospitalisation in 12 months prior to date of conception**						
None	457 (81.9%)	80143 (81.9%)	0.00	457 (81.9%)	4629 (83.0%)	0.03
One	73 (13.1%)	13744 (14.0%)	0.03	73 (13.1%)	676 (12.1%)	0.03
Two or more	28 (5.0%)	3998 (4.1%)	0.04	28 (5.0%)	275 (4.9%)	0.00

© 2025 Tran DT et al. *JAMA Internal Medicine*.

Characteristics	Before matching			After matching		
	Exposed to varenicline	Unexposed*	Diff †	Exposed to varenicline	Unexposed*	Diff †
**Total number**	**558**	**97885**		**558**	**5580**	
**Maternal morbidity**						
Mental health disorder	151 (27.1%)	20948 (21.4%)	0.13	151 (27.1%)	1461 (26.2%)	0.02
Chronic airway disorder	90 (16.1%)	8348 (8.5%)	0.23	90 (16.1%)	827 (14.8%)	0.04
Gastro-oesophageal reflux	68 (12.2%)	6102 (6.2%)	0.21	68 (12.2%)	638 (11.4%)	0.02
Use of non-steroid anti-inflammation drugs	148 (26.5%)	17311 (17.7%)	0.21	148 (26.5%)	1399 (25.1%)	0.03
Use of steroids	38 (6.8%)	3066 (3.1%)	0.17	38 (6.8%)	346 (6.2%)	0.02
Anaemia and coagulation disorders	19 (3.4%)	2402 (2.5%)	0.06	19 (3.4%)	181 (3.2%)	0.01
Drug or alcohol disorders	24 (4.3%)	4013 (4.1%)	0.01	24 (4.3%)	222 (4.0%)	0.02
Thyroid disorder	35 (6.3%)	2813 (2.9%)	0.16	35 (6.3%)	325 (5.8%)	0.02
Cardiovascular disease	32 (5.7%)	2108 (2.2%)	0.18	32 (5.7%)	278 (5.0%)	0.03
Pre-existing diabetes	12 (2.2%)	1266 (1.3%)	0.07	12 (2.2%)	97 (1.7%)	0.03
Pre-existing hypertension	10 (1.8%)	834 (0.9%)	0.08	10 (1.8%)	83 (1.5%)	0.02
Epilepsy	5 (0.9%)	955 (1.0%)	0.01	5 (0.9%)	33 (0.6%)	0.04
Chronic renal disease	≈5 (0.9%)	625 (0.6%)	0.03	≈5 (0.9%)	≈ 50 (0.9%)	0.01
Rheumatic disease	≈5 (0.9%)	290 (0.3%)	0.08	≈5 (0.9%)	Suppressed	<0.1

Data are the number (percentages) and † absolute standardised differences.

*: Unexposed infants were born to women who smoked in the first trimester and were not dispensed a prescribed smoking cessation pharmacotherapy during 90 days before conception and the first trimester. In the matched sample, exposed infants were matched to unexposed infants (1:10) on propensity score and year of childbirth.

≈: For data privacy, cell value <5 is not shown, and was replaced with a 5; percentage was adjusted accordingly.

© 2025 Tran DT et al. *JAMA Internal Medicine*.

6. eAppendix 6: Major congenital malformations, overall and subgroups: unadjusted and adjusted results

eTable 17: Major congenital malformations (overall and subgroups) in liveborn infants exposed to nicotine replacement therapy, varenicline, or bupropion in the first trimester and unexposed infants, before propensity score matching: country-specific number, prevalence and unadjusted relative risk.

Overall and subgroups	Country	Exposed *			Unexposed *			Unadjusted relative risk (95% CI)
		number	denominator	prevalence per 1000 livebirths (95%CI)	number	denominator	Prevalence per 1000 livebirths (95%CI)	
**Nicotine replacement therapy (any formulation)**								
Overall	New Zealand	345	9242	37.3 (33.5-41.5)	3041	89659	33.9 (32.7-35.1)	1.10 (0.98-1.23)
	New South Wales	26	647	40.2 (26.3-58.9)	2552	67379	37.9 (36.4-39.4)	1.06 (0.73-1.55)
Congenital heart defects	New Zealand	80	9242	8.7 (6.9-10.8)	765	89659	8.5 (7.9-9.2)	1.01 (0.81-1.28)
	New South Wales	11	647	17.0 (8.5-30.4)	492	67379	7.3 (6.7-8.0)	2.33 (1.29-4.21)
Limb anomalies	New Zealand	76	9242	8.2 (6.5-10.3)	647	89659	7.2 (6.7-7.8)	1.14 (0.90-1.45)
	New South Wales	6	647	9.3 (3.4-20.2)	561	67379	8.3 (7.7-9.0)	1.11 (0.50-2.48)
Genital organs	New Zealand	57	9242	6.2 (4.7-8.0)	524	89659	5.8 (5.4-6.4)	1.06 (0.80-1.39)
	New South Wales	<6	647	(supp)	415	67379	6.2 (5.6-6.8)	-
Kidney and urinary tract	New Zealand	43	9242	4.7 (3.4-6.3)	322	89659	3.6 (3.2-4.0)	1.30 (0.94-1.78)
	New South Wales	<6	647	(supp)	309	67379	4.6 (4.1-5.1)	-
Digestive system	New Zealand	36	9242	3.9 (2.7-5.4)	219	89659	2.4 (2.1-2.8)	1.59 (1.12-2.27)
	New South Wales	<6	647	(supp)	290	67379	4.3 (3.8-4.8)	-
Respiratory system	New Zealand	18	9242	2.0 (1.2-3.1)	160	89659	1.8 (1.5-2.1)	1.09 (0.67-1.78)
	New South Wales	<6	647	(supp)	107	67379	1.6 (1.3-1.9)	-
Nervous system	New Zealand	10	9242	1.1 (0.5-2.0)	172	89659	1.9 (1.6-2.2)	0.56 (0.30-1.07)
	New South Wales	<6	647	(supp)	167	67379	2.5 (2.1-2.9)	-
Oro-facial clefts	New Zealand	17	9242	1.8 (1.1-3.0)	185	89659	2.1 (1.8-2.4)	0.89 (0.54-1.47)
	New South Wales	0	647	-	106	67379	1.6 (1.3-1.9)	-
Abdominal wall	New Zealand	11	9242	1.2 (0.6-2.1)	100	89659	1.1 (0.9-1.4)	1.07 (0.57-1.99)
	New South Wales	<6	647	(supp)	55	67379	0.8 (0.6-1.1)	-
Eye	New Zealand	7	9242	0.8 (0.3-1.6)	38	89659	0.4 (0.3-0.6)	1.79 (0.80-4.00)
	New South Wales	0	647	-	32	67379	0.5 (0.3-0.7)	-
Ear	New Zealand	<3	9242	(supp)	11	89659	0.1 (0.1-0.2)	0.88 (0.11-6.83)
	New South Wales	0	647	-	16	67379	0.2 (0.1-0.4)	-
Others	New Zealand	33	9242	3.6 (2.5-5.0)	286	89659	3.2 (2.8-3.6)	1.12 (0.78-1.61)
	New South Wales	<6	647	(supp)	318	67379	4.7 (4.2-5.3)	-
**Varenicline**								
Overall	New Zealand	30	955	31.4 (21.2-44.8)	2263	65164	34.7 (33.3-36.2)	0.90 (0.63-1.30)
	New South Wales	49	1519	32.3 (23.9-42.7)	3298	87593	37.7 (36.4-39.0)	0.86 (0.65-1.13)

© 2025 Tran DT et al. *JAMA Internal Medicine*.

Overall and subgroups	Country	Exposed *			Unexposed *			Unadjusted relative risk (95% CI)
		number	denominator	prevalence per 1000 livebirths (95%CI)	number	denominator	Prevalence per 1000 livebirths (95%CI)	
Congenital heart defects	Norway & Sweden	20	558	35.8 (21.9-55.4)	3416	97885	34.9 (33.7-36.1)	1.03 (0.66-1.59)
	New Zealand	8	955	8.4 (3.6-16.5)	595	65164	9.1 (8.4-9.9)	0.92 (0.46-1.84)
	New South Wales	7	1519	4.6 (1.9-9.5)	643	87593	7.3 (6.8-7.9)	0.63 (0.30-1.32)
Limb anomalies	Norway & Sweden	12	558	21.5 (11.1-37.6)	1437	97885	14.7 (13.9-15.5)	1.46 (0.83-2.59)
	New Zealand	6	955	6.3 (2.3-13.7)	477	65164	7.3 (6.7-8.0)	0.86 (0.38-1.92)
	New South Wales	9	1519	5.9 (2.7-11.3)	735	87593	8.4 (7.8-9.0)	0.71 (0.37-1.36)
Genital organs	Norway & Sweden	<5	558	(supp)	529	97885	5.4 (5.0-5.9)	-
	New Zealand	3	955	3.1 (0.7-9.2)	372	65164	5.7 (5.1-6.3)	0.55 (0.18-1.71)
	New South Wales	14	1519	9.2 (5.0-15.5)	542	87593	6.2 (5.7-6.7)	1.49 (0.88-2.53)
Kidney and urinary tract	Norway & Sweden	<5	558	(supp)	328	97885	3.4 (3.0-3.7)	-
	New Zealand	11	955	11.5 (5.8-20.6)	247	65164	3.8 (3.3-4.3)	3.04 (1.66-5.56)
	New South Wales	<6	1519	(supp)	379	87593	4.3 (3.9-4.8)	-
Digestive system	Norway & Sweden	<5	558	(supp)	334	97885	3.4 (3.1-3.8)	-
	New Zealand	0	955	-	171	65164	2.6 (2.3-3.1)	-
	New South Wales	<6	1519	(supp)	397	87593	4.5 (4.1-5.0)	-
Respiratory system	Norway & Sweden	<5	558	(supp)	180	97885	1.8 (1.6-2.1)	-
	New Zealand	<3	955	(supp)	111	65164	1.7 (1.4-2.1)	0.61 (0.09-4.40)
	New South Wales	<6	1519	(supp)	147	87593	1.7 (1.4-2.0)	-
Nervous system	Norway & Sweden	0	558	-	27	97885	0.3 (0.2-0.4)	-
	New Zealand	3	955	3.1 (0.7-9.2)	124	65164	1.9 (1.6-2.3)	1.65 (0.53-5.19)
	New South Wales	<6	1519	(supp)	213	87593	2.4 (2.1-2.8)	-
Oro-facial clefts	Norway & Sweden	0	558	-	116	97885	1.2 (1.0-1.4)	-
	New Zealand	<3	955	(supp)	131	65164	2.0 (1.7-2.4)	0.52 (0.07-3.73)
	New South Wales	<6	1519	(supp)	154	87593	1.8 (1.5-2.1)	-
Abdominal wall	Norway & Sweden	0	558	-	203	97885	2.1 (1.8-2.4)	-
	New Zealand	<3	955	(supp)	71	65164	1.1 (0.9-1.4)	0.96 (0.13-6.92)
	New South Wales	0	1519	-	75	87593	0.9 (0.7-1.1)	-
Eye	Norway & Sweden	0	558	-	53	97885	0.5 (0.4-0.7)	-
	New Zealand	0	955	-	28	65164	0.4 (0.3-0.6)	-
	New South Wales	0	1519	-	51	87593	0.6 (0.4-0.8)	-
Ear	Norway & Sweden	0	558	-	95	97885	1.0 (0.8-1.2)	-
	New Zealand	0	955	-	7	65164	0.1 (0.0-0.2)	-
	New South Wales	0	1519	-	20	87593	0.2 (0.1-0.4)	-
	Norway & Sweden	0	558	-	24	97885	0.3 (0.2-0.4)	-

© 2025 Tran DT et al. *JAMA Internal Medicine*.

Overall and subgroups	Country	Exposed *			Unexposed *			Unadjusted relative risk (95% CI)
		number	denominator	prevalence per 1000 livebirths (95%CI)	number	denominator	Prevalence per 1000 livebirths (95%CI)	
Others	New Zealand	0	955	-	214	65164	3.3 (2.9-3.8)	-
	New South Wales	9	1519	5.9 (2.7-11.3)	416	87593	4.8 (4.3-5.2)	1.25 (0.65-2.41)
	Norway & Sweden	<5	558	(supp)	385	97885	3.9 (3.6-4.4)	-
**Bupropion**								
Overall	New Zealand	26	754	34.5 (22.5-50.5)	2528	73598	34.4 (33.0-35.7)	1.00 (0.68-1.48)
	New South Wales	11	293	37.5 (18.7-67.2)	5048	136636	36.9 (35.9-38.0)	1.02 (0.57-1.82)
Congenital heart defects	New Zealand	5	754	6.6 (2.2-15.5)	650	73598	8.8 (8.2-9.5)	0.75 (0.31-1.81)
	New South Wales	<6	293	(supp)	959	136636	7.0 (6.6-7.5)	-
Limb anomalies	New Zealand	5	754	6.6 (2.2-15.5)	539	73598	7.3 (6.7-8.0)	0.91 (0.38-2.18)
	New South Wales	0	293	-	1140	136636	8.3 (7.9-8.8)	-
Genital organs	New Zealand	7	754	9.3 (3.7-19.1)	422	73598	5.7 (5.2-6.3)	1.62 (0.77-3.42)
	New South Wales	<6	293	(supp)	862	136636	6.3 (5.9-6.7)	-
Kidney and urinary tract	New Zealand	4	754	5.3 (1.5-13.6)	270	73598	3.7 (3.2-4.1)	1.45 (0.54-3.88)
	New South Wales	0	293	-	538	136636	3.9 (3.6-4.3)	-
Digestive system	New Zealand	0	754	-	186	73598	2.5 (2.2-2.9)	-
	New South Wales	<6	293	(supp)	624	136636	4.6 (4.2-4.9)	-
Respiratory system	New Zealand	<3	754	(supp)	133	73598	1.8 (1.5-2.1)	1.47 (0.36-5.93)
	New South Wales	0	293	-	202	136636	1.5 (1.3-1.7)	-
Nervous system	New Zealand	0	754	-	139	73598	1.9 (1.6-2.2)	-
	New South Wales	<6	293	(supp)	317	136636	2.3 (2.1-2.6)	-
Oro-facial clefts	New Zealand	0	754	-	151	73598	2.1 (1.7-2.4)	-
	New South Wales	<6	293	(supp)	225	136636	1.7 (1.4-1.9)	-
Abdominal wall	New Zealand	<3	754	(supp)	79	73598	1.1 (0.9-1.3)	1.24 (0.17-8.88)
	New South Wales	0	293	-	110	136636	0.8 (0.7-1.0)	-
Eye	New Zealand	<3	754	(supp)	33	73598	0.5 (0.3-0.6)	2.96 (0.40-21.63)
	New South Wales	0	293	-	85	136636	0.6 (0.5-0.8)	-
Ear	New Zealand	0	754	-	10	73598	0.1 (0.1-0.3)	-
	New South Wales	0	293	-	33	136636	0.2 (0.2-0.3)	-
Others	New Zealand	3	754	4.0 (0.8-11.6)	239	73598	3.3 (2.9-3.7)	1.23 (0.39-3.83)
	New South Wales	<6	293	(supp)	653	136636	4.8 (4.4-5.2)	-

Data are number with an outcome, denominator, prevalence per 1000 live births (95% CI), and unadjusted relative risk (95% CI).

*: Unexposed infants were born to women who smoked in the first trimester and were not dispensed a prescribed smoking cessation pharmacotherapy during 90 days before conception and the first trimester. For data privacy, data suppression is applied to small counts (<3 for New Zealand, <6 for New South Wales, <5 for Norway and Sweden).

© 2025 Tran DT et al. *JAMA Internal Medicine*.

eTable 18: Major congenital malformations (overall and subgroups) in liveborn infants exposed to nicotine replacement therapy, varenicline, or bupropion in the first trimester and unexposed infants, after propensity score matching: country-specific number, prevalence and adjusted relative risk, and pooled relative risk

Overall and subgroups	Country	Exposed *			Unexposed *			Adjusted relative risk (RR) and 95% CI				
									Meta analysis **			
		number	denominator	prevalence per 1000 livebirths (95%CI)	number	denominator	prevalence per 1000 livebirths (95%CI)	Country-specific	Weight	Pooled RR (95%CI)	P-value from Poisson model	Benjamini-Hochberg correct p value
**Nicotine replacement therapy (any formulation)**												
Overall	New Zealand	325	8678	37.5 (33.5-41.8)	2546	74873	34.0 (32.7-35.4)	1.10 (0.98-1.23)	92.2	1.10 (0.98-1.22)	0.09	0.69
	New South Wales	26	647	40.2 (26.3-58.9)	252	6455	39.0 (34.4-44.2)	1.05 (0.71-1.55)	7.9			
Congenital heart defects	New Zealand	71	8678	8.2 (6.4-10.3)	654	74873	8.7 (8.1-9.4)	0.90 (0.70-1.14)	87.2	0.99 (0.79-1.24)	0.93	0.97
	New South Wales	11	647	17.0 (8.5-30.4)	57	6455	8.8 (6.7-11.4)	1.93 (1.02-3.64)	12.8			
Limb anomalies	New Zealand	72	8678	8.3 (6.5-10.5)	544	74873	7.3 (6.7-7.9)	1.18 (0.92-1.50)	92.1	1.17 (0.93-1.49)	0.18	0.75
	New South Wales	6	647	9.3 (3.4-20.2)	53	6455	8.2 (6.2-10.7)	1.14 (0.49-2.65)	7.9			
Genital organs	New Zealand	57	8678	6.6 (5.0-8.5)	435	74873	5.8 (5.3-6.4)	1.13 (0.86-1.50)	100.0	1.13 (0.86-1.50)	0.38	0.94
	New South Wales	<6	647	(supp)	43	6455	6.7 (4.8-9.0)	-				
Kidney and urinary tract	New Zealand	42	8678	4.8 (3.5-6.5)	270	74873	3.6 (3.2-4.1)	1.31 (0.94-1.82)	100.0	1.31 (0.94-1.82)	0.11	0.69
	New South Wales	<6	647	(supp)	26	6455	4.0 (2.6-5.9)	-				
Digestive system	New Zealand	33	8678	3.8 (2.6-5.3)	187	74873	2.5 (2.2-2.9)	1.53 (1.05-2.23)	100.0	1.53 (1.05-2.23)	0.03	0.41
	New South Wales	<6	647	(supp)	23	6455	3.6 (2.3-5.4)	-				
Respiratory system	New Zealand	17	8678	2.0 (1.1-3.1)	134	74873	1.8 (1.5-2.1)	1.10 (0.67-1.82)	100.0	1.10 (0.67-1.82)	0.71	0.97
	New South Wales	<6	647	(supp)	11	6455	1.7 (0.9-3.1)	-				
Oro-facial clefts	New Zealand	16	8678	1.8 (1.1-3.0)	155	74873	2.1 (1.8-2.4)	0.93 (0.56-1.54)	100.0	0.93 (0.56-1.54)	0.77	0.97
	New South Wales	0	647	-	10	6455	1.6 (0.7-2.9)	-				
Nervous system	New Zealand	10	8678	1.2 (0.6-2.1)	132	74873	1.8 (1.5-2.1)	0.64 (0.33-1.22)	100.0	0.64 (0.33-1.22)	0.17	0.75
	New South Wales	<6	647	(supp)	18	6455	2.8 (1.7-4.4)	-				
Abdominal wall	New Zealand	11	8678	1.3 (0.6-2.3)	83	74873	1.1 (0.9-1.4)	1.27 (0.67-2.41)	100.0	1.27 (0.67-2.41)	0.46	0.94
	New South Wales	<6	647	(supp)	10	6455	1.6 (0.7-2.9)	-				
Eye	New Zealand	7	8678	0.8 (0.3-1.7)	30	74873	0.4 (0.3-0.6)	2.06 (0.92-4.63)	100.0	2.06 (0.92-4.63)	0.08	0.69
	New South Wales	0	647	-	<6	6455	(supp)	-				
Ear	New Zealand	<3	8678	(supp)	8	74873	0.1 (0.1-0.2)	1.08 (0.14-8.60)	100.0	1.08 (0.14-8.60)	0.94	0.97
	New South Wales	0	647	-	0	6455	-	-				
Others	New Zealand	31	8678	3.6 (2.4-5.1)	238	74873	3.2 (2.8-3.6)	1.12 (0.77-1.63)	100.0	1.12 (0.77-1.63)	0.55	0.94
	New South Wales	<6	647	(supp)	34	6455	5.3 (3.7-7.4)	-				
**Varenicline**												
Overall	New Zealand	30	954	31.5 (21.2-44.9)	348	9540	36.5 (32.8-40.5)	0.86 (0.60-1.25)	30.6	0.90 (0.73-1.10)	0.23	0.93
	New South Wales	49	1519	32.3 (23.9-42.7)	569	15120	37.6 (34.6-40.9)	0.86 (0.64-1.14)	49.7			

© 2025 Tran DT et al. *JAMA Internal Medicine*.

Overall and subgroups	Country	Exposed *			Unexposed *			Adjusted relative risk (RR) and 95% CI				
									Meta analysis **			
		number	denominator	prevalence per 1000 livebirths (95%CI)	number	denominator	prevalence per 1000 livebirths (95%CI)	Country-specific	Weight	Pooled RR (95%CI)	P-value from Poisson model	Benjamini-Hochberg correct p value
	Norway & Sweden	20	558	35.8 (21.9-55.4)	189	5580	33.9 (29.2-39.1)	1.06 (0.67-1.67)	19.7			
Congenital heart defects	New Zealand	8	954	8.4 (3.6-16.5)	100	9540	10.5 (8.5-12.8)	0.81 (0.41-1.62)	32.2	0.99 (0.67-1.47)	0.97	0.97
	New South Wales	7	1519	4.6 (1.9-9.5)	123	15120	8.1 (6.8-9.7)	0.56 (0.26-1.20)	26.9			
	Norway & Sweden	12	558	21.5 (11.1-37.6)	71	5580	12.7 (9.9-16.1)	1.69 (0.91-3.13)	40.9			
Limb anomalies	New Zealand	6	954	6.3 (2.3-13.7)	71	9540	7.4 (5.8-9.4)	0.88 (0.40-1.95)	42.1	0.85 (0.51-1.42)	0.53	0.94
	New South Wales	9	1519	5.9 (2.7-11.3)	112	15120	7.4 (6.1-8.9)	0.83 (0.42-1.62)	57.9			
	Norway & Sweden	<5	558	(supp)	33	5580	5.9 (4.1-8.3)	-				
Genital organs	New Zealand	3	954	3.1 (0.7-9.2)	53	9540	5.6 (4.2-7.3)	0.61 (0.20-1.82)	20.2	1.26 (0.77-2.06)	0.36	0.94
	New South Wales	14	1519	9.2 (5.0-15.5)	94	15120	6.2 (5.0-7.6)	1.51 (0.87-2.62)	79.8			
	Norway & Sweden	<5	558	(supp)	17	5580	3.1 (1.8-4.9)	-				
Kidney and urinary tract	New Zealand	11	954	11.5 (5.8-20.6)	40	9540	4.2 (3.0-5.7)	2.75 (1.42-5.34)	100.0	2.75 (1.42-5.34)	<0.01	0.09
	New South Wales	<6	1519	(supp)	65	15120	4.3 (3.3-5.5)	-				
	Norway & Sweden	<5	558	(supp)	21	5580	3.8 (2.3-5.8)	-				
Digestive system	New Zealand	0	954	-	23	9540	2.4 (1.5-3.6)	-		-		
	New South Wales	<6	1519	(supp)	65	15120	4.3 (3.3-5.5)	-				
	Norway & Sweden	<5	558	(supp)	10	5580	1.8 (0.9-3.3)	-				
Respiratory system	New Zealand	<3	954	(supp)	11	9540	1.2 (0.6-2.1)	0.91 (0.12-6.90)	100.0	0.91 (0.12-6.90)	0.93	0.97
	New South Wales	<6	1519	(supp)	25	15120	1.7 (1.1-2.4)	-				
	Norway & Sweden	0	558	-	0	5580	-	-				
Oro-facial clefts	New Zealand	<3	954	(supp)	21	9540	2.2 (1.4-3.4)	0.47 (0.06-3.52)	100.0	0.47 (0.06-3.52)	0.47	0.94
	New South Wales	<6	1519	(supp)	21	15120	1.4 (0.9-2.1)	-				
	Norway & Sweden	0	558	-	19	5580	3.4 (2.1-5.3)	-				
Nervous system	New Zealand	3	954	3.1 (0.7-9.2)	21	9540	2.2 (1.4-3.4)	1.43 (0.43-4.76)	100.0	1.43 (0.43-4.76)	0.56	0.94
	New South Wales	<6	1519	(supp)	39	15120	2.6 (1.8-3.5)	-				
	Norway & Sweden	0	558	-	<5	5580	(supp)	-				
Abdominal wall	New Zealand	<3	954	(supp)	12	9540	1.3 (0.7-2.2)	0.83 (0.11-6.31)	100.0	0.83 (0.11-6.31)	0.86	0.97
	New South Wales	0	1519	-	13	15120	0.9 (0.5-1.5)	-				
	Norway & Sweden	0	558	-	<5	5580	(supp)	-				
Eye	New Zealand	0	954	-	5	9540	0.5 (0.2-1.2)	-		-		
	New South Wales	0	1519	-	<6	15120	(supp)	-				
	Norway & Sweden	0	558	-	9	5580	1.6 (0.7-3.1)	-				
Ear	New Zealand	0	954	-	<3	9540	(supp)	-		-		

© 2025 Tran DT et al. *JAMA Internal Medicine*.

Overall and subgroups	Country	Exposed *			Unexposed *			Adjusted relative risk (RR) and 95% CI				
									Meta analysis **			
		number	denominator	prevalence per 1000 livebirths (95%CI)	number	denominator	prevalence per 1000 livebirths (95%CI)	Country-specific	Weight	Pooled RR (95%CI)	P-value from Poisson model	Benjamini-Hochberg correct p value
	New South Wales	0	1519	-	<6	15120	(supp)	-				
	Norway & Sweden	0	558	-	<5	5580	(supp)	-				
Others	New Zealand	0	954	-	30	9540	3.1 (2.1-4.5)	-				
	New South Wales	9	1519	5.9 (2.7-11.3)	71	15120	4.7 (3.7-5.9)	1.25 (0.63-2.49)	100.0	1.25 (0.63-2.49)	0.52	0.94
	Norway & Sweden	<5	558	(supp)	20	5580	3.6 (2.2-5.5)	-				
**Bupropion**												
Overall	New Zealand	26	749	34.7 (22.7-50.9)	290	7410	39.1 (34.8-43.9)	0.86 (0.58-1.28)	70.0	0.93 (0.67-1.29)	0.66	0.96
	New South Wales	11	293	37.5 (18.7-67.2)	110	2908	37.8 (31.1-45.6)	1.10 (0.60-2.01)	30.0			
Congenital heart defects	New Zealand	5	749	6.7 (2.2-15.6)	86	7410	11.6 (9.3-14.3)	0.55 (0.21-1.42)	100.0	0.55 (0.21-1.42)	0.22	0.77
	New South Wales	<6	293	(supp)	19	2908	6.5 (3.9-10.2)	-				
Limb anomalies	New Zealand	5	749	6.7 (2.2-15.6)	58	7410	7.8 (5.9-10.1)	0.86 (0.34-2.18)	100.0	0.86 (0.34-2.18)	0.76	0.97
	New South Wales	0	293	-	20	2908	6.9 (4.2-10.6)	-				
Genital organs	New Zealand	7	749	9.4 (3.8-19.3)	53	7410	7.2 (5.4-9.4)	1.38 (0.66-2.90)	100.0	1.38 (0.66-2.90)	0.39	0.94
	New South Wales	<6	293	(supp)	17	2908	5.9 (3.4-9.4)	-				
Kidney and urinary tract	New Zealand	4	749	5.3 (1.5-13.7)	35	7410	4.7 (3.3-6.6)	1.27 (0.53-3.08)	100.0	1.27 (0.53-3.08)	0.59	0.95
	New South Wales	0	293	-	13	2908	4.5 (2.4-7.6)	-				
Digestive system	New Zealand	0	749	-	22	7410	3.0 (1.9-4.5)	-		-		
	New South Wales	<6	293	(supp)	21	2908	7.2 (4.5-11.0)	-				
Respiratory system	New Zealand	<3	749	(supp)	18	7410	2.4 (1.4-3.8)	1.10 (0.26-4.66)	100.0	1.10 (0.26-4.66)	0.90	0.97
	New South Wales	0	293	-	<6	2908	(supp)	-				
Oro-facial clefts	New Zealand	0	749	-	12	7410	1.6 (0.8-2.8)	-		-		
	New South Wales	<6	293	(supp)	<6	2908	(supp)	-				
Nervous system	New Zealand	0	749	-	10	7410	1.4 (0.7-2.5)	-		-		
	New South Wales	<6	293	(supp)	7	2908	2.4 (1.0-5.0)	-				
Abdominal wall	New Zealand	<3	749	(supp)	8	7410	1.1 (0.5-2.1)	1.24 (0.16-9.57)	100.0	1.24 (0.16-9.57)	0.84	0.97
	New South Wales	0	293	-	<6	2908	(supp)	-				
Eye	New Zealand	<3	749	(supp)	<3	7410	(supp)	4.96 (0.46-53.24)	100.0	4.96 (0.46-53.24)	0.19	0.75
	New South Wales	0	293	-	<6	2908	(supp)	-				
Ear	New Zealand	0	749	-	0	7410	-	-		-		
	New South Wales	0	293	-	<6	2908	(supp)	-				
Others	New Zealand	3	749	4.0 (0.8-11.7)	22	7410	3.0 (1.9-4.5)	1.35 (0.41-4.41)	100.0	1.35 (0.41-4.41)	0.62	0.95
	New South Wales	<6	293	(supp)	14	2908	4.8 (2.6-8.1)	-				

© 2025 Tran DT et al. *JAMA Internal Medicine*.

Data are number with an outcome, denominator, prevalence per 1000 live births (95% CI), and adjusted relative risk (95% CI).

*: Unexposed infants were born to women who smoked in the first trimester and were not dispensed a prescribed smoking cessation pharmacotherapy during 90 days before conception and the first trimester. In the matched sample, exposed infants were matched to unexposed infants (1:10) on propensity score and year of conception (New Zealand, New South Wales)/ year of childbirth (Norway and Sweden). For data privacy, data suppression is applied to small counts (<3 for New Zealand, <6 for New South Wales, <5 for Norway and Sweden) and such cells are not included in the pooled relative risk. **: Fixed-effects meta-analysis, using the R package *metafor*.

© 2025 Tran DT et al. *JAMA Internal Medicine*.

eTable 19: Major congenital malformations (overall and subgroups) in liveborn infants exposed to nicotine transdermal patches and fast-acting formulations in the first trimester and unexposed infants, before propensity score matching: country-specific number, prevalence and unadjusted relative risk

Overall and subgroups	Country	Exposed *			Unexposed *			Unadjusted relative risk (95% CI)
		number	denominator	prevalence per 1000 livebirths (95%CI)	number	denominator	Prevalence per 1000 livebirths (95%CI)	
**Transdermal patches only**								
Overall	New Zealand	141	3546	39.8 (33.5-46.9)	3041	89659	33.9 (32.7-35.1)	1.17 (0.99-1.39)
	New South Wales	26	647	40.2 (26.3-58.9)	2552	67379	37.9 (36.4-39.4)	1.06 (0.73-1.55)
Congenital heart defects	New Zealand	32	3546	9.0 (6.2-12.7)	765	67379	8.5 (7.9-9.2)	1.06 (0.74-1.51)
	New South Wales	11	647	17.0 (8.5-30.4)	492	67379	7.3 (6.7-8.0)	2.33 (1.29-4.21)
Limb anomalies	New Zealand	30	3546	8.5 (5.7-12.1)	647	89659	7.2 (6.7-7.8)	1.17 (0.81-1.69)
	New South Wales	6	647	9.3 (3.4-20.2)	561	67379	8.3 (7.7-9.0)	1.11 (0.50-2.48)
Genital organs	New Zealand	21	3546	5.9 (3.7-9.1)	524	89659	5.8 (5.4-6.4)	1.01 (0.66-1.57)
	New South Wales	<6	647	(supp)	415	67379	6.2 (5.6-6.8)	-
Kidney and urinary tract	New Zealand	19	3546	5.4 (3.2-8.4)	322	89659	3.6 (3.2-4.0)	1.49 (0.94-2.37)
	New South Wales	<6	647	(supp)	309	67379	4.6 (4.1-5.1)	-
Digestive system	New Zealand	16	3546	4.5 (2.6-7.3)	219	89659	2.4 (2.1-2.8)	1.85 (1.11-3.07)
	New South Wales	<6	647	(supp)	290	67379	4.3 (3.8-4.8)	-
Respiratory system	New Zealand	7	3546	2.0 (0.8-4.1)	160	89659	1.8 (1.5-2.1)	1.11 (0.52-2.36)
	New South Wales	<6	647	(supp)	107	67379	1.6 (1.3-1.9)	-
Oro-facial clefts	New Zealand	7	3546	2.0 (0.8-4.1)	185	89659	2.1 (1.8-2.4)	0.96 (0.45-2.03)
	New South Wales	0	647	-	106	67379	1.6 (1.3-1.9)	-
Nervous system	New Zealand	4	3546	1.1 (0.3-2.9)	172	89659	1.9 (1.6-2.2)	0.59 (0.22-1.58)
	New South Wales	<6	647	(supp)	167	67379	2.5 (2.1-2.9)	-
Abdominal wall	New Zealand	4	3546	1.1 (0.3-2.9)	100	89659	1.1 (0.9-1.4)	1.01 (0.37-2.75)
	New South Wales	<6	647	(supp)	55	67379	0.8 (0.6-1.1)	-
Eye	New Zealand	<3	3546	(supp)	38	89659	0.4 (0.3-0.6)	0.67 (0.09-4.85)
	New South Wales	0	647	-	32	67379	0.5 (0.3-0.7)	-
Ear	New Zealand	<3	3546	(supp)	11	89659	0.1 (0.1-0.2)	2.30 (0.30-17.80)
	New South Wales	0	647	-	16	67379	0.2 (0.1-0.4)	-
Others	New Zealand	33	9242	3.6 (2.5-5.0)	286	89659	3.2 (2.8-3.6)	1.12 (0.78-1.61)
	New South Wales	15	3546	4.2 (2.4-7.0)	286	89659	3.2 (2.8-3.6)	1.33 (0.79-2.23)
**Fast-acting formulation only (lozenges, gums)**								
Overall	New Zealand	93	2454	37.9 (30.6-46.4)	3041	89659	33.9 (32.7-35.1)	1.12 (0.91-1.37)
Congenital heart defects	New Zealand	30	2454	12.2 (8.3-17.5)	765	89659	8.5 (7.9-9.2)	1.43 (0.99-2.06)
Limb anomalies	New Zealand	22	2454	9.0 (5.6-13.6)	647	89659	7.2 (6.7-7.8)	1.24 (0.81-1.90)
Genital organs	New Zealand	11	2454	4.5 (2.2-8.0)	524	89659	5.8 (5.4-6.4)	0.77 (0.42-1.39)

© 2025 Tran DT et al. *JAMA Internal Medicine*.

Overall and subgroups	Country	Exposed *			Unexposed *			Unadjusted relative risk (95% CI)
		number	denominator	prevalence per 1000 livebirths (95%CI)	number	denominator	Prevalence per 1000 livebirths (95%CI)	
Kidney and urinary tract	New Zealand	8	2454	3.3 (1.4-6.4)	322	89659	3.6 (3.2-4.0)	0.91 (0.45-1.83)
Digestive system	New Zealand	13	2454	5.3 (2.8-9.1)	219	89659	2.4 (2.1-2.8)	2.17 (1.24-3.79)
Respiratory system	New Zealand	5	2454	2.0 (0.7-4.8)	160	89659	1.8 (1.5-2.1)	1.14 (0.47-2.78)
Oro-facial clefts	New Zealand	5	2454	2.0 (0.7-4.8)	185	89659	2.1 (1.8-2.4)	0.99 (0.41-2.40)
Nervous system	New Zealand	3	2454	1.2 (0.3-3.6)	172	89659	1.9 (1.6-2.2)	0.64 (0.20-2.00)
Abdominal wall	New Zealand	<3	2454	(supp)	100	89659	1.1 (0.9-1.4)	0.73 (0.18-2.96)
Eye	New Zealand	<3	2454	(supp)	38	89659	0.4 (0.3-0.6)	0.96 (0.13-7.00)
Ear	New Zealand	0	2454	-	11	89659	0.1 (0.1-0.2)	-
Others	New Zealand	8	2454	3.3 (1.4-6.4)	286	89659	3.2 (2.8-3.6)	1.02 (0.51-2.06)

Data are number with an outcome, denominator, prevalence per 1000 live births (95% CI), and unadjusted relative risk (95% CI). Nicotine fast-acting formulations are available in New Zealand only.

*: Unexposed infants were born to women who smoked in the first trimester and were not dispensed a prescribed smoking cessation pharmacotherapy during 90 days before conception and the first trimester. For data privacy, data suppression is applied to small counts (<3 for New Zealand, <6 for New South Wales).

© 2025 Tran DT et al. *JAMA Internal Medicine*.

7. eAppendix 7: Specific major congenital malformations, based on New Zealand cohort

eTable 20: Specific major congenital malformations in liveborn infants exposed to nicotine replacement therapy, varenicline, bupropion in the first trimester and unexposed infants, based on New Zealand cohort before propensity score matching: number, prevalence and unadjusted relative risk.

Specific major malformation	Exposed *		Unexposed *		Unadjusted relative risk (95% CI)
	number	prevalence per 1000 livebirths (95%CI)	number	prevalence per 1000 livebirths (95%CI)	
**Nicotine replacement therapy, any formulation (9242 exposed, 89659 unexposed)**					
**Congenital heart defects**					
Ventricular septal defect	27	2.9 (1.9-4.3)	257	2.9 (2.5-3.2)	1.02 (0.69-1.52)
Atrial septal defect	39	4.2 (3.0-5.8)	389	4.3 (3.9-4.8)	0.97 (0.70-1.35)
Patent ductus arteriosus in term infants	22	2.4 (1.5-3.6)	202	2.3 (2.0-2.6)	1.06 (0.68-1.64)
Tetralogy and pentalogy of Fallot	5	0.5 (0.2-1.3)	30	0.3 (0.2-0.5)	1.62 (0.63-4.17)
Mitral valve atresia/stenosis	4	0.4 (0.1-1.1)	26	0.3 (0.2-0.4)	1.49 (0.52-4.28)
Coarctation of aorta	<3	(supp)	40	0.5 (0.3-0.6)	0.49 (0.12-2.01)
Atrioventricular septal defect	<3	(supp)	21	0.2 (0.1-0.4)	0.92 (0.22-3.94)
Pulmonary valve stenosis	3	0.3 (0.1-1.0)	27	0.3 (0.2-0.4)	1.08 (0.33-3.55)
Hypoplastic left heart	3	0.3 (0.1-1.0)	17	0.2 (0.1-0.3)	1.71 (0.50-5.84)
Total anomalous pulmonary venous return	<3	(supp)	6	0.1 (0.0-0.2)	1.62 (0.19-13.43)
Common arterial truncus	<3	(supp)	6	0.1 (0.0-0.2)	1.62 (0.19-13.43)
Complete transposition of great arteries	<3	(supp)	50	0.6 (0.4-0.7)	0.19 (0.03-1.40)
Double outlet right ventricle	0	-	21	0.2 (0.1-0.4)	-
Aortic valve atresia/stenosis	0	-	<3	0.0 (0.0-0.1)	-
Single ventricle	0	-	7	0.1 (0.0-0.2)	-
Tricuspid atresia and stenosis	0	-	7	0.1 (0.0-0.2)	-
Hypoplastic right heart	0	-	11	0.1 (0.1-0.2)	-
Ebstein's anomaly	<3	(supp)	7	0.1 (0.0-0.2)	1.39 (0.17-11.26)
**Limb anomalies**					
Club foot, talipes equinovarus	23	2.5 (1.6-3.7)	343	3.8 (3.4-4.3)	0.65 (0.43-0.99)
Polydactyly	17	1.8 (1.1-3.0)	115	1.3 (1.1-1.5)	1.43 (0.86-2.39)
Hip dislocation and/or dysplasia	5	0.5 (0.2-1.3)	20	0.2 (0.1-0.3)	2.43 (0.91-6.46)
Limb reduction defects	5	0.5 (0.2-1.3)	27	0.3 (0.2-0.4)	1.80 (0.69-4.67)
Syndactyly	<3	(supp)	32	0.4 (0.2-0.5)	0.61 (0.15-2.53)
**Genital organs**					
Hypospadias	26	2.8 (1.8-4.1)	172	1.9 (1.6-2.2)	1.47 (0.97-2.22)
Indeterminate sex	0	-	7	0.1 (0.0-0.2)	-
**Oro-facial clefts**					
Cleft lip with or without cleft palate	9	1.0 (0.5-1.9)	68	0.8 (0.6-1.0)	1.28 (0.64-2.57)
Cleft palate	11	1.2 (0.6-2.1)	150	1.7 (1.4-2.0)	0.71 (0.39-1.31)
**Kidney and urinary tract**					
Congenital hydronephrosis	13	1.4 (0.8-2.4)	86	1.0 (0.8-1.2)	1.47 (0.82-2.63)
Multicystic renal dysplasia	6	0.7 (0.2-1.4)	35	0.4 (0.3-0.5)	1.66 (0.70-3.95)
Bilateral renal agenesis including Potter sequence	<3	(supp)	0	-	-
Posterior urethral valve	0	-	0	-	-
Bladder exstrophy and/or epispadias	0	-	<3	0.0 (0.0-0.1)	-
**Digestive system**					
Hirschsprung's disease	7	0.8 (0.3-1.6)	19	0.2 (0.1-0.3)	3.57 (1.50-8.50)
Ano-rectal atresia or/and stenosis	4	0.4 (0.1-1.1)	13	0.1 (0.1-0.3)	2.99 (0.97-9.15)
Atresia of bile ducts	3	0.3 (0.1-1.0)	13	0.1 (0.1-0.3)	2.24 (0.64-7.86)
Diaphragmatic hernia	<3	(supp)	19	0.2 (0.1-0.3)	0.51 (0.07-3.81)
Oesophageal atresia with or without trachea-oesophageal fistula	<3	(supp)	10	0.1 (0.1-0.2)	0.97 (0.12-7.58)
Duodenal atresia or stenosis	<3	(supp)	4	0.0 (0.0-0.1)	2.43 (0.27-21.70)
Atresia or stenosis of other parts of small intestine	0	-	15	0.2 (0.1-0.3)	-
Annular pancreas	<3	(supp)	0	-	-

© 2025 Tran DT et al. *JAMA Internal Medicine*.

Specific major malformation	Exposed *		Unexposed *		Unadjusted relative risk (95% CI)
	number	prevalence per 1000 livebirths (95%CI)	number	prevalence per 1000 livebirths (95%CI)	
**Nervous system**					
Severe microcephaly	4	0.4 (0.1-1.1)	34	0.4 (0.3-0.5)	1.14 (0.41-3.22)
Arhinencephaly and/or holoprosencephaly	<3	(supp)	3	0.0 (0.0-0.1)	6.47 (1.08-38.71)
Neural tube defects	0	-	32	0.4 (0.2-0.5)	-
Hydrocephaly	0	-	20	0.2 (0.1-0.3)	-
Spina bifida	0	-	18	0.2 (0.1-0.3)	-
Anencephaly and similar	0	-	4	0.0 (0.0-0.1)	-
Agenesis of corpus callosum	0	-	5	0.1 (0.0-0.1)	-
Encephalocele and meningocele	0	-	10	0.1 (0.1-0.2)	-
**Varenicline (955 exposed, 65164 unexposed)**					
**Cardiac system**					
Ventricular septal defect	3	3.1 (0.7-9.2)	189	2.9 (2.5-3.3)	1.08 (0.35-3.39)
Atrial septal defect	5	5.2 (1.7-12.2)	307	4.7 (4.2-5.3)	1.11 (0.46-2.69)
Patent ductus arteriosus in term infants	<3	(supp)	149	2.3 (1.9-2.7)	0.92 (0.23-3.70)
Tetralogy and pentalogy of Fallot	0	-	23	0.4 (0.2-0.5)	-
Mitral valve atresia/stenosis	<3	(supp)	18	0.3 (0.2-0.4)	3.79 (0.51-28.40)
Coarctation of aorta	<3	(supp)	29	0.5 (0.3-0.6)	2.35 (0.32-17.27)
Atrioventricular septal defect	0	-	17	0.3 (0.2-0.4)	-
Pulmonary valve stenosis	0	-	20	0.3 (0.2-0.5)	-
Hypoplastic left heart	0	-	12	0.2 (0.1-0.3)	-
Total anomalous pulmonary venous return	0	-	3	0.1 (0.0-0.1)	-
Common arterial truncus	0	-	3	0.1 (0.0-0.1)	-
Complete transposition of great arteries	0	-	36	0.6 (0.4-0.8)	-
Double outlet right ventricle	0	-	15	0.2 (0.1-0.4)	-
Aortic valve atresia/stenosis	0	-	0	-	-
Single ventricle	0	-	5	0.1 (0.0-0.2)	-
Tricuspid atresia and stenosis	0	-	7	0.1 (0.0-0.2)	-
Hypoplastic right heart	0	-	9	0.1 (0.1-0.3)	-
Ebstein's anomaly	0	-	5	0.1 (0.0-0.2)	-
**Limb anomalies**					
Club foot, talipes equinovarus	<3	(supp)	245	3.8 (3.3-4.3)	0.56 (0.14-2.24)
Polydactyly	<3	(supp)	87	1.3 (1.1-1.7)	0.78 (0.11-5.63)
Hip dislocation and/or dysplasia	<3	(supp)	13	0.2 (0.1-0.3)	5.25 (0.69-40.12)
Limb reduction defects	0	-	16	0.3 (0.1-0.4)	-
Syndactyly	0	-	24	0.4 (0.2-0.6)	-
**Genital organs**					
Hypospadias	<3	(supp)	129	2.0 (1.7-2.4)	0.53 (0.07-3.78)
Indeterminate sex	0	-	3	0.1 (0.0-0.1)	-
**Oro-facial clefts**					
Cleft lip with or without cleft palate	<3	(supp)	51	0.8 (0.6-1.0)	1.34 (0.18-9.68)
Cleft palate	<3	(supp)	110	1.7 (1.4-2.0)	0.62 (0.09-4.44)
**Kidney and urinary tract**					
Congenital hydronephrosis	4	4.2 (1.1-10.7)	64	1.0 (0.8-1.3)	4.26 (1.55-11.71)
Multicystic renal dysplasia	<3	(supp)	22	0.3 (0.2-0.5)	3.10 (0.42-23.01)
Bilateral renal agenesis including Potter sequence	0	-	0	-	-
Posterior urethral valve	0	-	0	-	-
Bladder exstrophy and/or epispadias	0	-	<3	(supp)	-
**Digestive system**					
Hirschsprung's disease	0	-	12	0.2 (0.1-0.3)	-
Ano-rectal atresia or/and stenosis	0	-	8	0.1 (0.1-0.2)	-
Atresia of bile ducts	0	-	10	0.2 (0.1-0.3)	-
Diaphragmatic hernia	0	-	14	0.2 (0.1-0.4)	-
Oesophageal atresia with/without trachea-oesophageal fistula	0	-	7	0.1 (0.0-0.2)	-

© 2025 Tran DT et al. *JAMA Internal Medicine*.

Specific major malformation	Exposed *		Unexposed *		Unadjusted relative risk (95% CI)
	number	prevalence per 1000 livebirths (95%CI)	number	prevalence per 1000 livebirths (95%CI)	
Duodenal atresia or stenosis	0	-	3	0.1 (0.0-0.1)	-
Atresia or stenosis of other parts of small intestine	0	-	11	0.2 (0.1-0.3)	-
Annular pancreas	0	-	0	-	-
**Nervous system**					
Severe microcephaly	<3	(supp)	25	0.4 (0.3-0.6)	2.73 (0.37-20.14)
Arhinencephaly and/or holoprosencephaly	0	-	<3	(supp)	-
Neural tube defects	0	-	26	0.4 (0.3-0.6)	-
Hydrocephaly	<3	(supp)	13	0.2 (0.1-0.3)	5.25 (0.69-40.12)
Spina bifida	0	-	14	0.2 (0.1-0.4)	-
Anencephaly and similar	0	-	3	0.1 (0.0-0.1)	-
Agenesis of corpus callosum	0	-	4	0.1 (0.0-0.2)	-
Encephalocele and meningocele	0	-	9	0.1 (0.1-0.3)	-
**Bupropion (754 exposed, 73598 unexposed)**					
**Cardiac system**					
Ventricular septal defect	4	5.3 (1.5-13.6)	208	2.8 (2.5-3.2)	1.88 (0.70-5.05)
Atrial septal defect	<3	(supp)	333	4.5 (4.1-5.0)	0.59 (0.15-2.35)
Patent ductus arteriosus in term infants	0	-	167	2.3 (1.9-2.6)	-
Tetralogy and pentalogy of Fallot	0	-	26	0.4 (0.2-0.5)	-
Mitral valve atresia/stenosis	0	-	21	0.3 (0.2-0.4)	-
Coarctation of aorta	0	-	32	0.4 (0.3-0.6)	-
Atrioventricular septal defect	0	-	17	0.2 (0.1-0.4)	-
Pulmonary valve stenosis	0	-	24	0.3 (0.2-0.5)	-
Hypoplastic left heart	0	-	15	0.2 (0.1-0.3)	-
Total anomalous pulmonary venous return	0	-	4	0.1 (0.0-0.1)	-
Common arterial truncus	0	-	3	0.0 (0.0-0.1)	-
Complete transposition of great arteries	0	-	41	0.6 (0.4-0.8)	-
Double outlet right ventricle	0	-	17	0.2 (0.1-0.4)	-
Aortic valve atresia/stenosis	0	-	0	-	-
Single ventricle	0	-	7	0.1 (0.0-0.2)	-
Tricuspid atresia and stenosis	0	-	7	0.1 (0.0-0.2)	-
Hypoplastic right heart	0	-	10	0.1 (0.1-0.3)	-
Ebstein’s anomaly	0	-	7	0.1 (0.0-0.2)	-
**Limb anomalies**					
Club foot, talipes equinovarus	<3	(supp)	278	3.8 (3.4-4.3)	0.35 (0.05-2.50)
Polydactyly	<3	(supp)	97	1.3 (1.1-1.6)	1.01 (0.14-7.22)
Hip dislocation and/or dysplasia	<3	(supp)	16	0.2 (0.1-0.4)	6.10 (0.81-46.00)
Limb reduction defects	0	-	21	0.3 (0.2-0.4)	
Syndactyly	0	-	28	0.4 (0.3-0.6)	
**Genital organs**					
Hypospadias	3	4.0 (0.8-11.6)	148	2.0 (1.7-2.4)	1.98 (0.63-6.21)
Indeterminate sex	0	-	4	0.1 (0.0-0.1)	-
**Oro-facial clefts**					
Cleft lip with or without cleft palate	0	-	57	0.8 (0.6-1.0)	-
Cleft palate	0	-	125	1.7 (1.4-2.0)	-
**Kidney and urinary tract**					
Congenital hydronephrosis	0	-	72	1.0 (0.8-1.2)	-
Multicystic renal dysplasia	0	-	24	0.3 (0.2-0.5)	-
Bilateral renal agenesis including Potter sequence	0	-	0	-	-
Posterior urethral valve	0	-	0	-	-
Bladder exstrophy and/or epispadias	0	-	2	0.0 (0.0-0.1)	-
**Digestive system**					
Hirschsprung’s disease	0	-	16	0.2 (0.1-0.4)	-
Ano-rectal atresia or/and stenosis	0	-	8	0.1 (0.1-0.2)	-

© 2025 Tran DT et al. *JAMA Internal Medicine*.

Specific major malformation	Exposed *		Unexposed *		Unadjusted relative risk (95% CI)
	number	prevalence per 1000 livebirths (95%CI)	number	prevalence per 1000 livebirths (95%CI)	
Atresia of bile ducts	0	-	11	0.2 (0.1-0.3)	-
Diaphragmatic hernia	0	-	15	0.2 (0.1-0.3)	-
Oesophageal atresia with or without trachea-oesophageal fistula	0	-	8	0.1 (0.1-0.2)	-
Duodenal atresia or stenosis	0	-	3	0.0 (0.0-0.1)	-
Atresia or stenosis of other parts of small intestine	0	-	11	0.2 (0.1-0.3)	-
Annular pancreas	0	-	0	-	-
**Nervous system**					
Severe microcephaly	0	-	27	0.4 (0.2-0.5)	-
Arhinencephaly and/or holoprosencephaly	0	-	3	0.0 (0.0-0.1)	-
Neural tube defects	0	-	27	0.4 (0.2-0.5)	-
Hydrocephaly	0	-	15	0.2 (0.1-0.3)	-
Spina bifida	0	-	15	0.2 (0.1-0.3)	-
Anencephaly and similar	0	-	3	0.0 (0.0-0.1)	-
Agenesis of corpus callosum	0	-	4	0.1 (0.0-0.1)	-
Encephalocele and meningocele	0	-	9	0.1 (0.1-0.2)	

Data are number with an outcome, prevalence per 1000 live births (95% CI), and unadjusted relative risk (95% CI).
*: Unexposed infants were born to women who smoked in the first trimester and were not dispensed a prescribed smoking cessation pharmacotherapy during 90 days before conception and the first trimester.
For data privacy, data suppression is applied to small counts (n<3).

© 2025 Tran DT et al. *JAMA Internal Medicine*.

eTable 21: Specific major congenital malformations in liveborn infants exposed to nicotine replacement therapy, varenicline, bupropion in the first trimester and unexposed infants, based on New Zealand cohort after propensity score matching: number, prevalence and adjusted relative risk

Specific major malformation	Exposed *		Unexposed *		Adjusted relative risk (95% CI)	P-value from Poisson model	Benjamini-Hochberg correct p-value
	number	prevalence per 1000 livebirths (95%CI)	number	Prevalence per 1000 livebirths (95%CI)			
**Nicotine replacement therapy, any formulation (8678 exposed, 74873 unexposed)**							
**Congenital heart defects**							
Ventricular septal defect	22	2.5 (1.6-3.8)	214	2.9 (2.5-3.3)	0.84 (0.54-1.30)	0.44	0.86
Atrial septal defect	34	3.9 (2.7-5.5)	341	4.6 (4.1-5.1)	0.81 (0.57-1.16)	0.26	0.68
Patent ductus arteriosus in term infants	20	2.3 (1.4-3.6)	172	2.3 (2.0-2.7)	1.04 (0.66-1.65)	0.86	0.94
Tetralogy and pentalogy of Fallot	4	0.5 (0.1-1.2)	27	0.4 (0.2-0.5)	1.38 (0.48-3.92)	0.55	0.86
Mitral valve atresia/stenosis	3	0.4 (0.1-1.0)	20	0.3 (0.2-0.4)	1.36 (0.43-4.26)	0.60	0.89
Coarctation of aorta	<3	(supp)	34	0.5 (0.3-0.6)	0.42 (0.10-1.74)	0.23	0.68
Atrioventricular septal defect	<3	(supp)	18	0.2 (0.1-0.4)	0.95 (0.26-3.49)	0.93	0.95
Pulmonary valve stenosis	<3	(supp)	23	0.3 (0.2-0.5)	0.52 (0.10-2.73)	0.44	0.86
Hypoplastic left heart	<3	(supp)	14	0.2 (0.1-0.3)	1.11 (0.28-4.42)	0.88	0.94
Total anomalous pulmonary venous return	<3	(supp)	5	0.1 (0.0-0.2)	1.41 (0.19-10.28)	0.74	0.94
Common arterial truncus	<3	(supp)	4	0.1 (0.0-0.1)	2.16 (0.24-19.27)	0.49	0.86
Complete transposition of great arteries	0	-	38	0.5 (0.4-0.7)	-		
Double outlet right ventricle	0	-	18	0.2 (0.1-0.4)	-		
Aortic valve atresia/stenosis	0	-	0	-	-		
Single ventricle	0	-	5	0.1 (0.0-0.2)	-		
Tricuspid atresia and stenosis	0	-	7	0.1 (0.0-0.2)	-		
Hypoplastic right heart	0	-	11	0.2 (0.1-0.3)	-		
Ebstein’s anomaly	0	-	7	0.1 (0.0-0.2)	-		
**Limb anomalies**							
Club foot, talipes equinovarus	23	2.7 (1.7-4.0)	277	3.7 (3.3-4.2)	0.78 (0.51-1.20)	0.26	0.68
Polydactyly	16	1.8 (1.1-3.0)	99	1.3 (1.1-1.6)	1.40 (0.81-2.39)	0.23	0.68
Hip dislocation and/or dysplasia	5	0.6 (0.2-1.3)	17	0.2 (0.1-0.4)	2.48 (0.95-6.45)	0.06	0.58
Limb reduction defects	5	0.6 (0.2-1.3)	20	0.3 (0.2-0.4)	2.18 (0.82-5.76)	0.12	0.68
Syndactyly	<3	(supp)	27	0.4 (0.2-0.5)	0.72 (0.17-3.04)	0.66	0.92
**Genital organs**							
Hypospadias	26	3.0 (2.0-4.4)	148	2.0 (1.7-2.3)	1.48 (0.96-2.28)	0.08	0.58
Indeterminate sex	0	-	4	0.1 (0.0-0.1)	-		
**Oro-facial clefts**							
Cleft lip with or without cleft palate	9	1.0 (0.5-2.0)	56	0.8 (0.6-1.0)	1.49 (0.73-3.03)	0.27	0.68
Cleft palate	10	1.2 (0.6-2.1)	129	1.7 (1.4-2.1)	0.70 (0.37-1.32)	0.27	0.68
**Kidney and urinary tract**							
Congenital hydronephrosis	12	1.4 (0.7-2.4)	71	1.0 (0.7-1.2)	1.38 (0.74-2.57)	0.30	0.68
Multicystic renal dysplasia	6	0.7 (0.3-1.5)	22	0.3 (0.2-0.4)	2.49 (0.98-6.34)	0.06	0.58
Bilateral renal agenesis including Potter sequence	<3	(supp)	0	-	-		
Posterior urethral valve	0	-	0	-	-		
Bladder exstrophy and/or epispadias	0	-	<3	(supp)	-		
**Digestive system**							
Hirschsprung’s disease	7	0.8 (0.3-1.7)	15	0.2 (0.1-0.3)	3.69 (1.47-9.28)	0.01	0.26
Ano-rectal atresia or/and stenosis	3	0.4 (0.1-1.0)	8	0.1 (0.1-0.2)	3.24 (0.86-12.18)	0.08	0.58
Atresia of bile ducts	3	0.4 (0.1-1.0)	13	0.2 (0.1-0.3)	1.92 (0.55-6.67)	0.30	0.68
Diaphragmatic hernia	<3	(supp)	17	0.2 (0.1-0.4)	0.49 (0.06-3.92)	0.50	0.86
Oesophageal atresia with or without trachea-oesophageal fistula	<3	(supp)	9	0.1 (0.1-0.2)	0.96 (0.12-7.54)	0.97	0.97
Duodenal atresia or stenosis	<3	(supp)	3	0.0 (0.0-0.1)	2.87 (0.30-27.56)	0.36	0.77
Atresia or stenosis of other parts of small intestine	0	-	12	0.2 (0.1-0.3)	-		
Annular pancreas	<3	(supp)	0	-	-		
**Nervous system**							

© 2025 Tran DT et al. *JAMA Internal Medicine*.

Specific major malformation	Exposed *		Unexposed *		Adjusted relative risk (95% CI)	P-value from Poisson model	Benjamini-Hochberg correct p-value
	number	prevalence per 1000 livebirths (95%CI)	number	Prevalence per 1000 livebirths (95%CI)			
Severe microcephaly	4	0.5 (0.1-1.2)	27	0.4 (0.2-0.5)	1.09 (0.38-3.10)	0.88	0.94
Arhinencephaly and/or holoprosencephaly	<3	(supp)		(supp)	8.63 (1.22-61.12)	0.03	0.58
Neural tube defects	0	-	26	0.4 (0.2-0.5)	-		
Hydrocephaly	0	-	17	0.2 (0.1-0.4)	-		
Spina bifida	0	-	14	0.2 (0.1-0.3)	-		
Anencephaly and similar	0	-	<3	(supp)	-		
Agenesis of corpus callosum	0	-	4	0.1 (0.0-0.1)	-		
Encephalocele and meningocele	0	-	10	0.1 (0.1-0.3)	-		
**Varenicline (954 exposed, 9540 unexposed)**							
**Cardiac system**							
Ventricular septal defect	3	3.1 (0.7-9.2)	36	3.8 (2.6-5.2)	0.92 (0.29-2.92)	0.88	0.94
Atrial septal defect	5	5.2 (1.7-12.2)	51	5.4 (4.0-7.0)	0.92 (0.38-2.24)	0.86	0.94
Patent ductus arteriosus in term infants	<3	(supp)	26	2.7 (1.8-4.0)	0.93 (0.24-3.52)	0.91	0.95
Tetralogy and pentalogy of Fallot	0	-	4	0.4 (0.1-1.1)	-		
Mitral valve atresia/stenosis	<3	(supp)	3	0.3 (0.1-0.9)	3.33 (0.35-31.23)	0.29	0.68
Coarctation of aorta	<3	(supp)	6	0.6 (0.2-1.4)	1.66 (0.20-13.49)	0.63	0.91
Atrioventricular septal defect	0	-	4	0.4 (0.1-1.1)	-		
Pulmonary valve stenosis	0	-	0	-	-		
Hypoplastic left heart	0	-	5	0.5 (0.2-1.2)	-		
Total anomalous pulmonary venous return	0	-	0	-	-		
Common arterial truncus	0	-	0	-	-		
Complete transposition of great arteries	0	-	<3	(supp)	-		
Double outlet right ventricle	0	-	4	0.4 (0.1-1.1)	-		
Aortic valve atresia/stenosis	0	-	0	-	-		
Single ventricle	0	-	<3	(supp)	-		
Tricuspid atresia and stenosis	0	-	<3	(supp)	-		
Hypoplastic right heart	0	-	<3	(supp)	-		
Ebstein's anomaly	0	-	<3	(supp)	-		
**Limb anomalies**							
Club foot, talipes equinovarus	<3	(supp)	31	3.3 (2.2-4.6)	0.65 (0.16-2.65)	0.55	0.86
Polydactyly	<3	(supp)	12	1.3 (0.7-2.2)	0.84 (0.11-6.25)	0.86	0.94
Hip dislocation and/or dysplasia	<3	(supp)	<3	(supp)	5.01 (0.46-54.11)	0.18	0.68
Limb reduction defects	0	-	<3	(supp)	-		
Syndactyly	0	-	4	0.4 (0.1-1.1)	-		
**Genital organs**							
Hypospadias	<3	(supp)	19	2.0 (1.2-3.1)	0.53 (0.07-3.87)	0.53	0.86
Indeterminate sex	0	-	0	-	-		
**Oro-facial clefts**							
Cleft lip with or without cleft palate		(supp)	7	0.7 (0.3-1.5)	1.43 (0.18-11.34)	0.74	0.94
Cleft palate		(supp)	18	1.9 (1.1-3.0)	0.55 (0.07-4.12)	0.56	0.86
**Kidney and urinary tract**							
Congenital hydronephrosis	4	4.2 (1.1-10.7)	12	1.3 (0.7-2.2)	3.34 (1.08-10.29)	0.04	0.58
Multicystic renal dysplasia	<3	(supp)	3	0.3 (0.1-0.9)	3.34 (0.36-31.08)	0.29	0.68
Bilateral renal agenesis including Potter sequence	0	-	0	-	-		
Posterior urethral valve	0	-	0	-	-		
Bladder exstrophy and/or epispadias	0	-	0	-	-		
**Digestive system**							
Hirschsprung's disease	0	-	<3	(supp)	-		
Ano-rectal atresia or/and stenosis	0	-	<3	(supp)	-		
Atresia of bile ducts	0	-	<3	(supp)	-		
Diaphragmatic hernia	0	-	<3	(supp)	-		

© 2025 Tran DT et al. *JAMA Internal Medicine*.

Specific major malformation	Exposed *		Unexposed *		Adjusted relative risk (95% CI)	P-value from Poisson model	Benjamini-Hochberg correct p-value
	number	prevalence per 1000 livebirths (95%CI)	number	Prevalence per 1000 livebirths (95%CI)			
Oesophageal atresia with/without trachea-oesophageal fistula	0	-	<3	(supp)	-		
Duodenal atresia or stenosis	0	-	0	-	-		
Atresia or stenosis of other parts of small intestine	0	-	0	-	-		
Annular pancreas	0	-	0	-	-		
**Nervous system**							
Severe microcephaly	<3	(supp)	8	0.8 (0.4-1.7)	1.25 (0.16-9.91)	0.83	0.94
Arhinencephaly and/or holoprosencephaly	0	-	0	-	-		
Neural tube defects	0	-	4	0.4 (0.1-1.1)	-		
Hydrocephaly	<3	(supp)	<3	(supp)	5.01 (0.46-54.11)	0.18	0.68
Spina bifida	0	-	<3	(supp)	-		
Anencephaly and similar	0	-	0	-	-		
Agenesis of corpus callosum	0	-	<3	(supp)	-		
Encephalocele and meningocele	0	-	<3	(supp)	-		
**Bupropion (749 exposed, 7410 unexposed)**							
**Cardiac system**							
Ventricular septal defect	4	5.3 (1.5-13.7)	22	3.0 (1.9-4.5)	1.79 (0.62-5.16)	0.28	0.68
Atrial septal defect	<3	(supp)	49	6.6 (4.9-8.7)	0.43 (0.11-1.61)	0.21	0.68
Patent ductus arteriosus in term infants	0	-	15	2.0 (1.1-3.3)	-		
Tetralogy and pentalogy of Fallot	0	-	<3	(supp)	-		
Mitral valve atresia/stenosis	0	-	<3	(supp)	-		
Coarctation of aorta	0	-	5	0.7 (0.2-1.6)	-		
Atrioventricular septal defect	0	-	4	0.5 (0.2-1.4)	-		
Pulmonary valve stenosis	0	-	<3	(supp)	-		
Hypoplastic left heart	0	-	<3	(supp)	-		
Total anomalous pulmonary venous return	0	-	<3	(supp)	-		
Common arterial truncus	0	-	0	-	-		
Complete transposition of great arteries	0	-	4	0.5 (0.2-1.4)	-		
Double outlet right ventricle	0	-	<3	(supp)	-		
Aortic valve atresia/stenosis	0	-	0	-	-		
Single ventricle	0	-	0	-	-		
Tricuspid atresia and stenosis	0	-	<3	(supp)	-		
Hypoplastic right heart	0	-	<3	(supp)	-		
Ebstein's anomaly	0	-	0	-	-		
**Limb anomalies**							
Club foot, talipes equinovarus	<3	(supp)	20	2.7 (1.7-4.2)	0.49 (0.06-3.67)	0.48	0.86
Polydactyly	<3	(supp)	13	1.8 (0.9-3.0)	0.77 (0.11-5.59)	0.79	0.94
Hip dislocation and/or dysplasia	<3	(supp)	0	-	-		
Limb reduction defects	0	-	<3	(supp)	-		
Syndactyly	0	-	<3	(supp)	-		
**Genital organs**							
Hypospadias	3	4.0 (0.8-11.7)	27	3.6 (2.4-5.3)	1.18 (0.42-3.33)	0.75	0.94
Indeterminate sex	0	-	<3	(supp)	-		
**Oro-facial clefts**							
Cleft lip with or without cleft palate	0	-	4	0.5 (0.2-1.4)	-		
Cleft palate	0	-	9	1.2 (0.6-2.3)	-		
**Kidney and urinary tract**							
Congenital hydronephrosis	0	-	10	1.4 (0.7-2.5)	-		
Multicystic renal dysplasia	0	-	<3	(supp)	-		
Bilateral renal agenesis including Potter sequence	0	-	0	-	-		
Posterior urethral valve	0	-	0	-	-		

© 2025 Tran DT et al. *JAMA Internal Medicine*.

Specific major malformation	Exposed *		Unexposed *		Adjusted relative risk (95% CI)	P-value from Poisson model	Benjamini-Hochberg correct p-value
	number	prevalence per 1000 livebirths (95%CI)	number	Prevalence per 1000 livebirths (95%CI)			
Bladder exstrophy and/or epispadias	0	-	0	-	-		
**Digestive system**							
Hirschsprung's disease	0	-	<3	(supp)	-		
Ano-rectal atresia or/and stenosis	0	-	<3	(supp)	-		
Atresia of bile ducts	0	-	<3	(supp)	-		
Diaphragmatic hernia	0	-	4	0.5 (0.2-1.4)	-		
Oesophageal atresia with or without trachea-oesophageal fistula	0	-	0	-	-		
Duodenal atresia or stenosis	0	-	0	-	-		
Atresia or stenosis of other parts of small intestine	0	-	<3	(supp)	-		
Annular pancreas	0	-	0	-	-		
**Nervous system**							
Severe microcephaly	0	-	3	0.4 (0.1-1.2)	-		
Arhinencephaly and/or holoprosencephaly	0	-	0	-	-		
Neural tube defects	0	-	<3	(supp)	-		
Hydrocephaly	0	-	<3	(supp)	-		
Spina bifida	0	-	<3	(supp)	-		
Anencephaly and similar	0	-	0	-	-		
Agenesis of corpus callosum	0	-	0	-	-		
Encephalocele and meningocele	0	-	0	-	-		

Data are number with an outcome, denominator, prevalence per 1000 live births (95% CI), and adjusted relative risk (95% CI).
*: Unexposed infants were born to women who smoked in the first trimester and were not dispensed a prescribed smoking cessation pharmacotherapy during 90 days before conception and the first trimester. Exposed infants were matched to unexposed infants (1:10) on propensity score and year of conception.
For data privacy, data suppression is applied to small counts (n<3).

© 2025 Tran DT et al. *JAMA Internal Medicine*.

8. eAppendix 8: Sensitivity analyses

8.1. Sensitivity analyses using different definitions

eTable 22 presents results of the main analyses and results of the two sensitivity analyses (SA).

- The first SA examined the robustness of the findings to the assumption that women who used a smoking cessation pharmacotherapy smoked during the first trimester. In this SA, we restricted exposed infants to those born to women who self-reported that they smoked in the first trimester. We repeated the calculation of propensity score to include quantity of smoking, and repeated the propensity score matching.
- The second SA acknowledged the potential for exposure misclassification due to a concern that women may stop taking a pharmacotherapy upon recognition of their pregnancy. In the second SA, we restricted exposed infants to those born to women who had at least two dispensings with at least one occurring after conception.

eTable 22: Sensitivity analyses: number, prevalence and adjusted relative risk

Major congenital malforms: overall and subgroups	Exposed *			Unexposed *			Adjusted relative risk (95% CI)	
	number	denominator	prevalence per 1000 livebirths (95%CI)	number	denominator	prevalence per 1000 livebirths (95%CI)		
**Nicotine replacement therapy (any formulation)**								
**Overall**								
Main analysis	351	9325	37.6 (33.8-41.8)	2798	81328	34.4 (33.1-35.7)	1.10 (0.98-1.22)	**
SA: Smoking reported	217	5545	39.1 (34.1-44.7)	1737	50381	34.5 (32.9-36.1)	1.13 (0.99-1.30)	**
SA: ≥2 dispensings	103	3096	33.3 (27.2-40.4)	880	26223	33.6 (31.4-35.9)	0.96 (0.79-1.18)	†
**Congenital heart defects**								
Main analysis	82	9325	8.8 (7.0-10.9)	711	81328	8.7 (8.1-9.4)	0.99 (0.79-1.24)	**
SA: Smoking reported	43	5144	8.4 (6.1-11.3)	412	46377	8.9 (8.1-9.8)	0.88 (0.64-1.19)	†
SA: ≥2 dispensings	19	3096	6.1 (3.7-9.6)	214	26223	8.2 (7.1-9.3)	0.67 (0.42-1.07)	†
**Limb anomalies**								
Main analysis	78	9325	8.4 (6.6-10.4)	597	81328	7.3 (6.8-8.0)	1.17 (0.93-1.49)	**
SA: Smoking reported	47	5144	9.1 (6.7-12.2)	328	46377	7.1 (6.3-7.9)	1.32 (0.97-1.80)	†
SA: ≥2 dispensings	22	3096	7.1 (4.5-10.8)	182	26223	6.9 (6.0-8.0)	1.03 (0.66-1.60)	†
**Genital organs**								
Main analysis	57	8678	6.6 (5.0-8.5)	435	74873	5.8 (5.3-6.4)	1.13 (0.86-1.50)	†
SA: Smoking reported	35	5144	6.8 (4.7-9.5)	261	46377	5.6 (5.0-6.4)	1.25 (0.87-1.78)	†
SA: ≥2 dispensings	23	3096	7.4 (4.7-11.2)	156	26223	6.0 (5.1-7.0)	1.24 (0.80-1.94)	†
**Kidney and urinary tract**								
Main analysis	42	8678	4.8 (3.5-6.5)	270	74873	3.6 (3.2-4.1)	1.31 (0.94-1.82)	†
SA: Smoking reported	23	5144	4.5 (2.8-6.7)	170	46377	3.7 (3.1-4.3)	1.20 (0.77-1.86)	†
SA: ≥2 dispensings	14	3096	4.5 (2.5-7.6)	98	26223	3.7 (3.0-4.6)	1.07 (0.59-1.93)	†
**Digestive system**								
Main analysis	33	8678	3.8 (2.6-5.3)	187	74873	2.5 (2.2-2.9)	1.53 (1.05-2.23)	†
SA: Smoking reported	20	5144	3.9 (2.4-6.0)	122	46377	2.6 (2.2-3.1)	1.44 (0.89-2.33)	†
SA: ≥2 dispensings	6	3096	1.9 (0.7-4.2)	83	26223	3.2 (2.5-3.9)	0.64 (0.28-1.45)	†
**Respiratory system**								
Main analysis	17	8678	2.0 (1.1-3.1)	134	74873	1.8 (1.5-2.1)	1.10 (0.67-1.82)	†
SA: Smoking reported	11	5144	2.1 (1.1-3.8)	77	46377	1.7 (1.3-2.1)	1.24 (0.66-2.33)	†
SA: ≥2 dispensings	5	3096	1.6 (0.5-3.8)	45	26223	1.7 (1.3-2.3)	0.90 (0.36-2.22)	†
**Oro-facial clefts**								
Main analysis	16	8678	1.8 (1.1-3.0)	155	74873	2.1 (1.8-2.4)	0.93 (0.56-1.54)	†
SA: Smoking reported	12	5144	2.3 (1.2-4.1)	103	46377	2.2 (1.8-2.7)	1.06 (0.59-1.91)	†
SA: ≥2 dispensings	4	3096	1.3 (0.4-3.3)	59	26223	2.3 (1.7-2.9)	0.61 (0.24-1.56)	†
**Nervous system**								
Main analysis	10	8678	1.2 (0.6-2.1)	132	74873	1.8 (1.5-2.1)	0.64 (0.33-1.22)	†
SA: Smoking reported	9	5144	1.8 (0.8-3.3)	87	46377	1.9 (1.5-2.3)	0.93 (0.46-1.85)	†
SA: ≥2 dispensings	3	3096	1.0 (0.2-2.8)	53	26223	2.0 (1.5-2.6)	0.45 (0.14-1.43)	†
**Abdominal wall**								
Main analysis	11	8678	1.3 (0.6-2.3)	83	74873	1.1 (0.9-1.4)	1.27 (0.67-2.41)	†
SA: Smoking reported	7	5144	1.4 (0.6-2.8)	54	46377	1.2 (0.9-1.5)	1.27 (0.58-2.79)	†
SA: ≥2 dispensings	6	3096	1.9 (0.7-4.2)	37	26223	1.4 (1.0-1.9)	1.62 (0.67-3.91)	†

© 2025 Tran DT et al. *JAMA Internal Medicine*.

Major congenital malforms: overall and subgroups	Exposed *			Unexposed *			Adjusted relative risk (95% CI)	
	number	denominator	prevalence per 1000 livebirths (95%CI)	number	denominator	prevalence per 1000 livebirths (95%CI)		
**Eye**								
Main analysis	7	8678	0.8 (0.3-1.7)	30	74873	0.4 (0.3-0.6)	2.06 (0.92-4.63)	†
SA: Smoking reported	5	5144	1.0 (0.3-2.3)	18	46377	0.4 (0.2-0.6)	2.51 (0.93-6.73)	†
SA: ≥2 dispensings	4	3096	1.3 (0.4-3.3)	12	26223	0.5 (0.2-0.8)	3.33 (1.10-10.02)	†
**Ear**								
Main analysis	<3	8678	(supp)	8	74873	0.1 (0.1-0.2)	1.08 (0.14-8.60)	†
SA: Smoking reported	0	5144	-	5	46377	0.1 (0.0-0.3)	-	†
SA: ≥2 dispensings	<3	3096	(supp)	3	26223	0.1 (0.0-0.3)	2.82 (0.30-26.97)	†
**Others**								
Main analysis	31	8678	3.6 (2.4-5.1)	238	74873	3.2 (2.8-3.6)	1.12 (0.77-1.63)	†
SA: Smoking reported	19	5144	3.7 (2.2-5.8)	146	46377	3.2 (2.7-3.7)	1.15 (0.72-1.85)	†
SA: ≥2 dispensings	11	3096	3.6 (1.8-6.4)	84	26223	3.2 (2.6-4.0)	1.09 (0.59-2.04)	†
**Varenicline**								
**Overall**								
Main analysis	99	3031	32.7 (26.6-39.8)	1106	30240	36.6 (34.5-38.8)	0.90 (0.73-1.10)	‡
SA: Smoking reported	30	1095	27.4 (18.5-39.1)	386	10927	35.3 (31.9-39.0)	0.76 (0.52-1.09)	**
SA: ≥2 dispensings	9	249	36.1 (16.5-68.6)	85	2490	34.1 (27.3-42.2)	1.02 (0.50-2.10)	†
**Congenital heart defects**								
Main analysis	27	3031	8.9 (5.9-13.0)	294	30240	9.7 (8.6-10.9)	0.99 (0.67-1.47)	‡
SA: Smoking reported	4	464	8.6 (2.4-22.1)	42	4640	9.1 (6.5-12.2)	0.94 (0.34-2.62)	†
SA: ≥2 dispensings	3	249	12.1 (2.5-35.2)	29	2490	11.7 (7.8-16.7)	0.99 (0.35-2.86)	†
**Limb anomalies**								
Main analysis	15	2473	6.1 (3.4-10.0)	183	24660	7.4 (6.4-8.6)	0.85 (0.51-1.42)	**
SA: Smoking reported	9	1095	8.2 (3.8-15.6)	80	10927	7.3 (5.8-9.1)	1.24 (0.64-2.41)	**
SA: ≥2 dispensings	<3	249	(supp)	19	2490	7.6 (4.6-11.9)	0.54 (0.08-3.61)	†
**Kidney and urinary tract**								
Main analysis	11	954	11.5 (5.8-20.6)	40	9540	4.2 (3.0-5.7)	2.75 (1.42-5.34)	†
SA: Smoking reported	<3	464	(supp)	21	4640	4.5 (2.8-6.9)	0.95 (0.23-3.91)	†
SA: ≥2 dispensings	5	249	20.1 (6.5-46.9)	7	2490	2.8 (1.1-5.8)	7.19 (2.38-21.79)	†
**Bupropion**								
**Overall**								
Main analysis	37	1042	35.5 (25.0-48.9)	400	10318	38.8 (35.1-42.8)	0.93 (0.67-1.29)	**
SA: Smoking reported	27	522	51.7 (34.1-75.3)	192	5207	36.9 (31.8-42.5)	1.46 (0.98-2.13)	**
SA: ≥2 dispensings	5	128	39.1 (12.7-91.2)	63	1271	49.6 (38.1-63.4)	0.67 (0.27-1.68)	†
**Congenital heart defects**								
Main analysis	5	749	6.7 (2.2-15.6)	86	7410	11.6 (9.3-14.3)	0.55 (0.21-1.42)	†
SA: Smoking reported	3	369	8.1 (1.7-23.8)	46	3690	12.5 (9.1-16.6)	0.64 (0.20-2.10)	†
SA: ≥2 dispensings	<3	128	(supp)	20	1271	15.7 (9.6-24.3)	0.37 (0.03-4.85)	†
**Genital organs**								
Main analysis	7	749	9.4 (3.8-19.3)	53	7410	7.2 (5.4-9.4)	1.38 (0.66-2.90)	†
SA: Smoking reported	5	369	13.6 (4.4-31.6)	28	3690	7.6 (5.0-11.0)	1.81 (0.71-4.62)	†
SA: ≥2 dispensings	<3	128	(supp)	6	1271	4.7 (1.7-10.3)	3.26 (0.76-14.02)	†
**Kidney and urinary tract**								
Main analysis	4	749	5.3 (1.5-13.7)	35	7410	4.7 (3.3-6.6)	1.27 (0.53-3.08)	†
SA: Smoking reported	3	369	8.1 (1.7-23.8)	19	3690	5.2 (3.1-8.0)	1.80 (0.62-5.18)	†
SA: ≥2 dispensings	0	128	-	11	1271	8.7 (4.3-15.5)	-	†

Data are number with an outcome, denominator, prevalence per 1000 livebirths (95%CI), and adjusted relative risk (95% CI).

*: Exposed babies were matched to unexposed babies (1:10) using propensity score and calendar year. Unexposed infants were born to women who smoked in the first trimester and were not dispensed a prescribed smoking cessation pharmacotherapy during 90 days before conception and the first trimester.

**: Estimates pooled from New Zealand and New South Wales cohorts.

†: Estimates based on New Zealand cohort; for data privacy, counts <3 and prevalence are suppressed.

‡: Estimates pooled across New Zealand, New South Wales, Norway and Sweden cohorts.

§: Estimates based on New South Wales cohort.

© 2025 Tran DT et al. *JAMA Internal Medicine*.

8.2. Quantifying potential impacts of selecting livebirths

In the base cohort of 391,474 births, the overall proportion of stillbirths was 0.63% (n=2476). Among births exposed to smoking cessation pharmacotherapy (prior to propensity score matching), the proportion of stillbirths was 0.5% [50 of 9940] in NRT-exposed births, 1.0% [30 of 3062] in varenicline-exposed births, and 0.9% [10 of 1057] in bupropion-exposed births.

Malformation outcomes in this study were assessed among livebirths only, with non-livebirths (i.e. spontaneous abortion, termination, or stillbirth) were excluded from the analysis. Severe malformation may cause non-livebirths. If non-livebirths occurred more often in women who used a smoking cessation pharmacotherapy, the risk of malformation based solely on livebirths may be underestimated. In this sensitivity analyses, we evaluated the potential bias due to inclusion of only livebirths by estimating plausible adjusted relative risks under scenarios where pregnancies with a MCM resulted in miscarriage or termination instead of livebirth.

We defined the following probabilities of livebirths being included in the study (selection probability).[22]

	With malformation	Without malformation
Exposed	S11	S01
Unexposed	S10	S00

Among unexposed pregnancies:

- S00 denotes probability of selection of livebirths without a malformation in the study. About 48% of pregnancies are unintended and the proportion of unintended pregnancies ending in abortion is 43%.[23] Only a small proportion of these terminated unintended pregnancies would carry a fetus with a major congenital malformation. Therefore, around 20% of pregnancies (0.48*0.43) are terminated for reasons other than a recognised malformation. Hence, S00 is assumed to be 80%.

- S10 demotes probability of selection of livebirths with a malformation: It is reported that for many types of birth defects, about 10% of affected pregnancies are ended by a termination.[24] Adding a 20% probability of termination for reasons other than a recognised malformation yields a total 30% for probability of non-livebirths among malformed foetuses. To be conservative, we evaluated the impact of higher probabilities of non-livebirths among affected pregnancies, range 30%-50%. Hence, S10 is assumed to vary between 50% and 70%.

Among exposed pregnancies:

- S01 denotes probability of selection of livebirths without a malformation. The value of S01 is based on the S00 and would decrease or increase by up to 20%
  S01=S00 +N%, with N ranging from -20% to +20%

- S11 denotes probability of selection of livebirths with a malformation. The value of S11 is based on the S10 and would decrease or increase by up to 20%.
  S11=S10 +N%, with N ranging from -20% to +20%

Corrected adjusted relative risk (aRR) was calculated using equation: Corrected RR = original aRR* $\frac{S10*S01}{S11*S00}$

eFigures 1-3 showed the range of plausible values of corrected aRRs for effects of exposure to NRT (any formulation), varenicline and bupropion, respectively. The most extreme scenarios represented S10 ≤50% (i.e. probability of non-livebirths being greater than 50% among unexposed pregnancies), S11 ≤30% (i.e. probability of non-livebirths being greater than 70% among exposed pregnancies), with S01=60% and S00=80%.

With regard to the effects of exposure to NRT (any formulation), under the most extreme scenarios, the observed RR would shift from 1.10 to 1.38 for outcome MCMs overall, 0.99 to 1.24 for congenital heart defects, 1.17 to 1.46 for limb anomalies, 1.13 to 1.41 for genital organ malformations, 1.31 to 1.64 for kidney and urinary tract malformations, and 1.51 to 1.89 for digestive system malformations (eFigure 1).

With regard to the effects of exposure to varenicline, under the most extreme scenarios, the observed RR would shift from 0.90 to 1.13 for MCMs overall, 0.99 to 1.24 for congenital heart defects, 0.85 to 1.06 for limb anomalies, 1.26 to 1.58 for genital organ malformations, and 2.75 to 3.44 for kidney and urinary tract malformation (eFigure 2).

Regarding bupropion, the most extreme scenarios would shift RR from 0.93 to 1.16 for MCMs overall (eFigure 3).

© 2025 Tran DT et al. *JAMA Internal Medicine*.

eFigure 1: Plausible values for corrected relative risks for the effects of exposure to nicotine replacement therapy (any formulation) in the first trimester and major congenital malformations.

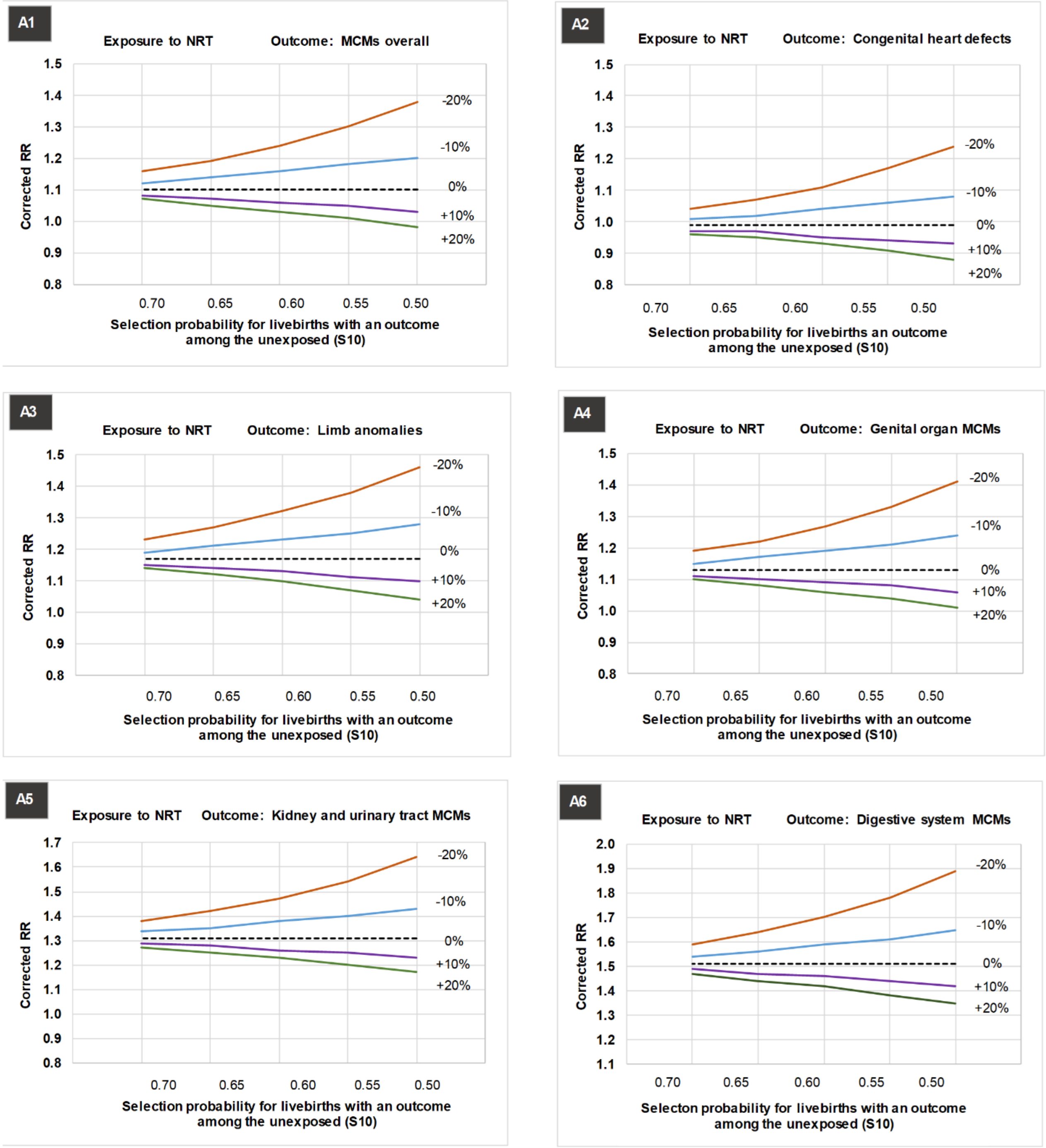

The x-axis shows a range of S10 - selection probabilities for livebirths with a malformation among unexposed pregnancies. The different lines represent the corrected RR for a range of S11 - selection probabilities for livebirths among exposed pregnancies (S11=S10 +N%, with N ranging from -20% to +20%).

© 2025 Tran DT et al. *JAMA Internal Medicine*.

eFigure 2: Plausible values for corrected relative risks (RR) for the effects of exposure to varenicline in the first trimester and major congenital malformations (MCMs).

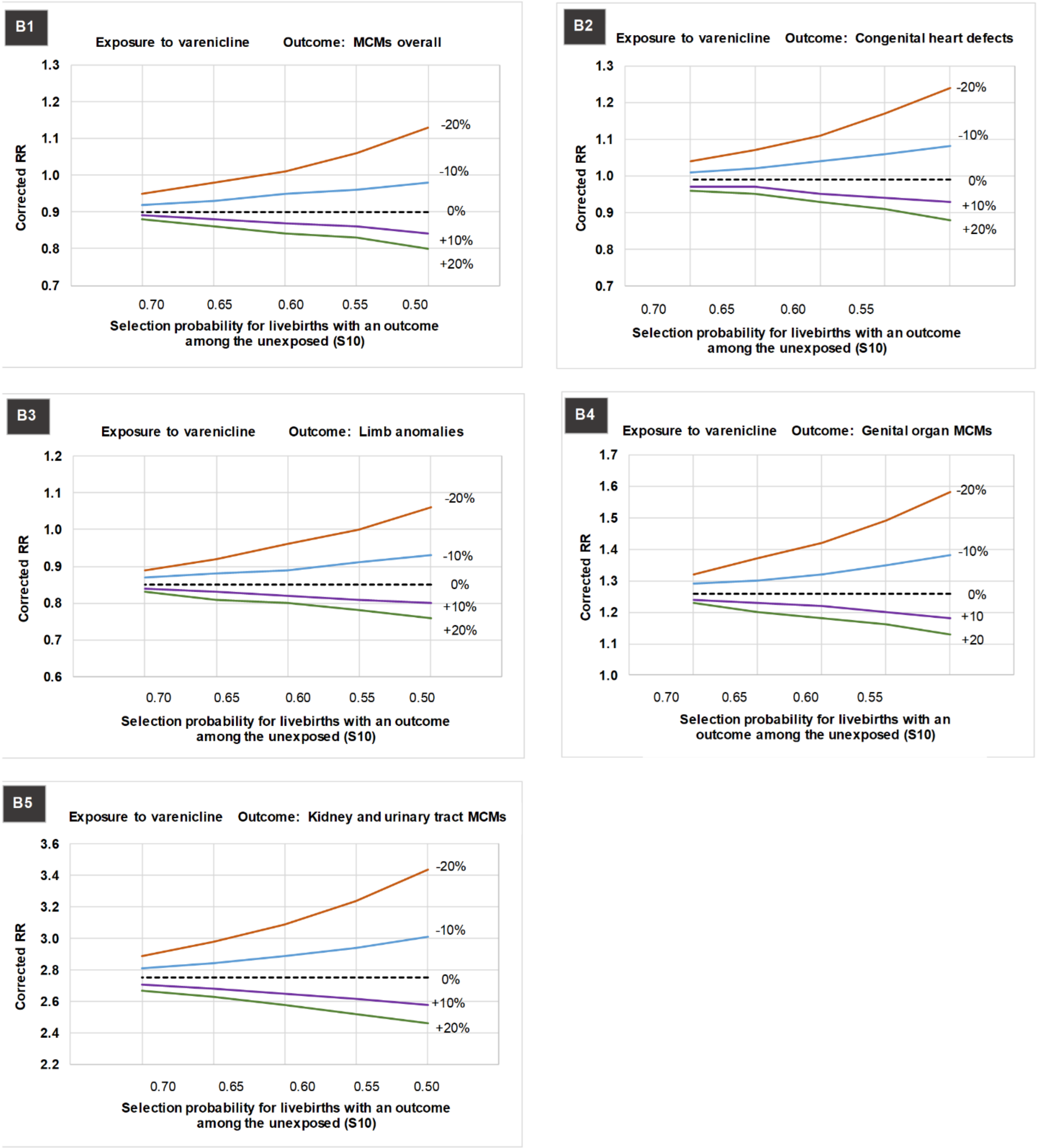

The x-axis shows a range of S10 - selection probabilities for livebirths with a malformation among unexposed pregnancies. The different lines represent the corrected RR for a range of S11 - selection probabilities for livebirths among exposed pregnancy (S11=S10 +N%, with N ranging from -20% to +20%).

© 2025 Tran DT et al. *JAMA Internal Medicine*.

eFigure 3: Plausible values for corrected relative risks (RR) for the effects of exposure to bupropion in the first trimester and major congenital malformations (MCMs) overall.

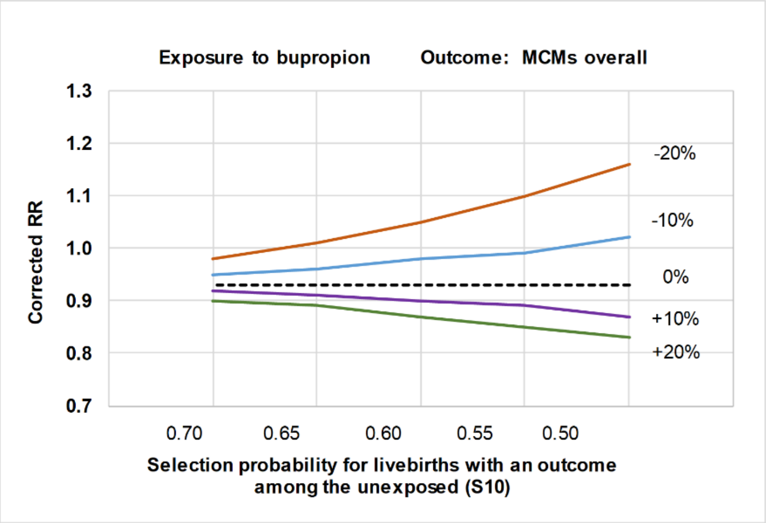

The x-axis shows a range of S10 - selection probabilities for livebirths with a malformation among unexposed pregnancies. The different lines represent the corrected RR for a range of S11 - selection probabilities for livebirths among exposed pregnancy (S11=S10 +N%, with N ranging from -20% to +20%).

8.3. Quantifying potential impacts of unmeasured confounding

In addition to covariates that we measured and included in the propensity score matching, several unmeasured factors such as alcohol consumption, changes in lifestyle behaviours connected to motivation to quit smoking, and occupational and environmental factors that may confound the association between exposure to a smoking cessation pharmacotherapy and major congenital malformations. Individual-level data on these potential confounding factors were not available to our study; however, using the E-value method we can assess the potential impacts of an unmeasured confounding factor.[25, 26] E-value is the minimum strength of association that an unmeasured confounder should have with both the exposure and the outcome to explain away the observed association.[25, 26] The E-values for MCM subgroups for which elevated risks were observed are shown in eTable 23.

eTable 23: E-values for the risk of MCM subgroups with potential signals following exposure to NRT and varenicline

Major congenital malformation subgroups	Smoking cessation pharmacotherapy	E- value
Digestive system	NRT	2.43
Eye	NRT	3.54
Kidney and urinary tract	Varenicline	4.94

© 2025 Tran DT et al. *JAMA Internal Medicine*.

9. eReferences

1. Donald S, Barson D, Horsburgh S, Sharples K, Parkin L. Generation of a pregnancy cohort for medicine utilisation and medicine safety studies in New Zealand. *Pharmacoepidemiol Drug Saf*. 2018;27(12):1335-1343. doi:10.1002/pds.4671
2. Tran DT, Robijn AL, Varney B, et al. Data Resource Profile: The Early Life Course data platform for research on perinatal and early childhood exposures and outcomes in Australia. *Int J Epidemiol*. 2024;53(2). doi:10.1093/ije/dyae045
3. Cohen JM, Cesta CE, Kjerpeseth L, et al. A common data model for harmonization in the Nordic Pregnancy Drug Safety Studies (NorPreSS). *Norsk Epidemiologi*. 2021;29(1-2):117-123. doi:10.5324/nje.v29i1-2.4053
4. Furu K, Wettermark B, Andersen M, Martikainen JE, Almarsdottir AB, Sørensen HT. The Nordic countries as a cohort for pharmacoepidemiological research. *Basic Clin Pharmacol Toxicol*. 2010;106(2). doi:10.1111/j.1742-7843.2009.00494.x
5. Cnattingius S, Källén K, Sandström A, et al. The Swedish medical birth register during five decades: documentation of the content and quality of the register. *Eur J Epidemiol*. 2023;38(1):109-120. doi:10.1007/s10654-022-00947-5
6. Ludvigsson JF, Andersson E, Ekbom A, et al. External review and validation of the Swedish national inpatient register. *BMC Public Health*. 2011;11. doi:10.1186/1471-2458-11-450
7. Irgens LM. The Medical Birth Registry of Norway. Epidemiological research and surveillance throughout 30 years. *Acta Obstet Gynecol Scand*. 2000;79(6). doi:10.1034/j.1600-0412.2000.079006435.x
8. Ludvigsson JF, Håberg SE, Knudsen GP, et al. Ethical aspects of registry-based research in the Nordic countries. *Clin Epidemiol*. 2015;7.
9. Johansson LA, Westerling R. Comparing Swedish hospital discharge records with death certificates: Implications for mortality statistics. *Int J Epidemiol*. 2000;29(3). doi:10.1093/intjepid/29.3.495
10. Ludvigsson JF, Svedberg P, Olén O, Bruze G, Neovius M. The longitudinal integrated database for health insurance and labour market studies (LISA) and its use in medical research. *Eur J Epidemiol*. Published online 2019. doi:10.1007/s10654-019-00511-8
11. Tran DT, Preen DB, Einarsdottir K, et al. Use of smoking cessation pharmacotherapies during pregnancy is not associated with increased risk of adverse pregnancy outcomes: a population-based cohort study. *BMC Med*. 2020;18(1):15. doi:10.1186/s12916-019-1472-9
12. Roper L, Tran DT, Einarsdóttir K, Preen DB, Havard A. Algorithm for resolving discrepancies between claims for smoking cessation pharmacotherapies during pregnancy and smoking status in delivery records: The impact on estimates of utilisation. *PLoS One*. 2018;13(8):e0202999. doi:10.1371/journal.pone.0202999
13. Robijn AL, Tran DT, Cohen JM, et al. Smoking cessation pharmacotherapy use in pregnancy. *JAMA Netw Open*. Published online 2024.
14. Pharmaceutical Benefits Scheme. *Post-Market Review of Medicines for Smoking Cessation – Final Report to the PBAC.*; 2022.
15. European Surveillance of Congenital Anomalies. EUROCAT Guide 1.4 and reference documents (Version 28/12/2018). Section 3.3. EUROCAT subgroups of congenital anomalies. 2018. https://eu-rd-platform.jrc.ec.europa.eu/sites/default/files/Full_Guide_1_4_version_28_DEC2018.pdf
16. European Surveillance of Congenital Anomalies. EUROCAT prevalence data tables. Cases and prevalence (per 10,000 births) of all congenital anomaly subgroups for all registries,

© 2025 Tran DT et al. *JAMA Internal Medicine*.

from 2011-2021. 2022. https://eu-rd-platform.jrc.ec.europa.eu/eurocat/eurocat-data/prevalence/export_en
17. Petersen JM, Yazdy MM, Getz KD, Anderka MT, Werler MM. Short interpregnancy intervals and risks for birth defects: support for the nutritional depletion hypothesis. *Am J Clin Nutr*. 2021;113(6):1688-1699. doi:10.1093/ajcn/nqaa436
18. Bateman BT, Heide-Jørgensen U, Einarsdóttir K, et al. β-Blocker use in pregnancy and the risk for congenital malformations: an international cohort study. *Ann Intern Med*. 2018;169(10):665-673. doi:10.7326/m18-0338
19. Hálfdánarson Ó, Cohen JM, Karlstad Ø, et al. Antipsychotic use in pregnancy and risk of attention/deficit-hyperactivity disorder and autism spectrum disorder: a Nordic cohort study. 2022;25(2):54-62. doi:10.1136/ebmental-2021-300311 %J Evidence Based Mental Health
20. Australian Therapeutic Goods Administration. The Australian categorisation system for prescribing medicines in pregnancy. Published online 2022. https://www.tga.gov.au/products/medicines/find-information-about-medicine/prescribing-medicines-pregnancy-database
21. Pratt NL, Kerr M, Barratt JD, et al. The validity of the Rx-Risk Comorbidity Index using medicines mapped to the Anatomical Therapeutic Chemical (ATC) Classification System. *BMJ Open*. 2018;8(4):e021122. doi:10.1136/bmjopen-2017-021122
22. Huybrechts KF, Palmsten K, Avorn J, et al. Antidepressant use in pregnancy and the risk of cardiac defects. *New England Journal of Medicine*. 2014;370(25):2397-2407. doi:10.1056/NEJMoa1312828
23. Bearak J, Popinchalk A, Ganatra B, et al. Unintended pregnancy and abortion by income, region, and the legal status of abortion: estimates from a comprehensive model for 1990–2019. *Lancet Glob Health*. 2020;8(9). doi:10.1016/S2214-109X(20)30315-6
24. Svensson E, Ehrenstein V, Nørgaard M, et al. Estimating the proportion of all observed birth defects occurring in pregnancies terminated by a second-trimester abortion. *Epidemiology*. 2014;25(6). doi:10.1097/EDE.0000000000000163
25. Van Der Weele TJ, Ding P. Sensitivity analysis in observational research: Introducing the E-Value. *Ann Intern Med*. 2017;167(4):268-274. doi:10.7326/M16-2607
26. Gaster T, Eggertsen CM, Støvring H, Ehrenstein V, Petersen I. Quantifying the impact of unmeasured confounding in observational studies with the E value. *BMJ Medicine*. 2023;2(1). doi:10.1136/bmjmed-2022-000366

© 2025 Tran DT et al. *JAMA Internal Medicine*.

10. STROBE Statement

Checklist according to the Strengthening the Reporting of Observational Studies in Epidemiology (STROBE) guidelines. Checklist of items that should be included in reports of cohort studies

	Item No	Recommendation	Manuscript section and page
**Title and abstract**	1	(*a*) Indicate the study's design with a commonly used term in the title or the abstract	• Title "Risk of major congenital malformations following prenatal exposure to smoking cessation medicines"
		(*b*) Provide in the abstract an informative and balanced summary of what was done and what was found	• The abstract includes informative subheadings Importance, Objective, Design, Setting, Participants, Exposure, Main outcomes, Results, and Conclusion.<br>• Key points panel is included, with subheadings: Question, Findings and Meaning.
**Introduction**			
Background/ rationale	2	Explain the scientific background and rationale for the investigation being reported	• Scientific background and rationales for the study are presented in page 6
Objectives	3	State specific objectives, including any prespecified hypotheses	• Statements about the aims of the paper are included: "To overcome the statistical power and confounding by maternal smoking issues present in prior studies, we conducted a large cohort study across four countries to assess the risk of MCMs overall, in subgroups, and specific malformations associated with maternal use of NRT, varenicline and bupropion in the first trimester compared to smoking. We also estimated the risk of MCMs separately for NRT transdermal patches and fast-acting formulations."
**Methods**			
Study design	4	Present key elements of study design early in the paper	• A sentence to describe study design is included "We conducted a retrospective cohort study, using a common protocol in New Zealand (NZ), Norway, Sweden, and Australia (only New South Wales [NSW], the most populous state in Australia)".
Setting	5	Describe the setting, locations, and relevant dates, including periods of recruitment, exposure, follow-up, and data collection	• The setting, locations, and relevant dates are described in sub-heading Data sources and study cohort (Page 7).
Participants	6	(*a*) Give the eligibility criteria, and the sources and methods of selection of participants. Describe methods of follow-up	• Description about eligible pregnancies is provided in sub-heading "Data sources and study cohort".<br>• Definitions of follow-up are provided under the sub-heading Outcomes.
		(*b*) For matched studies, give matching criteria and number of exposed and unexposed	• Matching criteria and number of exposed and unexposed pregnancies are described in sub-heading "Statistical analyses", and Figure 1
Variables	7	Clearly define all outcomes, exposures, predictors, potential confounders, and effect modifiers. Give diagnostic criteria, if applicable	• Definitions of outcomes are described in sub-heading Outcomes, and the Appendices<br>• Definition of potential confounders are stated in sub-heading Statistical analyses and the Appendices.
Data sources/ measurement	8*	For each variable of interest, give sources of data and details of methods of assessment (measurement). Describe comparability of assessment methods if there is more than one group	• Descriptions of data sources and methods to derive study variables are presented in sub-heading Study design and population and the Appendix.
Bias	9	Describe any efforts to address potential sources of bias	• Propensity score matching was used to address potential confounding. Description of propensity score matching is presented in section Statistical analyses.
Study size	10	Explain how the study size was arrived at	• Description of the study size is provided in the sub-heading Study design and population, Figure 1 and the Appendix.
Quantitative variables	11	Explain how quantitative variables were handled in the analyses. If applicable, describe which groupings were chosen and why	• Full detailed descriptions of data sources, study outcomes, potential confounding factors, methods to derive study variables are presented in the Appendix.
Statistical methods	12	(*a*) Describe all statistical methods, including those used to control for confounding	• Section Statistical analyses describes descriptive and comparative statistical methods.
		(*b*) Describe any methods used to examine subgroups and interactions	• Selection of exposed and unexposed pregnancies is described in the sub-heading Exposure and in the Appendix.
		(*c*) Explain how missing data were addressed	• Full detailed descriptions of data sources and variables are provided in the Appendix. Missing gestational age is excluded.
		(*d*) If applicable, explain how loss to follow-up was addressed	• Not applicable in this study.

© 2025 Tran DT et al. *JAMA Internal Medicine*.

	Item No	Recommendation	Manuscript section and page
		(*e*) Describe any sensitivity analyses	• Series of sensitivity analyses were described in the statistical methods. Results of these analyses are presented in the Result sections and in the Appendix
**Results**			
Participants	13*	(a) Report numbers of individuals at each stage of study—eg numbers potentially eligible, examined for eligibility, confirmed eligible, included in the study, completing follow-up, and analysed	• Information about the number of people identified in each data source, number of pregnancies excluded and included in the analyses is provided in Figure 1, Table 1 and the Appendix.
		(b) Give reasons for non-participation at each stage	• This is not applicable in the current manuscript.
		(c) Consider use of a flow diagram	• A flow diagram is included in Figure 1.
Descriptive data	14*	(a) Give characteristics of study participants (eg demographic, clinical, social) and information on exposures and potential confounders	• Characteristics of exposed and unexposed pregnancies are presented in the section Results, Table 1 and the Appendix.
		(b) Indicate number of participants with missing data for each variable of interest	• Not applicable
		(c) Summarise follow-up time (eg, average and total amount)	• Follow-up was 18 months in Australia and New Zealand cohorts and 12 months in Norway and Sweden cohorts.
Outcome data	15*	Report numbers of outcome events or summary measures over time	• Outcomes are reported in the section Results, Tables 2-4 and the Appendix.
Main results	16	(*a*) Give unadjusted estimates and, if applicable, confounder-adjusted estimates and their precision (eg, 95% confidence interval). Make clear which confounders were adjusted for and why they were included	• Adjusted results are provided in the section Results, Tables 2-4 and the Appendix.
		(*b*) Report category boundaries when continuous variables were categorized	• Categorisation of continuous variables is described in the Appendix.
		(*c*) If relevant, consider translating estimates of relative risk into absolute risk for a meaningful time period	• Not applicable
Other analyses	17	Report other analyses done—eg analyses of subgroups and interactions, and sensitivity analyses	• Subgroup analyses are not applicable to this study
**Discussion**			
Key results	18	Summarise key results with reference to study objectives	• Principal findings are summarised in the section Discussion .
Limitations	19	Discuss limitations of the study, taking into account sources of potential bias or imprecision. Discuss both direction and magnitude of any potential bias	• Study strengths and limitations are discussed in page 15.
Interpretation	20	Give a cautious overall interpretation of results considering objectives, limitations, multiplicity of analyses, results from similar studies, and other relevant evidence	• Cautions in interpretation of results are discussed in page 14&15.
Generalisability	21	Discuss the generalisability (external validity) of the study results	• Generalisability of study results are discussed in page 15.
**Other information**			
Funding	22	Give the source of funding and the role of the funders for the present study and, if applicable, for the original study on which the present article is based	• Sources of funding and the role of the funding bodies are declared.

© 2025 Tran DT et al. *JAMA Internal Medicine*.
