# Supplementary material for: Risk of Major Congenital Malformations Following Prenatal Exposure to Smoking Cessation Medicines
Source: JAMA Intern Med. 2025 Mar 31;185(6):656–67. doi: 10.1001/jamainternmed.2025.0290 (PMC11959474; doi:10.1001/jamainternmed.2025.0290)
Supplement: Supplement 2. — Data sharing statement [file jamainternmed-e250290-s002.pdf]

Data Sharing Statement

Tran. Risk of Major Congenital Malformations Following Prenatal Exposure to Smoking Cessation Medicines. *JAMA Intern Med*. Published March 31, 2025.
doi:10.1001/jamainternmed.2025.0290

Data
Data available: No

Additional Information
Explanation for why data not available: The datasets generated or analysed, or both, during this study are not publicly available because of governance restrictions.
